# ATP-citrate lyase controls endothelial gluco-lipogenic metabolism and vascular inflammation in sepsis-associated organ injury

**DOI:** 10.1038/s41419-023-05932-8

**Published:** 2023-07-06

**Authors:** Ranran Li, Mei Meng, Ying Chen, Tingting Pan, Yinjiaozhi Li, Yunxin Deng, Ruyuan Zhang, Rui Tian, Wen Xu, Xiangtao Zheng, Fangchen Gong, Jie Liu, Haiting Tang, Xiaowei Ding, Yaoqing Tang, Djillali Annane, Erzhen Chen, Hongping Qu, Lei Li

**Affiliations:** 1 Department of Critical Care Medicine, Ruijin Hospital, Shanghai Jiao Tong University School of Medicine, Shanghai, P.R. China; 2 Department of Emergency, Ruijin Hospital, Shanghai Jiao Tong University School of Medicine, Shanghai, P.R. China; 3 National Advanced Medical Engineering Research Center, China State Institute of Pharmaceutical Industry, Shanghai, P.R. China; 4 Department of Obstetrics and Gynecology, Ruijin Hospital, Shanghai Jiao Tong University School of Medicine, Shanghai, P.R. China; 5 General intensive care unit, Raymond Poincaré Hospital (APHP), Laboratory of Inflammation and Infection U1173, University of Versailles SQY/INSERM 104 bd Raymond Poincaré, 92380 Garches, France

**Keywords:** Molecular biology, Inflammation

## Abstract

Sepsis involves endothelial cell (EC) dysfunction, which contributes to multiple organ failure. To improve therapeutic prospects, elucidating molecular mechanisms of vascular dysfunction is of the essence. ATP-citrate lyase (ACLY) directs glucose metabolic fluxes to de novo lipogenesis by generating acetyl-Co-enzyme A (acetyl-CoA), which facilitates transcriptional priming via protein acetylation. It is well illustrated that ACLY participates in promoting cancer metastasis and fatty liver diseases. Its biological functions in ECs during sepsis remain unclear. We found that plasma levels of ACLY were increased in septic patients and were positively correlated with interleukin (IL)-6, soluble E-selectin (sE-selectin), soluble vascular cell adhesion molecule 1 (sVCAM-1), and lactate levels. ACLY inhibition significantly ameliorated lipopolysaccharide challenge-induced EC proinflammatory response in vitro and organ injury in vivo. The metabolomic analysis revealed that ACLY blockade fostered ECs a quiescent status by reducing the levels of glycolytic and lipogenic metabolites. Mechanistically, ACLY promoted forkhead box O1 (FoxO1) and histone H3 acetylation, thereby increasing the transcription of c-Myc (MYC) to facilitate the expression of proinflammatory and gluco-lipogenic genes. Our findings revealed that ACLY promoted EC gluco-lipogenic metabolism and proinflammatory response through acetylation-mediated MYC transcription, suggesting ACLY as the potential therapeutic target for treating sepsis-associated EC dysfunction and organ injury.

## Introduction

Sepsis is a life-threatening condition characterized by infection-induced dysregulated immune responses and multiple organ dysfunction syndrome (MODS). Despite the significant progress in delineating the underlying mechanisms of sepsis pathogenesis, effective treatments or specific diagnostic biomarkers are not clinically available [1]. The pathogenesis of sepsis-associated MODS involves dysregulated immune responses, immune cell infiltration, and vascular dysfunction [2]. Among others, the onset of sepsis drives the proinflammatory activation of vascular ECs to express adhesion molecules to facilitate leukocyte infiltration into underlying tissues, causing organ injury by releasing proteases and oxygen-derived radicals [3]. Thus, EC activation as the hallmark in sepsis-related MODS represents an important therapeutic target.

As previously reported, EC metabolism is markedly perturbed in pathologies such as cancer and diabetes. Metabolic pathways such as glycolysis, fatty acid oxidation, and glutamine metabolism have distinct and essential roles in regulating EC functions such as angiogenesis [4]. Aerobic glycolysis is well known to contribute to the pathogenesis of sepsis and sepsis-related EC activation [5–7]. Except for promoting lactate production, glycolysis-derived pyruvate enters the tricarboxylic acid (TCA) cycle to form acetyl coenzyme A (acetyl-CoA), which is a precursor for glucose-dependent de novo lipogenesis (DNL). ATP-citrate lyase (ACLY) is the key enzyme that converts citrate into acetyl-CoA to promote DNL [8, 9]. Alterations in the expression or activation of ACLY have been observed in various metabolic and pathological conditions, such as cancer and fatty liver diseases [10, 11]. In infectious diseases, toll-like receptor signaling-related macrophage polarization has been demonstrated to be mediated by ACLY-mediated histone acetylation [12]. However, the regulatory role of ACLY in vascular dysfunction and organ injuries in sepsis remains unknown.

In this study, we for the first time demonstrated the regulatory mechanism of ACLY-mediated EC dysfunction in sepsis. We observed the positive correlations of plasma ACLY with markers of EC activation in septic patients. In ECs, overexpression of ACLY promotes, while depletion of ACLY suppresses, the proinflammatory activation and gluco-lipogenic metabolism. Mechanistically, ACLY activation promoted FoxO1 acetylation-dependent nuclear export, as well as histone H3 acetylation, thereby activating MYC transcription to facilitate the expression of proinflammatory and gluco-lipogenic genes in ECs. Furthermore, both mTORC1 and mTORC2-mediated Akt pathway participated in phosphotylation-dependent activation of ACLY in ECs. Taken together, these findings provide a framework to understand the role of ACLY in regulating the proinflammatory activation and gluco-lipogenesis in ECs, highlighting the potential of ACLY as a novel therapeutic target for treating sepsis-associated EC dysfunction and organ injuries.

## Results

### Plasma ACLY was associated with severity of inflammation in sepsis

**Fig. 1:**
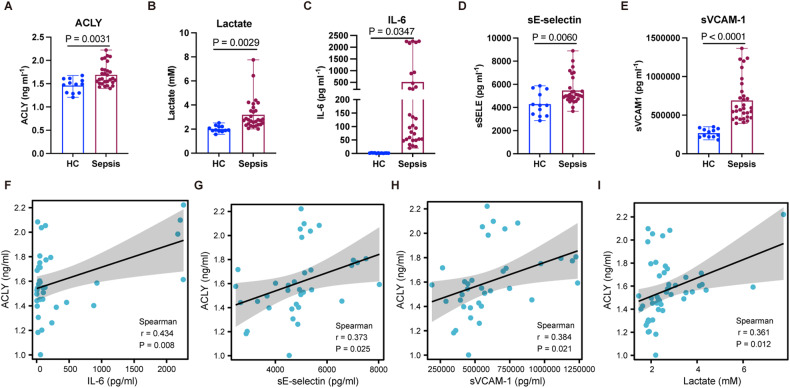
Plasma ACLY levels were associated with the severity of systemic and vascular inflammation in sepsis. **A–E** Levels of ACLY, lactate, IL-6, sE-selectin, and sVCAM-1 were measured in healthy controls (*n* = 12) and patients with sepsis (*n* = 37). **F–I** Correlations of plasma levels of ACLY with IL-6, sE-selectin, sVCAM-1, and lactate were analyzed. Student *t* test was used for A-E. Simple linear regression and Spearman correlation coefficients were used for F-I.

The characteristics of septic patients and healthy controls were shown in Supplementary Table S2. The age and BMI were not significantly different between septic patients and healthy controls (*P* = 0.0725, 0.0929, respectively), indicating the comparability of these two groups. According to the clinical data of septic patients, the SOFA score was 12.5 (9.5, 15.5). The range of the procalcitonin (PCT) level in the 37 patients were from 1.2 to over 100. Additionally, 11 patients were diagnosed to have septic shock. These data indicate that these patients were different in the severity of sepsis. We observed that plasma levels of ACLY and lactate were significantly increased in patients with sepsis compared to those in healthy controls (*P* = 0.0031 and 0.0029, respectively) (Fig. 1A, B). Likewise, the levels of IL-6 (*P* = 0.0347), sE-selectin (*P* = 0.0060), and sVCAM-1 (*P* < 0.0001) were significantly higher in patients with sepsis than in healthy controls (Fig. 1C–E). The correlation analyses showed that plasma levels of ACLY were positively associated with the levels of IL-6, sE-selectin, and sVCAM-1 (*P* = 0.008, 0.025, and 0.021, respectively) (Fig. 1F–H). The plasma level of ACLY was also positively correlated with lactate level in septic patients (*P* = 0.012) (Fig. 1I). Collectively, these results revealed that ACLY levels were closely associated with the severity of patients with sepsis, implying the potentially detrimental effects of intrinsic ACLY on cellular functions.

### ACLY blockade ameliorated endotoxemia-induced inflammation and tissue injury in vivo

**Fig. 2:**
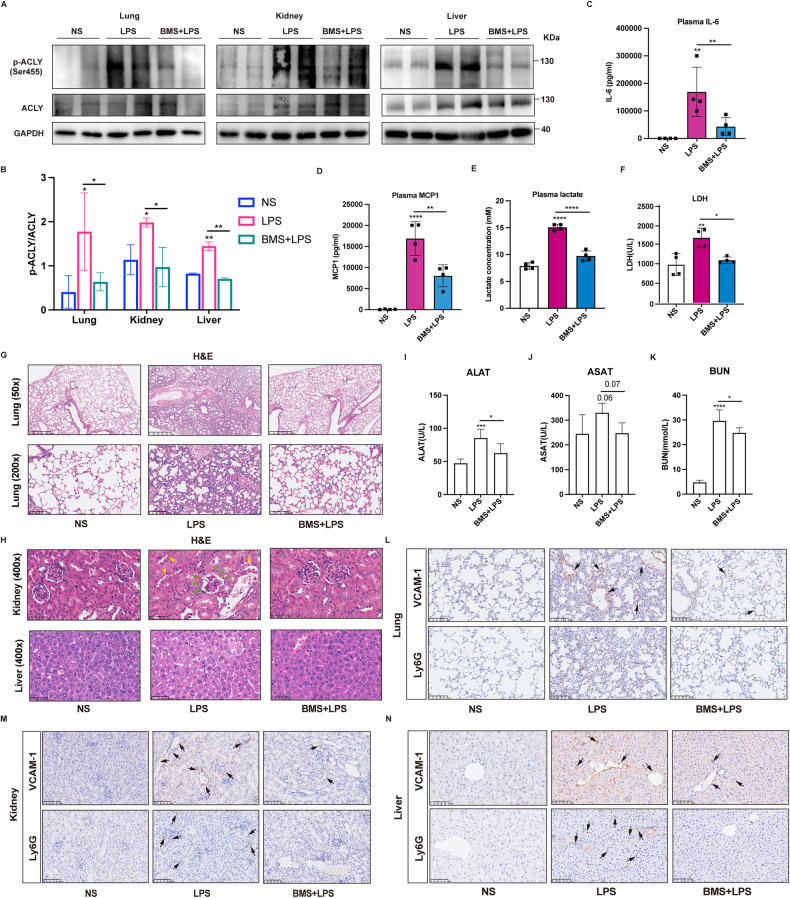
ACLY inhibition alleviated endotoxemia-induced inflammation and organ injury in vivo. Mice were pretreated with BMS-303141 (BMS, 50 mg/kg) prior to LPS injection (*n* = 4). **A, B** Immunoblot showing the expression levels of phosphorylated and total ACLY in the lung, kidney, and liver tissues harvested 16 h after LPS challenge. **C, D** Levels of IL-6 and MCP-1 in plasma harvested 16 h after LPS challenge were measured using ELISA. **(E, F)** Lactate and LDH levels in the plasma 16 h after LPS challenge were measured. **G, H** H&E staining showing the injury in the lung, kidney, and liver tissues 16 h after LPS challenge. Green arrows indicate the shrinking of glomerulus, and yellow arrows indicate the loss of brush border of renal tubules. Magnification is 50 × and 200 × for the lung tissue, and 400 × for the kidney and liver tissues. **I–K** The levels of ALAT, ASAT, and BUN in the plasma samples were detected (*n* = 4). **L–N** IHC staining showing the levels and distribution of VCAM-1 and the infiltration of Ly6G+ neutrophils in the lung, kidney, and liver tissues. NS: normal saline.

The phosphorylated ACLY at serine 455 has been reported to be the activated form of ACLY in adipocyte lipogenesis and macrophage proinflammatory response [13, 14]. To verify the regulatory role of ACLY in sepsis-induced inflammation and organ injury in vivo, mice were injected with ACLY-specific inhibitor BMS-303141 before being subjected to LPS-induced endotoxemia. The hematoxylin and eosin (H&E) staining showed that BMS-303141 administration had no influence on the lung tissues in control mice (Supplementary Fig. 1A). The results showed that levels of phosphorylated ACLY were increased in the lung, kidney, and liver from septic mice, which were inhibited by BMS-303141 administration (Fig. 2A, B). ACLY inhibition significantly decreased the plasma levels of cytokines IL-6 and MCP-1 (Fig. 2C, D). The lactate and LDH levels in plasma were also significantly reduced upon ACLY inhibition (Fig. 2E, F). The H&E staining showed that BMS-303141 alleviated LPS-induced tissue injury in the lung, kidney, and liver, as shown by less immune cell infiltration and tissue necrosis in the lungs, the relieved shrinking glomerulus and recovery of the loss of brush border of renal tubules, as well as the reduced ballooning degeneration of liver cells (Fig. 2G, H). Additionally, LPS challenge increased the levels of ALAT and ASAT related to liver injury as well as the levels of BUN associated with renal injury, which were reduced upon BMS-303141 administration (Fig. 2I–K). Moreover, the vascular proinflammatory responses in the microvasculature were inhibited upon ACLY blockade as indicated by the reduced intensity of VCAM-1 in the lung, kidney, and liver (Fig. 2H–L). The infiltration levels of Ly6G+ neutrophils in tissues were decreased (Fig. 2H–L). These results indicated that ACLY participates in promoting inflammatory response and multiple organ injury in sepsis.

### ACLY activation involved in EC proinflammatory activation induced by LPS

**Fig. 3:**
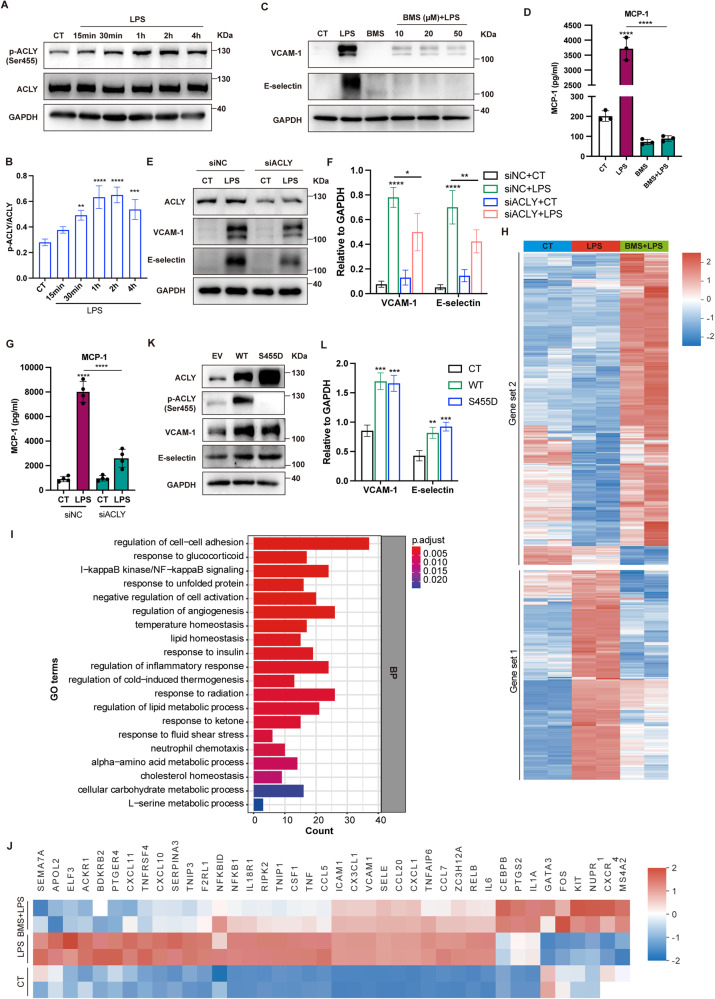
ACLY regulated EC proinflammatory activation in response to LPS. **A, B** Time-course western blot analysis showing the phospho-specific and total ACLY in HUVEC incubated with LPS (1 µg/mL). **C, D** Immunoblotting and ELISA indicating the expression levels of adhesion molecules and the levels of MCP-1 in the presence of ACLY inhibitor BMS-303141 (BMS, 10 µM) in HUVEC stimulated with LPS for 4 h (*n* = 3). **E–G** HUVEC were transfected with siRNA specific for ACLY before 4 h LPS incubation and analyzed for the expression of adhesion molecules using Western blot and the production of MCP-1 using ELISA (*n* = 4). **H** Transcriptomic analysis of HUVEC stimulated with 1 µg/mL LPS ± BMS-303141 for 4 h (*n* = 2). Expression data were log transformed and scaled. **I** GO analysis of DEGs between the LPS and BMS + LPS groups. **J** Heatmap of inflammatory response-associated genes. **K, L** HUVEC were transduced with adenovirus containing ACLY-WT (WT) or ACLY-S455D (S455D) constructs and analyzed for the expression levels of VCAM-1 and E-selectin using Western blot analysis.

To investigate the changes of ACLY in vascular ECs under septic conditons, HUVEC were stimulated with LPS. We observed a time-dependent increase in ACLY phosphorylation, while the total expression of ACLY remained unaltered within 4 h of LPS stimuli (Fig. 3A, B). To examine the role of ACLY activation in EC inflammation, HUVEC were pretreated with BMS-303141 before LPS stimulus. The upregulation of VCAM-1 and E-selectin as well as MCP-1 was effectively abolished by BMS-303141 without affecting cell viability (Fig. 3C, D, Supplementary Fig. 1B–E). The knockdown of ACLY with siRNA showed similar effects on EC inflammation (Fig. 3E–G, Supplementary Fig. 1F–I). RNA sequencing data showed that ACLY inhibition significantly reversed the expression of genes in response to LPS (gene set 1 and 2) (Fig. 3H). Among others, 262 genes was significantly regulated by both LPS stimuli and ACLY inhibition (Supplementary Fig. 1J, K). GO analysis showed that ACLY-regulated genes were enriched in biological processes associated with inflammation (cell–cell adhesion, IκB kinase/NF-κB signaling, regulation of inflammatory response, and neutrophil chemotaxis) and metabolism (lipid metabolic process, cellular carbohydrate metabolic process, etc.) (Fig. 3I). Specifically, a majority of genes enriched in inflammatory response were downregulated on ACLY inhibition (gene set 1), including VCAM-1, E-selectin, ICAM-1, and IL-6, TNF, CXCL1, IL18R1, and CCL5 (Fig. 3J). To verify the proinflammatory role of ACLY and phosphorylated ACLY at serine 455, which is reported to be the activated form of ACLY [13], HUVEC were transfected with adenovirus carrying ACLY-WT and ACLY-S455D constructs. An increase in ACLY phosphorylation was observed in ACLY-WT-overexpressed HUVEC. Both ACLY-WT and ACLY-S455D overexpression resulted in increased expression of VCAM-1, E-selectin, and MCP-1 (Fig. 3K, L, Supplementary Fig. 1L). These data indicated that ACLY contributes to the proinflammatory activation of ECs in response to LPS.

### ACLY inhibition rewired EC gluco-lipogenic metabolism

**Fig. 4:**
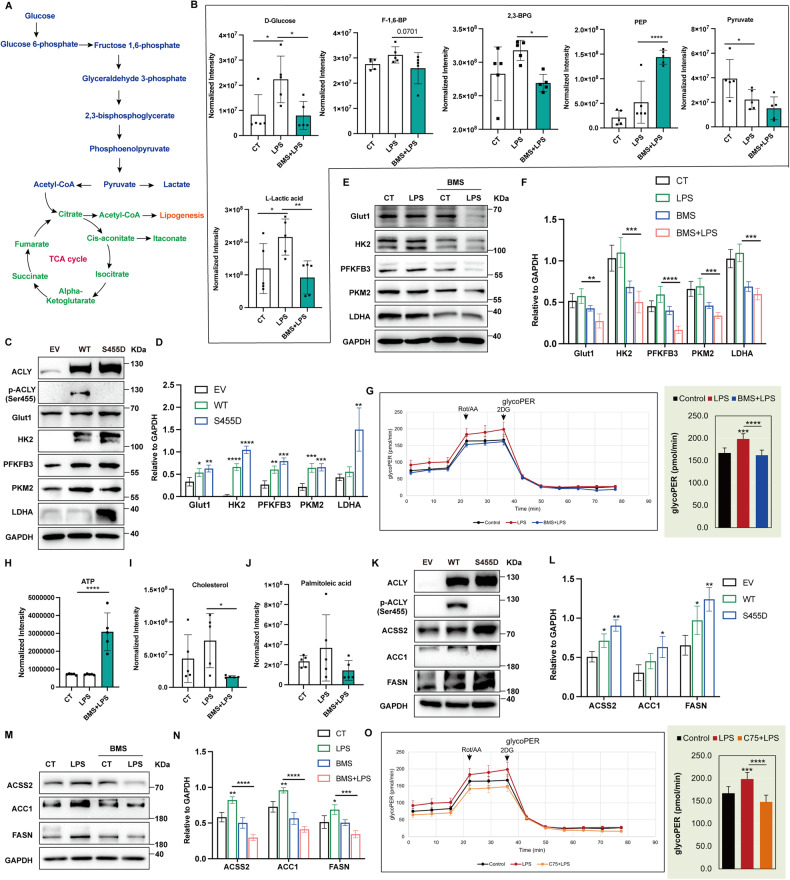
ACLY inhibition rewired EC gluco-lipogenic metabolism in response to LPS. **A** Schematic representation of individual metabolites associated with glycolysis, TCA cycle, and DNL. **B** Metabolomic data of glycolytic metabolites in HUVEC pretreated with BMS-303141 before LPS stimuli (*n* = 5). **C, D** Western blot analysis showing the expression levels of glycolysis-related proteins in ACLY-WT-overexpressing (WT) or ACLY-S455D-overexpressing (S455D) HUVEC. **E, F** HUVEC were pretreated with BMS-303141 before LPS stimulation for 4 h. Expression levels of glycolysis-related proteins were analyzed using immunoblot. **G** HUVEC were pretreated with BMS-303141 before LPS stimuli, followed by the measurement of glycolysis rate (proton efflux rates, PER) for 80 min. Rot&AA: Rotenone & antimycin A; Oligo: Oligomycin. **H** Metabolomic data of ATP production in HUVEC pretreated with BMS-303141 before LPS stimuli (*n* = 5). **I, J** Metabolomic data of metabolites of de novo lipogenesis in HUVEC pretreated with BMS-303141 before LPS stimuli (*n* = 5). **K, L** HUVEC with ACLY-WT (WT) or ACLY-S455D (S455D) overexpression were analyzed for the expression levels of DNL-related proteins using immunoblots. Empty vector was taken as the control. **M, N** HUVEC were pretreated with BMS-303141 before LPS stimulation for 4 h. Expression levels of DNL-related proteins were analyzed using immunoblot. **O** HUVEC were pretreated with C75 before LPS stimuli, followed by the measurement of glycolysis rate (proton efflux rates, PER) for 80 min.

ACLY facilitated DNL by promoting gluco-lipogenic gene expression (Fig. 4A) [15, 16]. As shown in Fig. 4B, ACLY blockade decreased the levels of LPS-induced glycolytic intermediates, including D-glucose, fructose 1,6-bisphosphate (F-1,6-BP), 2,3-bisphosphoglycerate (2,3-BPG), and L-lactic acid. The level of phosphoenolpyruvate (PEP), which has been reported to protect from ischemia/reperfusion-related lung injury [17], was significantly increased by ACLY inhibition (Fig. 4B). Moreover, the expression levels of glycolytic enzymes in HUVEC were significantly increased by ACLY-WT and ACLY-S455D overexpression and reduced by ACLY blockade (Fig. 4C–F). Additionally, ACLY inhibition reduced the level of glycolysis in HUVEC upon LPS stimuli as indicated by less proton efflux rate (Fig. 4G). ACLY directs glucose metabolism to enter TCA cycle and facilitates DNL [13]. We then asked whether the inhibition of ACLY would lead to the adaptation of TCA cycle. The level of ATP were significantly increased by ACLY blockade (Fig. 4H). ACLY inhibition reduced the level of citric acid but increased the level of isocitric acid (Supplementary Fig. 2A, B). Cis-aconitate as a TCA intermediate is converted into itaconic acid which exerts anti-inflammatory function [18]. ACLY inhibition significantly upregulated the level of itaconic acid (Supplementary Fig. 2C, D). Additionally, the level of fumarate, which has been reported to have beneficial effects in sepsis and chronic active multiple sclerosis lesions [19, 20], was also significantly increased by ACLY inhibition (Supplementary Fig. 2E). On the other hand, the levels of 2-keto-glutaramic acid, succinate, and malate were not significantly altered (Supplementary Fig. 2F–H). These results indicated that ACLY blockade may render ECs to an anti-inflammatory status via the inhibition of glycolysis and the adaption of TCA cycle in sepsis.

ACLY is the first key enzyme for glucose-dependent DNL [21, 22]. ACLY blockade showed the trend to reduce the levels of cholesterol and palmitoleic acid in HUVEC increased by LPS (Fig. 4I, J). Furthermore, ACLY-WT and ACLY-S455D overexpression led to increased expression of DNL-related proteins, including ACSS2, ACC1, and FASN, while ACLY blockade inhibited the expression of these proteins in LPS-stimulated HUVEC (Fig. 4K–N). To verify the contribution of fatty acid synthesis per se to EC proinflammatory response and glucose metabolism, C75, an inhibitor of FASN, which is the key enzyme for fatty acid synthesis, was used before LPS stimulus. The upregulation of VCAM-1, E-selectin, and MCP-1 induced by LPS were dramatically abolished by C75 without affecting cell viability (Supplementary Fig. 3A–H). Additionally, the level of glycolysis was reduced upon FASN inhibition as indicated by less proton efflux rate (Fig. 4O). These data revealed that ACLY-mediated de novo fatty acid synthesis plays an important role in regulating EC function during sepsis.

### ACLY activated MYC transcription via acetylation of FoxO1 and histone H3

**Fig. 5:**
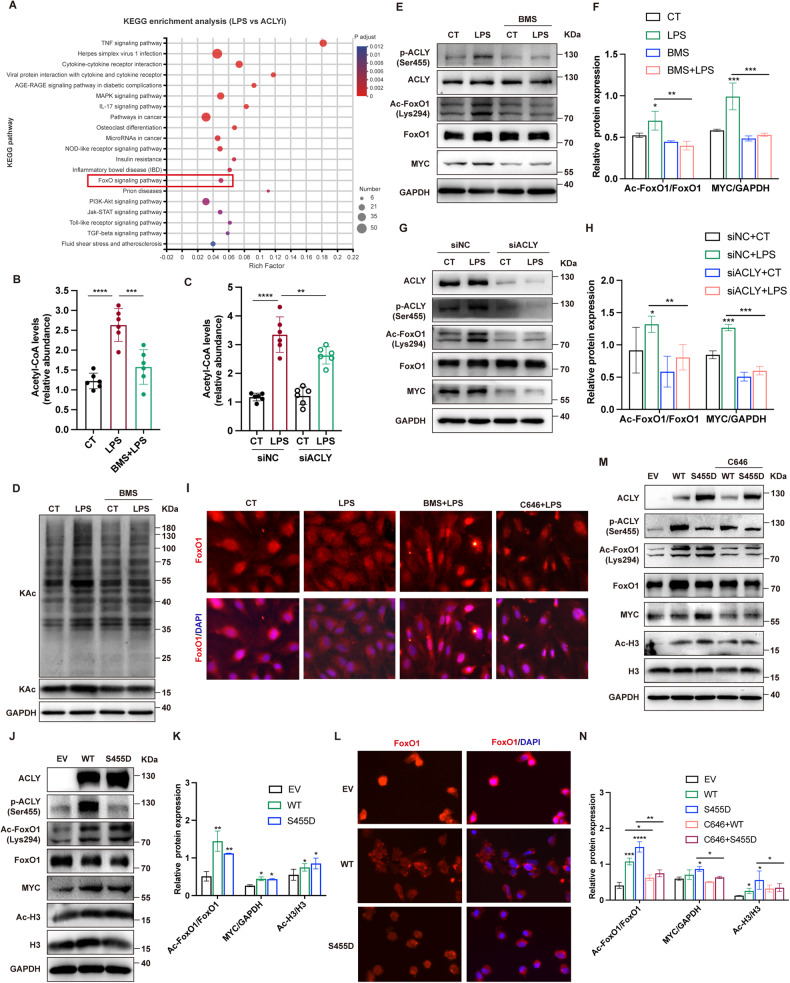
ACLY regulated MYC expression via promoting the acetylation of FoxO1 and H3. **A** KEGG analysis of DEGs from transcriptomic sequencing showing the top 20 enriched pathways involved in ACLY regulation in LPS-induced HUVEC. **B**, **C** Levels of acetyl-CoA in HUVEC treated with BMS-303141 before LPS stimulation were measured (*n* = 6). **D** Western blot analysis showing the global acetylation of lysine in HUVEC treated with or without BMS-303141 before LPS stimulation. (**E**–**H**) HUVEC were pretreated with BMS-303141 or transfected with siRNA specific to ACLY before stimulatedn with LPS for 1 h. Levels of phosphorylated and total ACLY, acetylated and total FoxO1, as well as MYC were tested using Western blot assays. **I** Immunofluorescence staining showing FoxO1 localization in HUVEC stimulated with LPS in the presence of BMS-303141 (10 µM) or C646 (5 µM). **J**, **K** Western blot analysis showing the levels of acetylated FoxO1 and acetylated H3, and the expression of MYC in HUVEC with ACLY-WT (WT) or ACLY-S455D (S455D) overexpression, respectively. **L** Immunofluorescence staining representing FoxO1 nuclear export in ACLY-WT- and ACLY-S455D-overexpressing HUVEC (white arrow). **M**, **N** ACLY-WT-overexpressing (WT) or ACLY-S455D-overexpressing (S455D) HUVEC were treated with p300 inhibitor C646 (5 µM) and the levels of acetylated FoxO1 and acetylated H3, and the expression of MYC were analyzed using immunoblot.

We then explored the regulatory mechanisms of EC functions by ACLY. KEGG analysis of the DEGs demonstrated that FoxO signaling was significantly involved in ACLY inhibition-related regulation of genes in ECs (adjusted *P* = 0.0028) (Fig. 5A). FoxO1 is the most abundant transcription factor that regulates EC function and metabolism via depressing the transcription of MYC [23, 24]. The activity of FoxO1 is regulated by posttranslational modification [25]. We then explored whether LPS stimulation leads to the modification of FoxO1 in ECs. The results showed that both acetylation and phosphorylation levels of FoxO1 were increased by LPS in a time-dependent manner (Supplementary Fig. 4A, B). Since ACLY generates acetyl-CoA serving as a substrate for protein acetylation, we asked whether ACLY could regulate FoxO1 activity via acetylation. Firstly, the levels of acetyl-CoA in HUVEC were increased by LPS, which were significantly inhibited upon ACLY blockade (Fig. 5B, C). Additionally, ACLY inhibition reduced the levels of global acetylation in response to LPS (Fig. 5D). To investigate the modulation of FoxO1 acetylation by ACLY, HUVEC were treated with BMS-303141 or siRNA specific to ACLY before LPS stimulus. The results showed that ACLY deficiency abolished LPS-induced FoxO1 acetylation as well as the upregulation of MYC (Fig. 5E–H, Supplementary Fig. 4C, D). Immunofluorescent staining showed that LPS led to the nuclear export of FoxO1, whereas ACLY inhibition retained FoxO1 in the nucleus (Fig. 5I**)**. Treatment with C646, which inhibits acetyltransferase p300, also resulted in the nuclear accumulation of FoxO1, confirming the effect of FoxO1 acetylation on its nucleoplasm shuttle (Fig. 5I). Moreover, ACLY-WT and ACLY-S455D overexpression resulted in increased levels of FoxO1 acetylation and nuclear export of FoxO1, and induced the expression of MYC (Fig. 5J–L). Except for FoxO1 acetylation, the level of acetylated histone H3 was also increased in HUVEC with ACLY-WT and ACLY-S455D overexpression (Fig. 5J, K), and ACLY deficiency reduced the level of acetylated H3 (Supplementary Fig. 4E–H). The acetylation of FoxO1 has been reported to enhance its sensitivity for phosphorylation [26]. The result showed that expect for the inhibition of FoxO1 acetylation, ACLY blockade also diminished FoxO1 phosphorylation (Supplementary Fig. 4I–L). To confirm that ACLY-associated regulation of MYC was mediated by acetylation, HUVEC overexpressed with ACLY-WT and ACLY-S455D were co-treated with p300 inhibitor. The results showed that C646 effectively reduced ACLY-WT- and ACLY-S455D-related increase of both acetylated FoxO1 and H3 (Fig. 5J). The expression of MYC induced by ACLY-WT and ACLY-S455D overexpression was also abolished by p300 inhibition (Fig. 5M, N). These data revealed that by ACLY promoted the transcription of MYC through the acetylation of both FoxO1 and histones.

### MYC mediated the proinflammatory response and gluco-lipogenesis in ECs

**Fig. 6:**
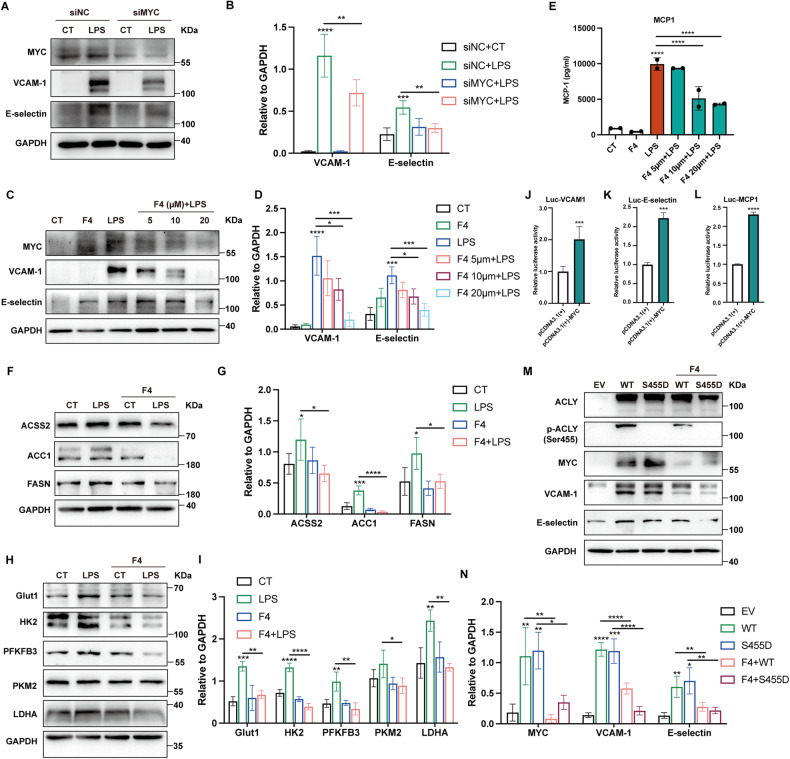
MYC mediated ACLY regulation of EC dysfunction induced by LPS. HUVEC were transfected with siMYC or pretreated with MYC inhibitor 10058-F4 before LPS stimuli for 4 h. **A**–**D** Expression levels of MYC, VCAM-1, and E-selectin were analyzed by immunoblot assays. **E** Levels of MCP-1 production in the supernatants of HUVEC were measured using ELISA (*n* = 2). **F**–**I** HUVEC were pretreated with MYC inhibitor 10058-F4 before LPS stimuli for 4 h. Expression levels of glycolytic and lipogenic enzymes were tested by immunoblot assays. **J**–**L** HEK-293T cells were co-transfected with reconstructed luciferase vectors harboring wild-type promoter region of VCAM-1, E-selectin, and MCP-1 together with the transfection with empty plasmid vector or plasmid vector harboring MYC gene (*n* = 4). **M**, **N** ACLY-WT-overexpressing (WT) or ACLY-S455D-overexpressing (S455D) HUVEC were treated with MYC inhibitor 10058-F4 (10 µM). Expression levels of VCAM-1, E-selectin, and MYC were detected using Western blot assay.

To verify the role of MYC in ECs in response to LPS, MYC was knocked down or inhibited with 10058-F4 prior to LPS stimuli. The results showed that the inhibition of MYC abolished LPS-induced upregulation of VCAM-1, E-selectin, and MCP-1 (Fig. 6A–E). In addition, the expressions of glycolysis- and lipogenesis-related proteins were inhibited upon MYC blockade (Fig. 6F–I). To confirm the transcriptional regulation of VCAM-1, E-selectin, and MCP-1 by MYC, reconstructed luciferase vectors harboring wild-type promoter region of VCAM-1, E-selectin, and MCP-1 were transferred into the human embryonic kidney (HEK)-293T respectively in combination with the transfection with empty plasmid vector or plasmid vector harboring MYC gene. The co-transfection of MYC significantly increased the transcription levels of VCAM-1, E-selectin, and MCP-1 (Fig. 6J–L). To investigate whether MYC mediated the proinflammatory function of ACLY in ECs, HUVEC overexpressed with ACLY-WT- and ACLY-S455D were co-treated with MYC inhibitor. The upregulations of VCAM-1 and E-selectin induced by the ACLY-WT and ACLY-S455D overexpression were diminished upon MYC inhibition (Fig. 6M, N). These data revealed that MYC played an important role in ACLY-mediated proinflammatory response and gluco-lipogenic changes in ECs.

### Both mTORC1 and mTORC2 promoted phosphorylation of ACLY in ECs

**Fig. 7:**
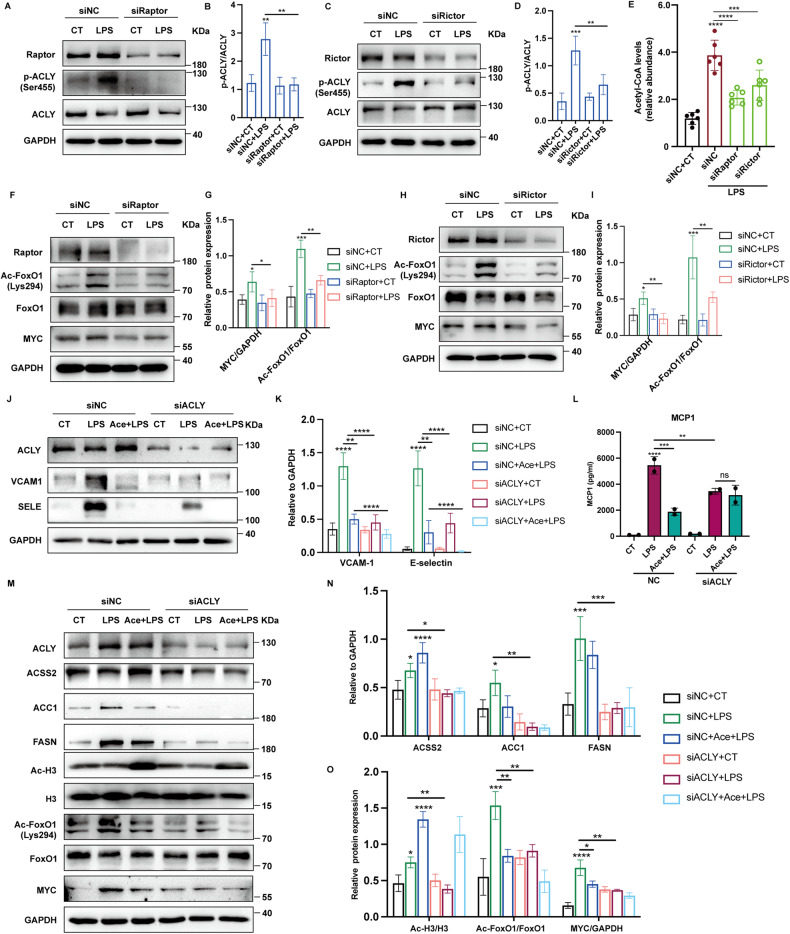
Both mTORC1 and mTORC2 promoted ACLY activation. **A**–**B** HUVEC transfected with siRaptor and siRictor before LPS stimulus were analyzed for phospho-specific and total p70s6k, and phospho-specific and total ACLY using Western blot. **E** Abundance of acetyl-CoA in HUVEC with siRaptor and siRictor before LPS stimuli were analyzed (*n* = 6). **F**–**I** Levels of FoxO1 acetylation and MYC expression in HUVEC transfected with siRaptor and siRictor before LPS stimuli were determined using Western blot assays. **J**, **K** Immunoblot showing the protein levels of VCAM-1 and E-selectin in HUVEC transfected with siACLY before LPS stimuli with or without the supplementation of sodium acetate (Ace, 10 mM). **L** HUVEC were transfected with siACLY and stimulated with LPS in the presence or absence of sodium acetate (Ace, 10 mM). Levels of MCP-1 in the supernatants were analyzed using ELISA (*n* = 2). **M**–**O** HUVEC were transfected with siACLY and stimulated with LPS in the presence or absence of acetate acid (Ace, 10 mM). Protein levels of ACSS2, ACC1, FASN, acetylated FoxO1 and H3, and MYC were analyzed using Western blot.

ACLY is a downstream effector of the mTOR/protein kinase B (Akt) signaling [13, 27]. mTOR kinase is a master regulator of intracellular metabolism and participates in cellular inflammatory responses. To verify the responsibility of mTORC1 and mTORC2 for ACLY activation in ECs, Raptor and Rictor were silenced, respectively before LPS stimuli. Both Raptor and Rictor deletion effectively inhibited LPS-induced phosphorylation of ACLY as well as the cellular levels of acetyl-CoA, respectively (Fig. 7A–E). Additionally, acetylation levels of FoxO1 and the expression of MYC were obviously reduced after the deletion of Raptor and Rictor (Fig. 7F–I). These data indicated that ACLY mediated the regulation of FoxO1-MYC axis by mTOR signaling in ECs.

In the absence of ACLY, ACSS2 can be upregulated to generate acetyl-CoA for DNL and protein acetylation by utilizing exogenous acetate. Therefore, we investigated whether the anti-inflammatory effects of ACLY deficiency could be reversed by the supplementation of exogenous acetate. Intriguingly, the presence of exogenous sodium acetate per se diminished LPS-induced upregulation of VCAM-1, E-selectin, and MCP-1, which was in agreement with the previously reported anti-inflammatory effects of acetate as a short-chain fatty acid (SCFA) [28, 29]. The downregulation of VCAM-1, E-selectin, and MCP-1 upon ACLY inhibition and mTOR signaling blockade was even enhanced by exogenous acetate (Fig. 7J–L, Supplementary Fig. 5A–I). We then examined the effect of acetate on ECs with ACLY-deficiency. ACSS2 was upregulated by acetate (Fig. 7M, N). Additonally, except for the levels of acetylated H3, exogenous acetate failed to reverse the downregulation of either DNL-related protein ACSS2, ACC1, and FASN or acetylated FoxO1 and MYC expression (Fig. 7M, O). These data revealed that acetate metabolism is not involved in the compensation for the effects of ACLY deficiency on DNL and inflammation in ECs.

## Discussion

**Fig. 8:**
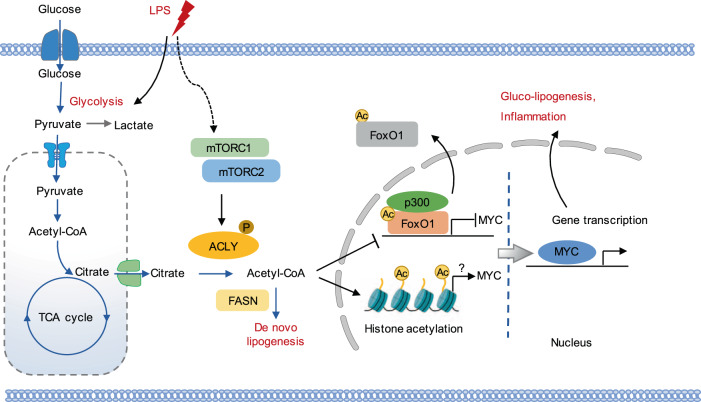
Mechanistic diagram. ACLY was activated by mTORC1/2 signaling, leading to the conversion of citrate into acetyl-CoA. Acetyl-CoA promoted the acetylation of FoxO1 and histone H3 in the nucleus via p300, facilitating the nuclear export of acetylated Foxo1 as well as the transcription derepression of MYC. MYC as a transcription factor activated the expression of proinflammatory and gluco-lipogenic genes in ECs.

Sepsis is characterized by profound metabolic derangements [30]. Gluco-lipogenic metabolism adapts to facilitate transcriptional priming to foster protein acetylation in the context of inflammatory diseases. As the central enzyme in gluco-lipogenic metabolism, ACLY rewires systemic metabolic homeostasis to modulate the transcriptional responses and cellular functions. However, its regulatory role in EC dysfunction during sepsis is still unknown. In this study, we revealed that ACLY activated the transcription of MYC through FoxO1 acetylation-mediated nuclear export, thereby inducing EC proinflammatory activation and gluco-lipogenic metabolism, providing potential therapeutic strategies for treating sepsis-associated EC dysfunction and organ injury (Fig. 8).

ACLY is a strategic enzyme at the crossroads of glucose and lipid metabolism. ACLY has been reported to promote glucose uptake in adipocytes during lipogenesis [13]. Conversely, accelerated glycolytic flux leads to an intensified production of mitochondrial citrate as the substrate of ACLY. It has been reported that glucose-derived metabolic flux increases cytoplasmic acetyl-CoA levels in the regulation of aging and lifespan through ACLY [31]. Additionally, glucose promotes the expression of ACLY in inflammatory T cells in a GLUT3-dependent manner [32]. In the present study, we found that the glycolytic flux in ECs was decreased upon ACLY inhibition. Additionally, the blood glucose levels in septic patients were 12.1 ± 1.9 mmol/L, indicating the hyperglycemia as the complication of sepsis. Thus, we speculate that sepsis-induced hyperglycemia may play a role in increasing the levels of ACLY. Moreover, in our study, the cholesterol levels in septic patients were as normal as 2.4 ± 0.3 mmol/L. Low cholesterol concentrations are a well-recognized manifestation of sepsis and septic shock. The magnitude of hypocholesterolemia relates to the disease severity and outcome and is an early prognostic marker of sepsis [33]. Cholesterol metabolism is modulated by several other enzymes besides ACLY [34]. Therefore, the high level of ACLY is not necessarily to be associated with the level of cholesterol in the blood. It is of interest to elucidate the feedback modulation of cell metabolism by cholesterol in the future.

Increasing studies have revealed the role of ACLY in tumors and cardiovascular and fatty liver diseases [10, 16, 22, 35]. Here, we demonstrated that ACLY facilitated EC gluco-lipogenic metabolism by promoting MYC expression. This study recapitulated the regulatory role of ACLY in glycolysis and DNL. Moreover, we found the protective effects of ACLY inhibitors on vital organ injuries in mice with sepsis, which is supported by recent studies that proposed ACLY inhibition as a potential treatment target for cancer, sepsis, and/or other metabolic diseases [10, 15, 21, 35]. The metabolic activity of ACLY is known to be modulated by its protein expression and phosphorylation [13]. In our study, LPS-challenged mice showed significantly increased phosphorylation of ACLY at serine 455, which is in consistent with previous studies demonstrating that LPS-mediated activation of ACLY is essential for the inflammatory response of macrophage [14, 36]. Moreover, acetylation-related ACLY nuclear translocation could promote the acetylation of NF-*κ*B as well as its activity, leading to the proinflammatory response in macrophages, indicating the possible role of ACLY acetylation in regulating its enzymatic activity for producing acetyl-CoA [37]. Additionally, ACLY has been identified among the O-GlcNAcylated proteins in septic mice. In NButGT-treated septic mice which showed globally increased protein O-GlcNAcylation, ACLY O-GlcNAcylation level was decreased, indicating the key role of ACLY O-GlcNAcylation in response to metabolic change and stress [38]. In a pilot clinical study, an increased level of serum ACLY was identified in pediatic patients with sepsis. However, the level of ACLY was lower in non-survivors [39]. It should be noticed that these data were observed in children which cannot be transposed to adult patients and further studies are needed to confirm the diagnostic value of ACLY in sepsis. All these data pinpoint the involvement of ACLY in sepsis and open a new therapeutic avenue for treating inflammation-related diseases.

The metabolic changes at different phases during sepsis are important for understanding septic pathogenesis. In early sepsis, both increased glycolysis and TCA cycle contributed to the inflammatory response in macrophages [12]. Additionally, mitochondrial ATP synthase activity is increased in the early stage of endotoxic shock but decreased in the late stage, indicating both short-term and longer-term adaptations at the level of oxidative phosphorylation [40]. ECs rely on glycolysis for ATP generation during sepsis, whereas the roles of other metabolic pathways in ECs are less studied [41]. In this study, we found that ACLY and FASN blockade inhibited EC inflammation. Moreover, it has been reported that the inhibition of FASN impaired EC angiogenesis via mTOR malonylation in diabetic retinopathy [42]. Also, inhibition of FASN has been reported to alleviate sepsis-associated inflammation via inhibiting the activation of inflammasome [43]. In light of these findings, the lipogenesis-dependent regulation of cellular functions may represent promising therapeutic targets for treating sepsis.

MYC was demonstrated in our study as the key mediator for ACLY-related regulation of EC metabolism and inflammation. MYC is a highly pleitropic transcription factor and is associated with broad effects on cell proliferation, angiogenesis, apoptosis, and differentiation [44]. In addition to its role in various human cancers, dysregulation of MYC is involved in the regulation of metabolic diseases such as diabetes and fatty liver diseases [45, 46]. It has been reported that MYC promoted lipogenesis in several cell types via cooperating with sterol-regulated element-binding protein (SREBP-1) [47]. Additionally, intestine-specific MYC-disrupted mice ameliorated high-fat diet-induced obesity and hepatic steatosis [47]. Moreover, long-chain acyl-CoA synthetase 4 (ACSL4) has been reported to upregulate lipogenic enzymes via MYC, thereby increasing lipogenesis [48]. On the other hand, the transcriptional de-repression of MYC leads to cell quiescence characterized by reduced glycolysis in sprouting angiogenesis [23]. Therefore, the transcriptional activity of MYC might be a putative therapeutic target to counteract the metabolic disorder in diseases including sepsis.

Acetyl-CoA is a central node in carbon metabolism and plays a critical role in regulating protein acetylation and a wide range of biological processes [49]. ACLY regulates acetylation by controlling net acetyl-CoA input, while ACSS2 recycles acetate for acetyl-CoA production when the metabolic resources are limited [50, 51]. In the absence of ACLY, glucose is converted into acetate, and the cells switch from using glucose to endogenous acetate for acetyl-CoA synthesis and lipogenesis [52]. It has been reported that ACLY knockdown causes ACSS2 upregulation to compensate acetyl-CoA synthesis and lipogenesis [53–55]. In contrast, ACLY-deficiency in pancreas as well as adipocyte-specific ablation of ACLY in female mice suppressed ACSS2 expression [56, 57]. However, in our study, ACLY deletion did not lead to increasd expression of ACSS2, suggesting that ACSS2 may not participate in the compensation of ACLY deficiency in ECs. Acetate derived from the deacetylation process can be recycled in the nucleus, leading to nuclear regeneration of histone acetylation [52]. Acetate can also promote histone acetylation via inhibiting histone deacetylase (HDAC) activities [58]. In our study, we found that exogenous acetate increased the level of histone H3 acetylation in ECs, but failed to rescue the downregulation of ACSS2, ACC1, and FASN, suggesting that acetate metabolism is not engaged in the compensatory mechanism for ACLY deficiency-related inhibition of DNL in ECs. Further studies are needed to understand the distinct roles of ACLY and ACSS2 in EC dysfunction during sepsis.

Collectively, we for the first time demonstrated that ACLY activated MYC transcription via acetylation of FoxO1 and H3, thereby leading to enhanced gluco-lipogenic metabolism and proinflammatory response in ECs under septic conditions. These findings revealed the significance of ACLY in modulating EC metabolism and proinflammatory activation in sepsis, implying the great therapeutic potential of ACLY for treating EC dysfunction-associated tissue injuries in sepsis.

## Materials/subjects and Methods

### Patients

Patients diagnosed with sepsis in the Department of Critical Care Medicine in Shanghai Ruijin Hospital from May 1 to December 20, 2022, were enrolled. This study protocol conformed to the ethics guidelines of the Declaration of Helsinki and was approved by the ethics committee of Ruijin Hospital (No. 20210101). Informed consent was obtained from each participant. The enrollment criteria were as follows: (1) patients aged 18–90 years, (2) meeting sepsis 3.0 definition, and (3) hospital stay > 24 h. Exclusion criteria were: (1) discharge or death within 24 h after admission; (2) participation in other clinical research; (3) emergency surgery after admission; (4) malignant tumor; (5) pregnant or lactating patients; and (6) lack of necessary clinical data. Finally, 12 healthy volunteers and 37 patients with sepsis were enrolled.

Peripheral blood was withdrawn from healthy volunteers and patients with sepsis on the day of enrollment. Plasma samples were obtained after centrifugation at 1500 rpm for 10 min and were stored at −80 °C for further analysis.

### Murine model of sepsis

Male C57BL/6 N mice (7–10 weeks, 20–25 g) were obtained from Charles River (Beijing, China) and randomly divided into experimental groups (*n* = 5 per group). No statistical methods were used for the animal sample size. The mice were injected intraperitoneally with lipopolysaccharide (LPS, L2630, E. coli 0111:B4, Sigma Aldrich, MA, USA) (5 mg/kg body weight [59, 60]) in 0.9% saline (0.9% NaCl) to induce endotoxemia. In the intervention group, the mice were intraperitoneally injected with BMS-303141 (S0277, Selleck, Shanghai, China) (50 mg/kg body weight) 1 h before LPS injection. Vehicle control mice were intraperitoneally injected with 100 µL of saline. After 16 h of LPS challenge, the mice were anesthetized and the blood samples were collected. The organs were snap frozen or fixed with formalin for further examination. The frozen organs were stored at −80 °C. Investigators were blinded to the group allocation for the analysis. The protocols for animal experiments were approved by the Animal Ethics Committee of Ruijin Hospital Affiliated to Shanghai Jiaotong University School of Medicine (No. 092) and were in line with the International Guidelines for Care and Use of Laboratory Animals (National Academy of Sciences Health Publication No. 85–23, revised in 1996).

### Cell culture

Primary human umbilical vein endothelial cells (HUVEC) were isolated from human umbilical cords as previously reported [61]. Briefly, fresh human umbilical cords were acquired from the Department of Obstetrics in Ruijin Hospital with the consent of donors. Isolated HUVEC were cultured in endothelial cell medium (1001, ScienCell, CA, USA) with 5% fetal bovine serum, suspended in streptomycin and penicillin, and maintained at 37 °C under a humidified atmosphere with 5% CO_2_. HUVEC at passages 1–4 were used for all experiments. HUVEC were used in accordance with the human subject guidelines of Ruijin Hospital Shanghai Jiao Tong University.

HUVEC were transfected with small-interfering RNAs (siRNAs, GenePharma, Shanghai, China) to analyze the functions of ACLY, MYC, raptor, and rictor during EC activation. Negative control siRNA was used as the control (GenePharma). HiPerFect (301704, Qiagen, Germany) was used as the transfection reagent following the manufacturer’s instructions. For experiments involving ACLY-WT and ACLY-S455D overexpression, the cells at 80–90% confluence were incubated with adenoviruses carrying human wild-type ACLY construct and ACLY-S455D construct, respectively (pADV-EF1-mNeonGreen-CMV vector, OBiO, Shanghai, China). Adenoviruses were then removed, and the cells were incubated in fresh medium for another 42 h. The transfection efficacy was validated using Western blot analysis. The siRNA sequences are shown in Supplementary Table 1.

### RNA extraction and quantitative RT-PCR

Total RNA was extracted from cell and tissue lysates using the TRIzol reagent (R401, Vazyme, Jiangsu, China) following the manufacturer’s protocol. RNA concentration and purity were measured using a Gen5 Microplate Spectrophotometer (BioTek, VT, USA). Reverse transcription (RT) of RNA was carried out using the HiScript III RT SuperMix (R323, Vazyme). ChamQ Universal SYBR qPCR Master Mix (Q711, Vazyme) was used for quantitative polymerase chain reaction to analyze the mRNA levels. The sequences of human primers used are listed in Supplementary Table 1.

### RNA sequencing analysis

Total RNA was extracted from HUVEC stimulated with LPS in the absence and presence of BMS-303141. The concentration and purity of isolated RNA were measured using a ND-800 spectrophotometer (Thermo Fisher Scientific, DE, USA). The RNA libraries were constructed using a Truseq RNA Library Prep Kit. Sequencing was carried out using a 2 × 150 bp PE configuration. The clean data (reads) were mapped using Hisat2 (version 2.1.0). Then, we performed gene expression analysis using RSEM (version 1.3.1). Differential expression analysis among different groups was conducted using DESeq2, and then |FC| > 2 and FDR < 0.05 were determined as thresholds for differentially expressed genes (DEGs). Gene Ontology (GO) analysis was used to determine the significant biological processes of a particular gene set (*q* < 0.05). The Kyoto Encyclopedia of Genes and Genomes (KEGG) analysis was used to identify the most significant signaling pathways involved in BMS-303141-regulated genes (adjusted *P* < 0.05). The Venn analysis was used to calculate the number of genes in different gene sets.

### Metabolomic analysis

For metabolomic analysis, HUVEC were plated and pretreated with or without BMS-303141 0.5 h before stimulation with LPS for 4 h (*n* = 5 per group). The cells were washed and scraped with phosphate-buffered saline. The metabolites were extracted from cell lysates, separated using liquid chromatography–mass spectrometry (Thermo Fisher Scientific), and identified through tandem mass spectrometry analysis (Q Exactive FOCUS, Thermo Fisher Scientific).

### Measurement of lactate levels

The plasma samples from mice and patients were collected for measuring the lactate levels following the manufacturer’s instructions (L-Lactate Assay Kit, 1200011002, Eton Bioscience, CA, USA).

### Western blot assay

The cells and mouse tissues were lysed using RIPA buffer (#89901, Thermo fisher Scientific) supplemented with PhosSTOP and complete protease inhibitors (Merck, Beijing, China). The protein samples were subjected to 12.5% SDS-PAGE and transferred to a polyvinylidene fluoride membrane (162-0177, BioRad, Shanghai, China). Blots were probed with appropriate primary antibodies against VCAM-1 (ab134047, Abcam, Cambridge, UK), E-selectin (20894-1-AP, Proteintech, Shanghai, China; sc-137054, Santa Cruz, California, USA), Phosphorylated ACLY (Ser455, 4331, CST, Danvers, MA), ACLY (13390, CST), Raptor (48648, CST), Rictor (9476, CST), phosphorylated FoxO1 (Ser256, 84192, CST), FoxO1 (2880, CST), acetylated FoxO1 (Lys294, AF2305, Affinity, PA, USA), MYC (9402, CST), FASN (3180, CST), ACSS2 (3658, CST), ACC1 (4190, CST) (MA, USA), and pan-acetyl-lysine (PTM-105RM, PTM Biolabs, Shanghai, China) at 4 °C overnight. The blots were then incubated with the appropriate secondary antibodies (1:5000, proteintech) and detected using a horseradish peroxidase substrate (WBLUF0500, Millipore, USA). For internal controls of equal loading, the blots were also stripped with stripping buffer (100 mmol/l 2-mercaptoethanol, 2% SDS, 62.5 mmol/l, Tris pH 6.8) and reprobed with ACLY, FoxO1, or GAPDH antibodies.

### Immunofluorescent staining

HUVEC were seeded on a Millicell eight-well glass (PEZGS0416, Millipore). After indicatd treatments, the cells were fixed with 1% paraformaldehyde for 20 min and permeabilized with 0.25% Triton X-100 (Sigma–Aldrich) for 5 min on ice. The cells were then blocked with 3% bovine serum albumin (Sigma–Aldrich) and incubated with FoxO1 antibody overnight at 4 °C. After washing, the cells were incubated with goat anti-rabbit secondary antibody conjugated with Alexa Flour 555 (A32794, Thermo Fisher Scientific). The nuclei were stained with 4′,6-diamidino-2-phenylindole (D9542, Sigma–Aldrich), and the slides were mounted with a microscope cover glass (803400130, CITOGLAS, Jiangsu, China). The images were taken using an Olympus microscope (DP73, Japan).

### Histological and Immunohistochemical staining

Paraffin-embedded mouse kidney, lung, and liver tissue sections were Paraffin-embedded mouse kidney, lung, and liver tissue sections were stained with hemotoxylin & eosin (H&E) for blinded histopathologic assessment. For immunohistochemical detection of VCAM-1 and Ly6G, the sections were stained with antibodies against VCAM-1 (1:500, ab134047, Abcam) and Ly6G (1:1000, ab238132, Abcam) at 4 °C overnight, followed by incubation with biotinylated secondary antibody for 30 min at 37 °C, and finally visualized with a 3,3'-diaminobenzidine solution and counterstained with hematoxylin. The images were taken using a light microscope (BX50, Olympus).

### Enzyme-linked immunosorbent assay

The concentrations of monocyte chemoattractant protein (MCP)-1, IL-6, soluble VCAM-1 (sVCAM-1), and sE-selectin in patient plasma, supernatants of cultured HUVEC, and mouse plasma were measured using enzyme-linked immunosorbent assay (ELISA) kits (MultiSciences Biotechnology, Hangzhou, China) following the manufacturer’s instructions. The concentration of ACLY in plasma samples from patients was measured using ELISA kits (P53396, RayBiotech, GA, USA) following the manufacturer’s instructions.

### Measurement of acetyl-CoA levels

After indicated experiments, HUVEC were harvested, the cell pellets were suspended in assay buffer, and the cell suspensions were homogenized on ice. After centrifugation, the supernatants were deproteinized. Deproteinized and standard samples were used to measure the levels of acetyl-CoA (ab87546, PicoProbe Acetyl-CoA Assay Kit, Fluorometric, Abcam) following the manufacturer’s instructions.

### Statistics

All data were expressed as mean ± standard deviation (SD) of at least three independent experiments. Statistical significance was determined using the two-tailed unpaired-sample *t* test between two groups or the one-way analysis of variance (ANOVA) followed with Bonferroni multiple comparison test. *P < 0.05, **P < 0.01, **P < 0.001, ****P < 0.0001. The images were prepared using GraphPad Prism version 9.0 (GraphPad Prism Software, San Diego, USA).

## Supplementary information

Supplementary information

Figure S1

Figure S2

Figure S3

Figure S4

Figure S5

Original data

aj-checklist

## Data Availability

Transcriptome profiling raw data generated with this study have been deposited in Gene Expression Omnibus (GEO) database under accession code GSE216178. The metabolomic data generated with this study is available at www.ebi.ac.uk/metabolights/MTBLS6161. The datasets used and/or analyzed during the current study are available from the corresponding author upon reasonable request.
