# Supplementary material for: ATP-citrate lyase controls endothelial gluco-lipogenic metabolism and vascular inflammation in sepsis-associated organ injury
Source: Cell Death Dis. 2023 Jul 6;14(7):401. doi: 10.1038/s41419-023-05932-8 (PMC10325983; doi:10.1038/s41419-023-05932-8)
Supplement: Supplementary file 1 — Supplementary information [file 41419_2023_5932_MOESM1_ESM.docx]

**Supplementary Material**

**Supplementary Table S1. Sequences for siRNA and primers**

| **siRNA** | **sense 5‘-3’** | **antisense 5‘-3’** |
| --- | --- | --- |
| siACLY | GGCAUGUCCAACGAGCUCAA | UUGAGCUCGUUGGACAUGCC |
| siMYC | GAGGAUAUCUGGAAGAAAUTT | AUUUCUUCCAGAUAUCCUCTT |
| siRaptor#1 | GCGUCACACUGGAUUUGAUTT | AUCAAAUCCAGUGUGACGCTT |
| siRaptor#2 | CCGAGAAUGAGGAGCAUAUTT | AUAUGCUCCUCAUUCUCGGTT |
| siRaptor#3 | CGAGAUUGGACGACCAAAUTT | AUUUGGUCGUCCAAUCUCGTT |
| siRictor#1 | GCGAGCUGAUGUAGAAUUATT | UAAUUCUACAUCAGCUCGCTT |
| siRictor#2 | CCAAGACAGUUGGAGGCUUTT | AAGCCUCCAACUGUCUUGGTT |
| siRictor#3 | GCCUUAAGUUAUGCAUCAUTT | AUGAUGCAUAACUUAAGGCTT |
| **Gene name** |  |  |
| VCAM1 | ACCACATCTACGCTGACAATGAATCC | AACACTTGACTGTGATCGGCTTCC |
| SELE | GAAGAGGTTCCTTCCTGCCAAGTG | CAGAGCCATTGAGCGTCCATCC |
| MCP1 | CCCCAGTCACCTGCTGTTAT | CAGATCTCCTTGGCCACAAT |
| MYC | CGACGAGACCTTCATCAAAAAC | CTTCTCTGAGACGAGCTTGG |
| ACLY | CAGAATCGGTTCAAGTATGCTC | AAGTTTTCCACGACGTTTGATC |
| Raptor | GACACGGAAGATGTTCGACAAG | ATCTGAGAAGCAACGCTCTC |
| Rictor | CGAGCCAGTGCAGCCTTGAAC | ACTGATCCCGTTTCTGGTGTGTTG |
| GAPDH | GGTGAAGGTCG GAGTCAACG | CAAAGTTGTCATGGATGGACC |

| **Characteristics** | **Healthy Controls** | **Sepsis** |
| --- | --- | --- |
| **Numbers** | 12 | 37 |
| **Age (year)** | 59±11 | 65±13 |
| **Gender, male/female** | 7/5 | 21/16 |
| **BMI (kg/cm^2^)** | 21±0.7 | 23±1.3 |
| **Sites of Infection, N (%)** |  |  |
| Lung | - | 8 (21.6) |
| Abdominal | - | 14 (37.8) |
| Blood | - | 4 (10.8) |
| Others | - | 11 (29.7) |
| **SOFA Score** | - | 12.5 (9.5,15.5) |
| **Laboratory Tests** |  |  |
| CRP (mg/L) | - | 98.6±17.3 |
| PCT (ng/mL) | - | 6.9 (1.6, 69.8) |
| WBC (10^9^/L) | - | 8.9±1.1 |
| PLT (10^9^/L) | - | 111.3 ± 23.8 |
| Lactate (mg/L) | - | 4.1±0.8 |
| Blood glucose (mmol/L) | - | 12.1±1.9 |
| Cholesterol (mmol/L) |  | 2.4±0.3 |
| [**Complication**](javascript:;)**s，N (%)** | - |  |
| Acute respiratory failure | - | 17 (45.9) |
| Acute cardiac dysfunction | - | 9 (24.3) |
| [Acute](javascript:;) [kidney](javascript:;) [injury](javascript:;) | - | 18 (48.6) |
| Acute hepatic insufficiency |  | 9 (24.3) |
| Septic shock | - | 11 (29.7) |
| **28-day mortality, N (%)** | - | 10 (27.0) |
| **Hospital mortality, N (%)** | - | 7 (18.9) |

**Supplemental Table S2. Characteristics of septic patients in our study.**

**Supplementary Figure legends**

**Supplementary Fig. 1 EC response to ACLY inhibition in the presence of LPS. (A)** Mice were i.p. injected with BMS-303141 (50mg/kg body weight). 16h later, the lung tissues were harvested and fixed for H&E staining. **(B-D)** RT-qPCR data showing the effects of BMS-303141 on LPS-induced mRNA expression of VCAM1, SELE, and MCP1 (n = 4). **(E)** Cell viability of EC incubated with or without BMS-303141 was measured using CCK-8 (n = 6). **(F-I)** HUVEC were transfected with siNC or siACLY before stimulated with LPS, and mRNA expressions of ACLY, VCAM1, SELE, and MCP1 were tested using RT-qPCR. Data are representative of at least two independent experiments. **(J)** Venn analysis of genes sensitive to LPS stimuli and ACLY inhibition. **(K)** Heatmap showing genes both significantly different between CT and LPS groups and between LPS and BMS-303141+LPS groups. **(L)** The levels of MCP1 in the supernatants of ACLY-WT and ACLY-S455D overexpressed HUVEC were measured with ELISA (n = 2 per group).

**Supplementary Fig. 2 The effects of ACLY inhibitor on TCA cycle intermediates in HUVEC. (A-H)** The levels of TCA cycle-related metabolites in HUVEC pretreated with BMS-303141 before LPS stimuli (n = 5).

**Supplementary Fig. 3 FASN inhibition abolished the pro-inflammatory response in HUVEC. (A, B)** HUVECs pretreated with C75 before LPS stimuli. Western blot analysis showed the expression levels of VCAM1, SELE, and FASN. **(C)** ELISA showed the production of MCP1 by HUVECs (n = 3). **(D-G)** The mRNA levels of FASN, VCAM1, SELE, and MCP1 in HUVEC pretreated with C75 (20 µM) before LPS stimuli were analyzed by RT-qPCR (n = 2). **(H)** Cell viability of HUVEC treated with C75 (20 µM) for 4h was analyzed by CCK8 (n = 6).

**Supplementary Fig. 4 ACLY activation regulates FoxO1 acetylation and phosphorylation. (A, B)** Western blot showed the time-dependent increase of acetylated and phosphorylated FoxO1 induced by LPS. **(C, D)** The mRNA levels of MYC in HUVEC pretreated with BMS-303141 or transfected with siACLY were detected by RT-qPCR (n = 2). **(E-H)** Western blot showed the levels of acetylated H3 in HUVECs induced by LPS with or without ACLY deficiency. **(I-L)** HUVEC were pretreated with BMS-303141 or transfected with siACLY before LPS stimuli and analyzed for the protein levels of phosphorylated (Ser256) and total FoxO1 by western blot assays.

**Supplementary Fig. 5 Exogenous acetate failed to rescue ACLY inhibition-related anti-inflammatory effects on HUVEC. (A-C)** HUVEC were pretreated with BMS-303141 before LPS stimuli for 4h in the presence or absence of sodium acetate (Ace, 10 mM). The protein levels of VCAM1, SELE, as well as acetylated and total H3 were detected using immunoblot assays **(A, B)**, and the levels of MCP1 in the supernatants were tested by ELISA (n = 2) **(C)**. **(D-I)** HUVEC were transfected with siRaptor#2 or siRictor#1 before LPS stimuli for 4h in the presence or absence of sodium acetate (Ace, 10 mM). The protein levels of VCAM1, SELE, ACSS2, as well as acetylated and total H3 were detected by western blot. The levels of MCP1 in the supernatants were tested by ELISA (n = 2 per group).
