# Supplementary figures and images for: ATP-citrate lyase controls endothelial gluco-lipogenic metabolism and vascular inflammation in sepsis-associated organ injury
Source: Cell Death Dis. 2023 Jul 6;14(7):401. doi: 10.1038/s41419-023-05932-8 (PMC10325983; doi:10.1038/s41419-023-05932-8)

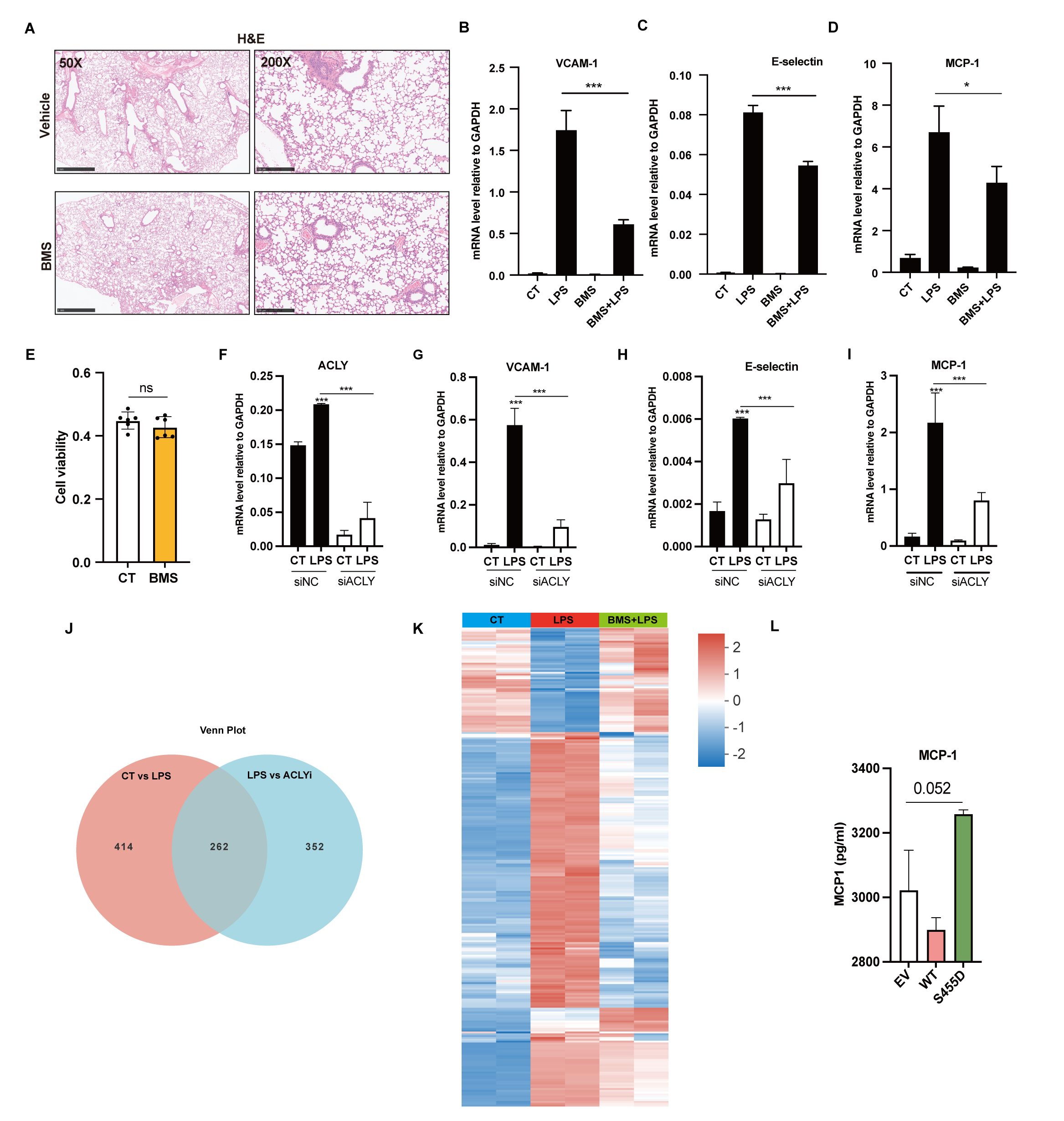

Supplement: Supplementary file 2 — Figure S1 [file 41419_2023_5932_MOESM2_ESM.tif]

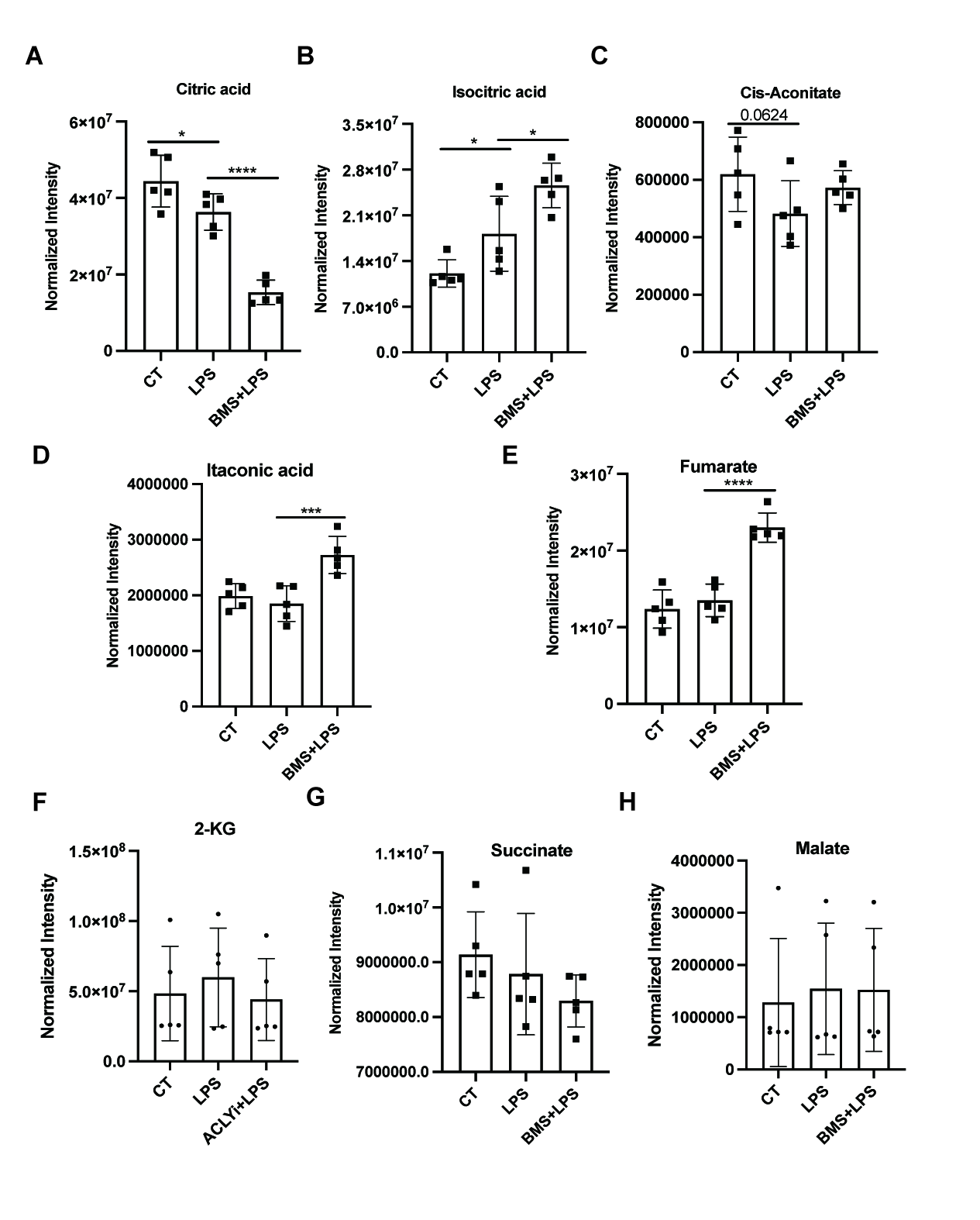

Supplement: Supplementary file 3 — Figure S2 [file 41419_2023_5932_MOESM3_ESM.tif]

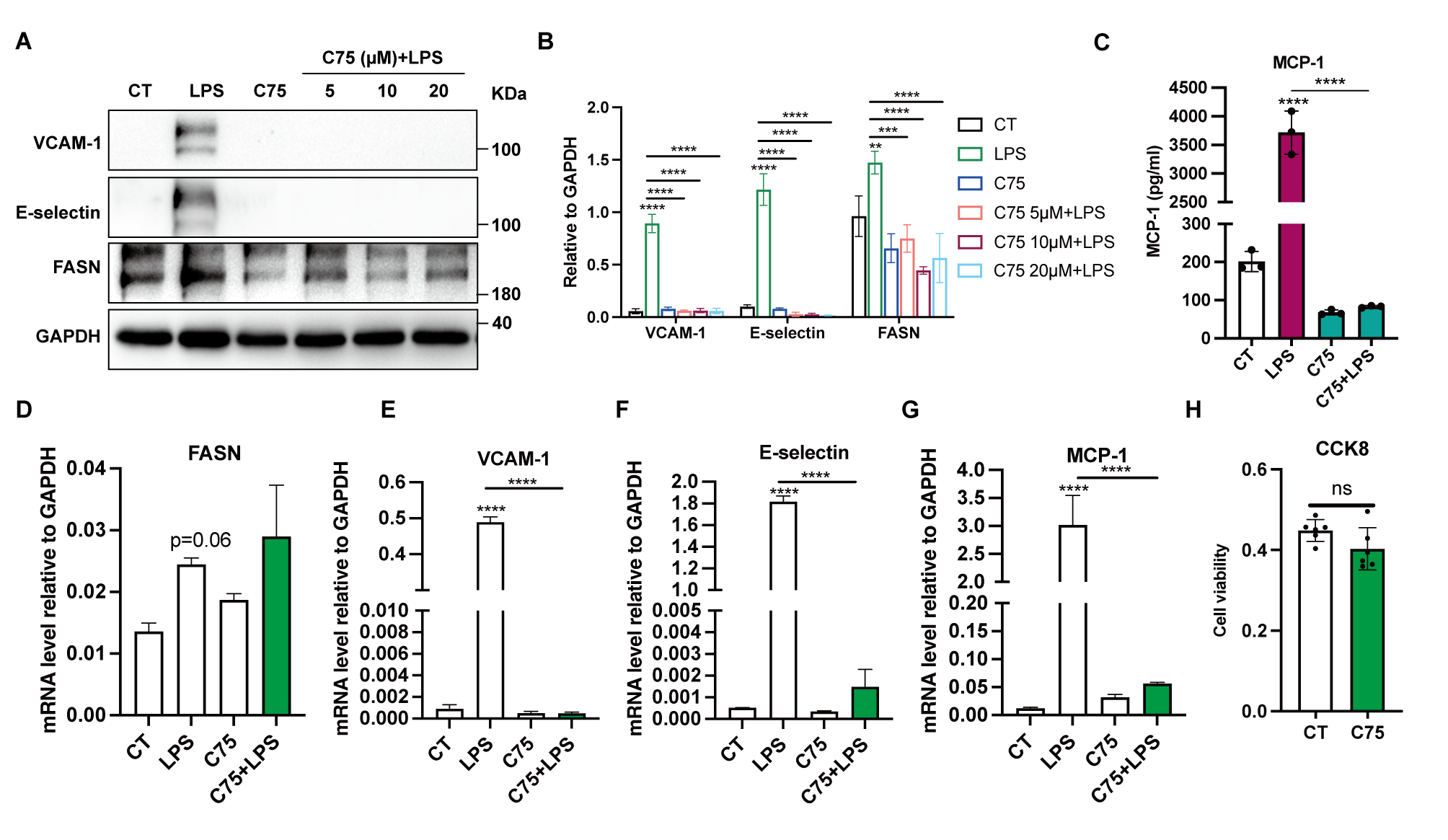

Supplement: Supplementary file 4 — Figure S3 [file 41419_2023_5932_MOESM4_ESM.tif]

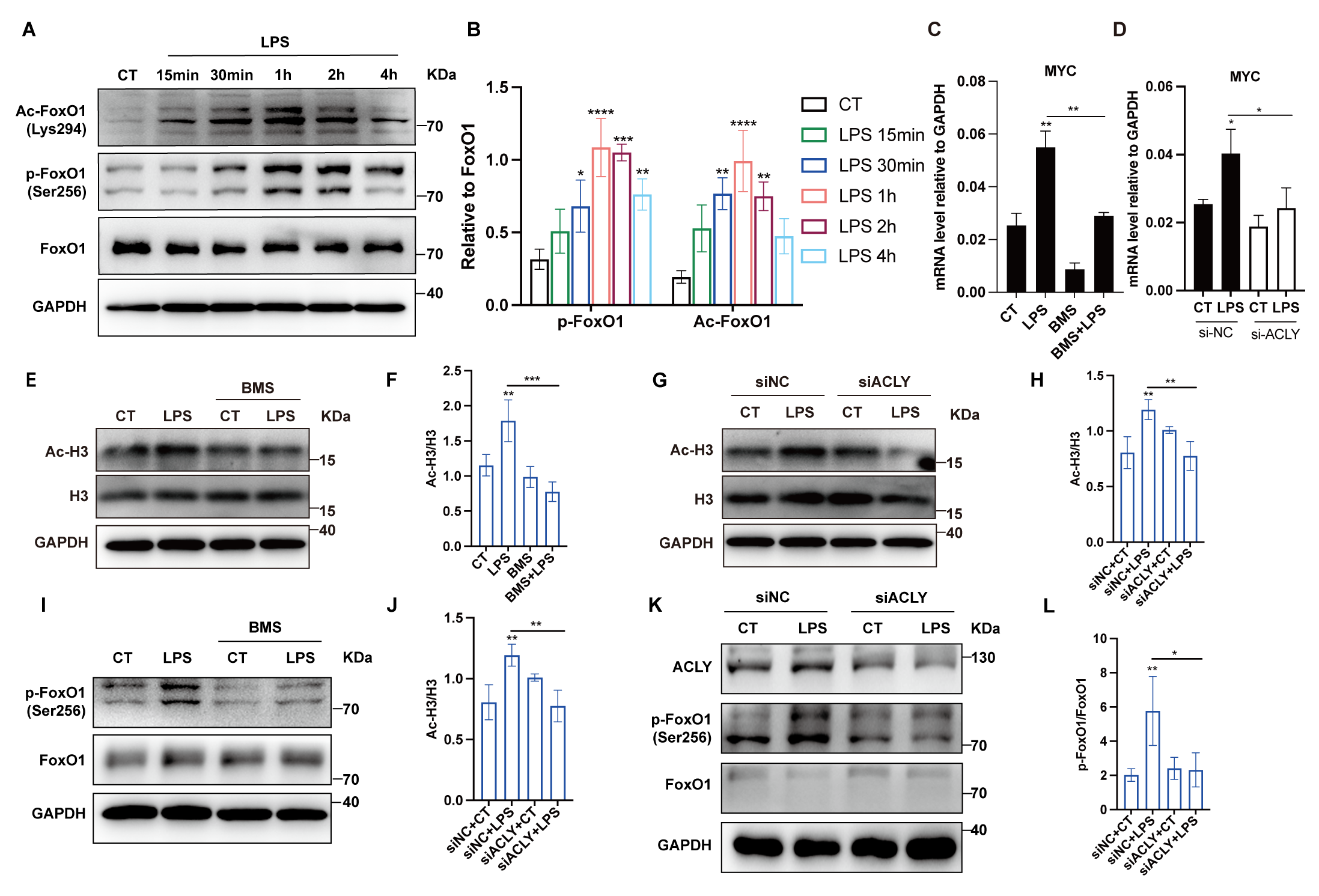

Supplement: Supplementary file 5 — Figure S4 [file 41419_2023_5932_MOESM5_ESM.tif]

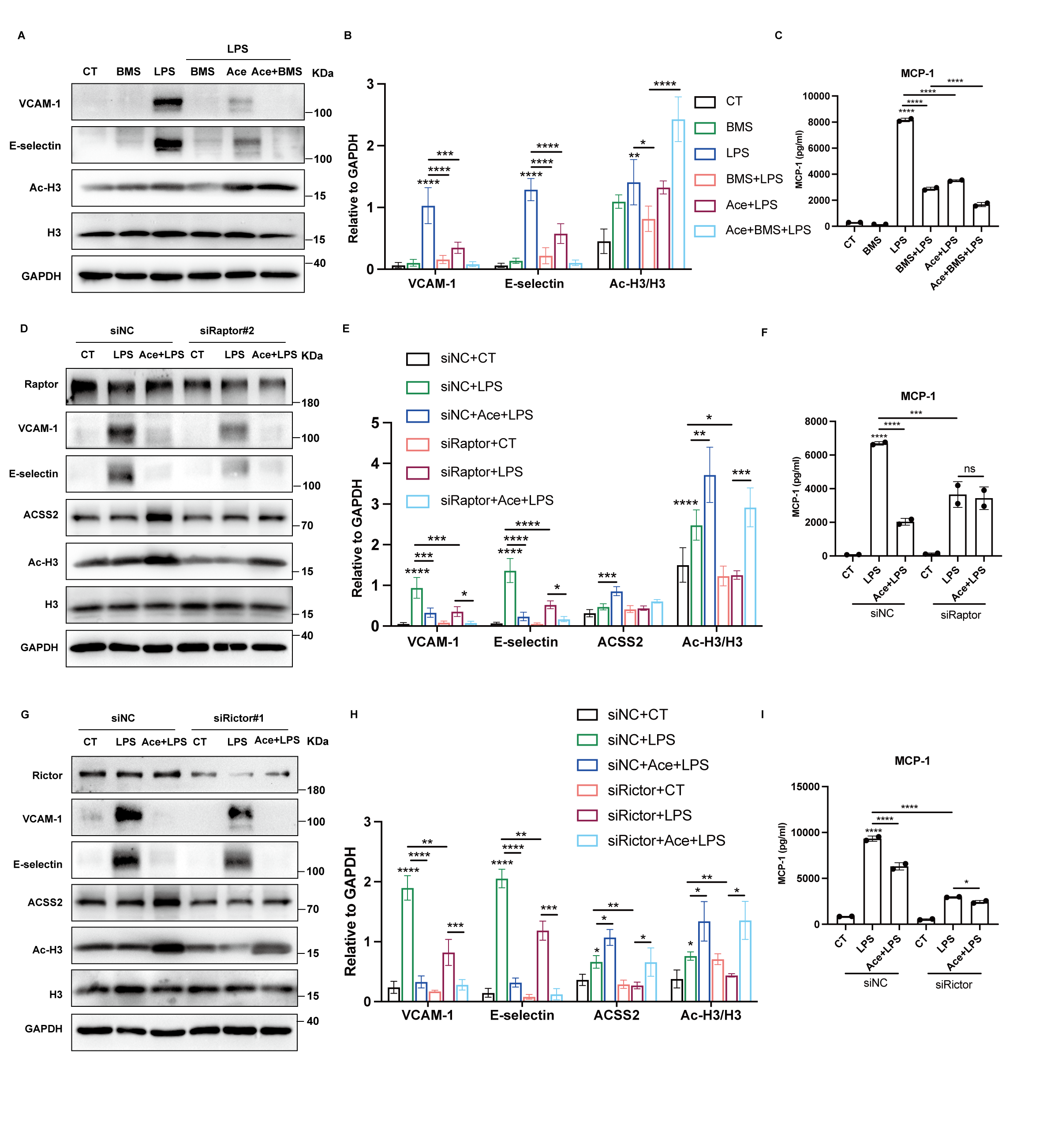

Supplement: Supplementary file 6 — Figure S5 [file 41419_2023_5932_MOESM6_ESM.tif]
