# Supplementary material for: ATP-citrate lyase controls endothelial gluco-lipogenic metabolism and vascular inflammation in sepsis-associated organ injury
Source: Cell Death Dis. 2023 Jul 6;14(7):401. doi: 10.1038/s41419-023-05932-8 (PMC10325983; doi:10.1038/s41419-023-05932-8)
Supplement: Supplementary file 7 — Original data [file 41419_2023_5932_MOESM7_ESM.pdf]

ATP-citrate lyase controls endothelial gluco-lipogenic metabolism and vascular inflammation in sepsis-associated organ injury

Ranran Li[1,6]*, Mei Meng[1,6], Ying Chen[2,6], Tingting Pan[1], Yinjiaozhi Li[1], Yunxin Deng[1], Ruyuan Zhang[1], Rui Tian[1], Wen Xu[1], Xiangtao Zheng[2], Fangchen Gong[2], Jie Liu[3], Haiting Tang[4], Xiaowei Ding[4], Yaoqing Tang[1], Djillali Annane[5], Erzhen Chen[2]*, Hongping Qu[1]*, Lei Li[1]*

Raw data for western blot

Figure 2a: Lung

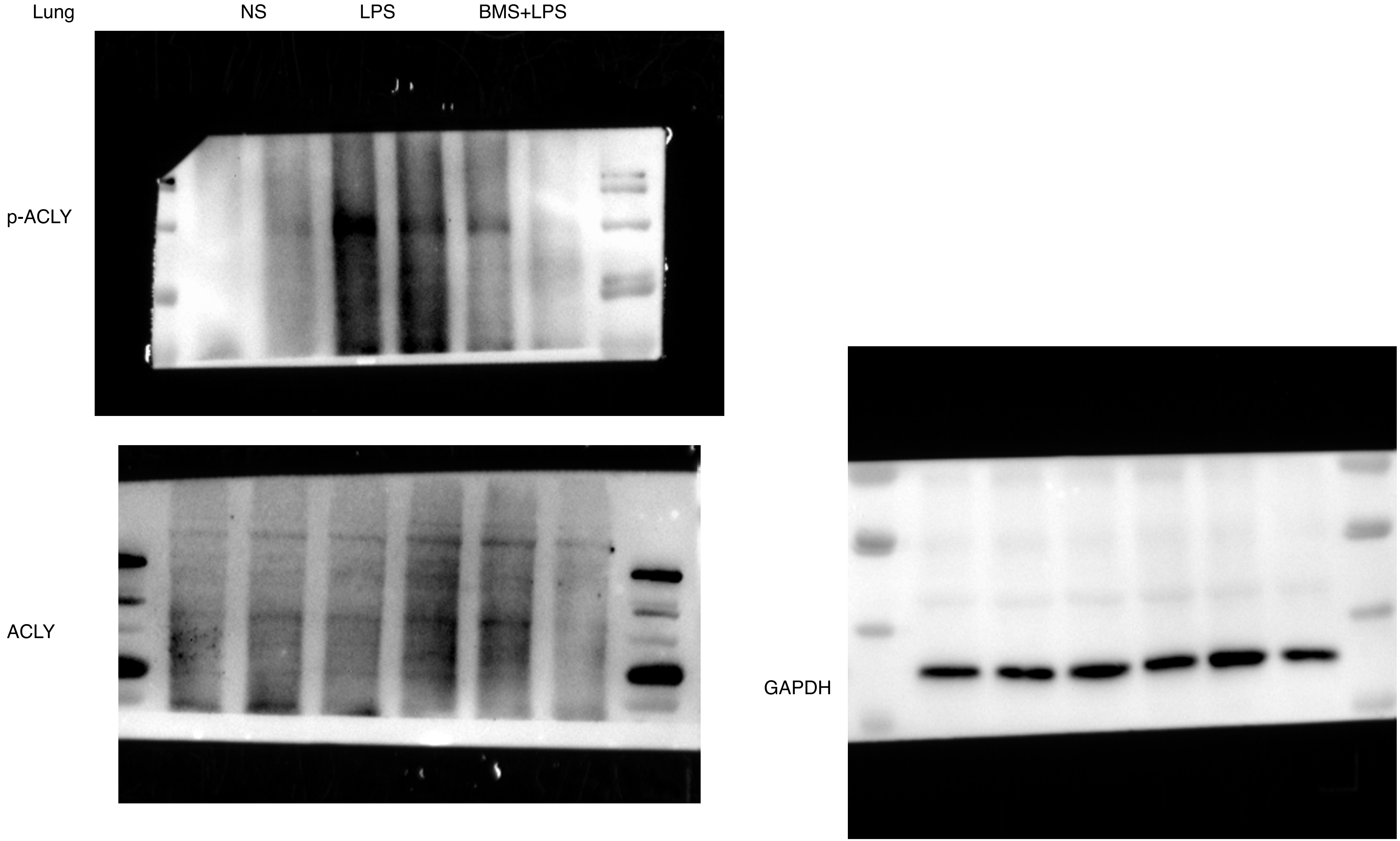

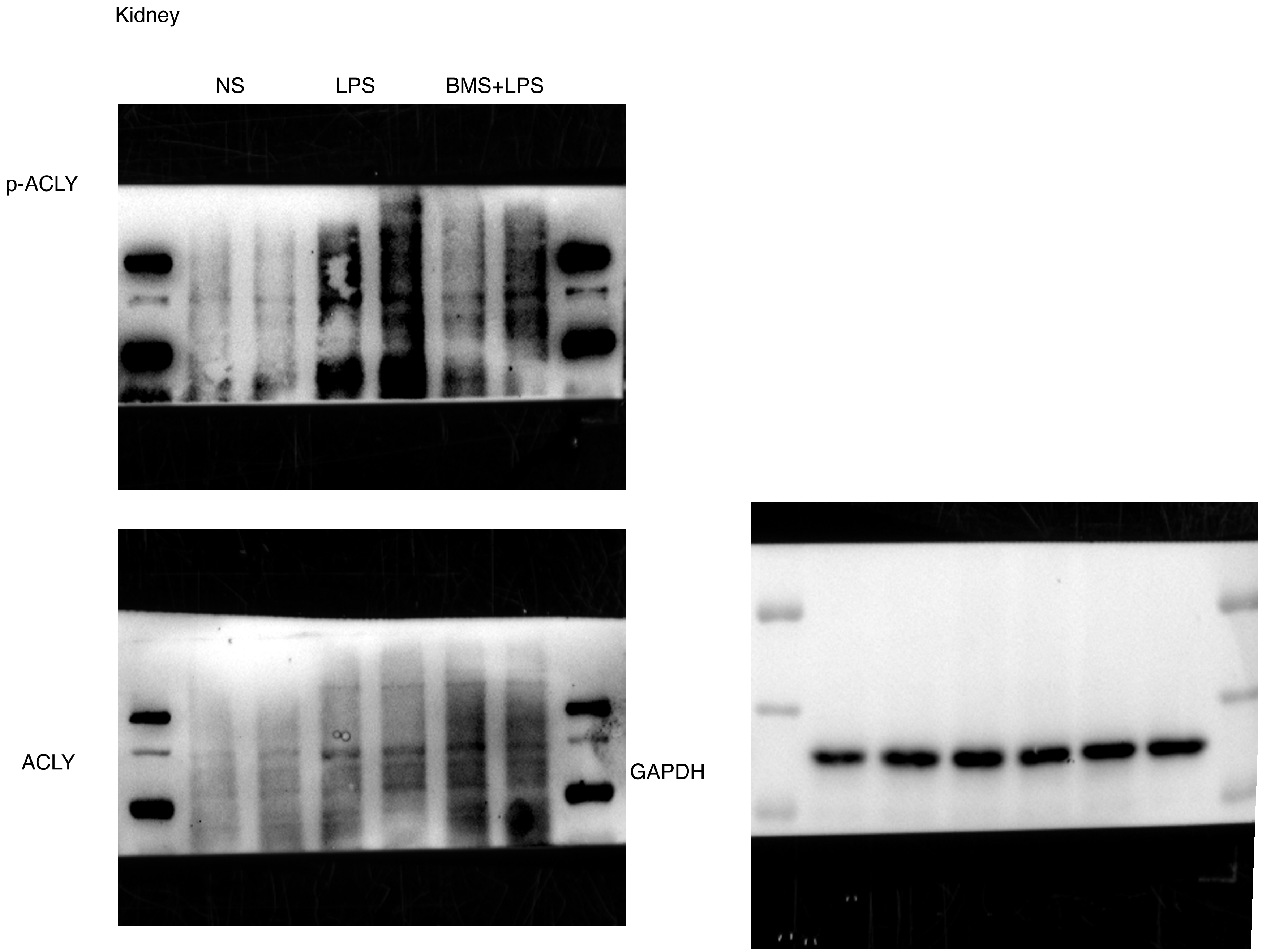
Kidney
NS
LPS
BMS+LPS
p-ACLY
ACLY
GAPDH

liver

NS LPS BMS+LPS

p-ACLY

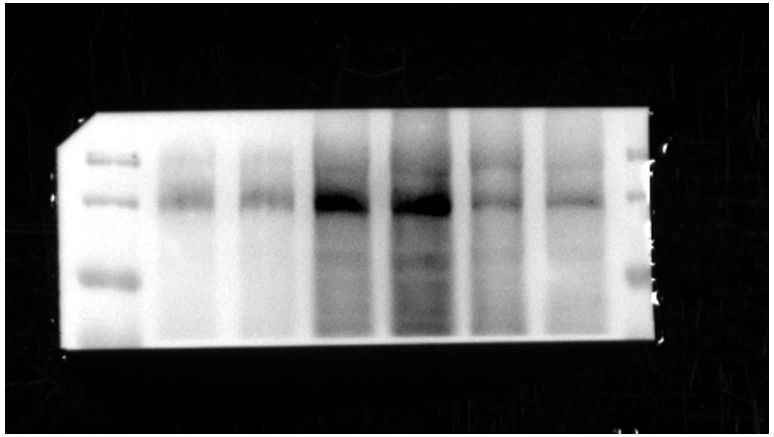

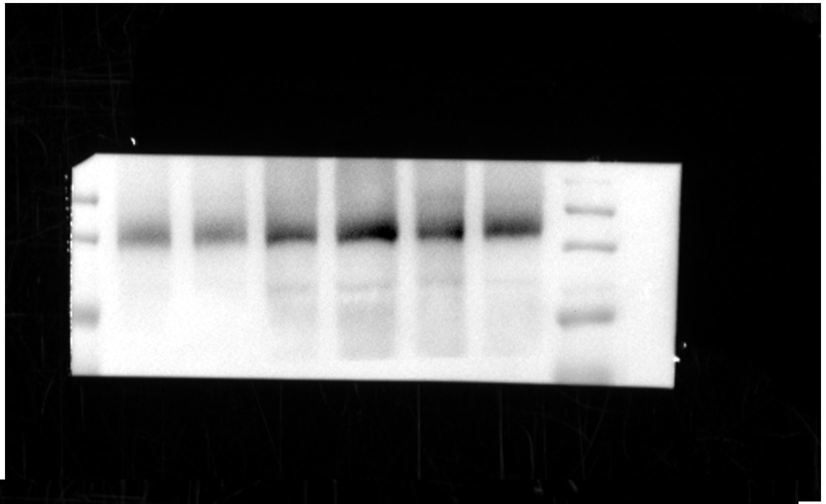

ACLY

GAPDH

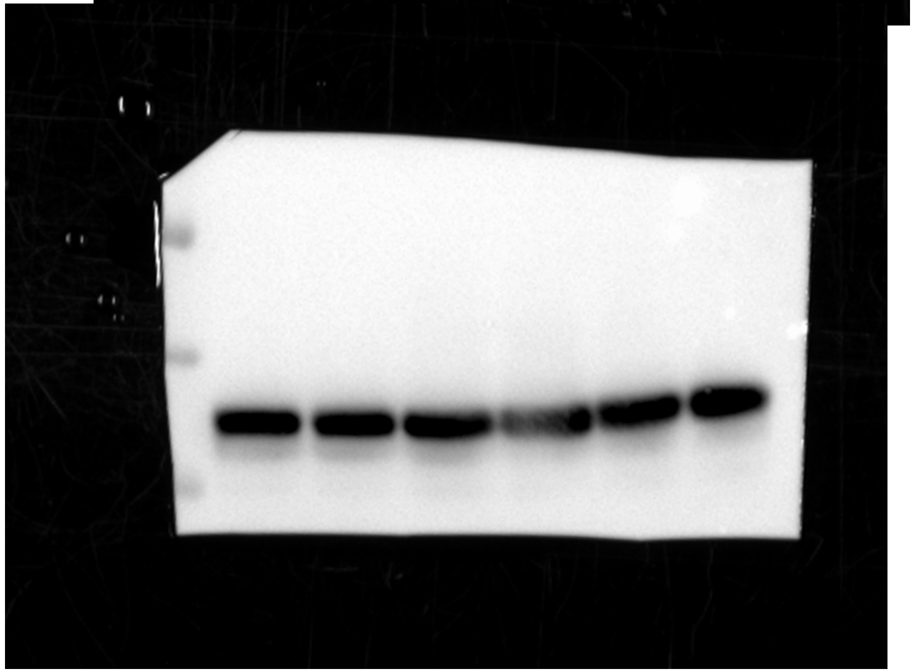

Figure 3a

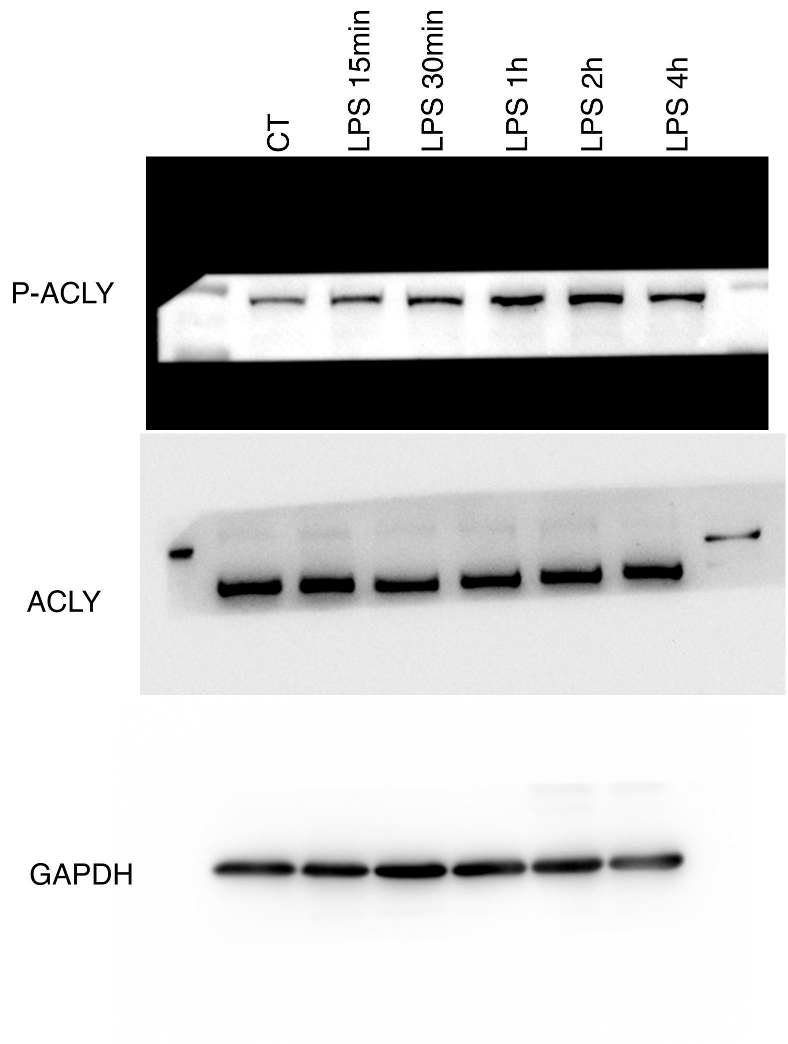

Figure 3c

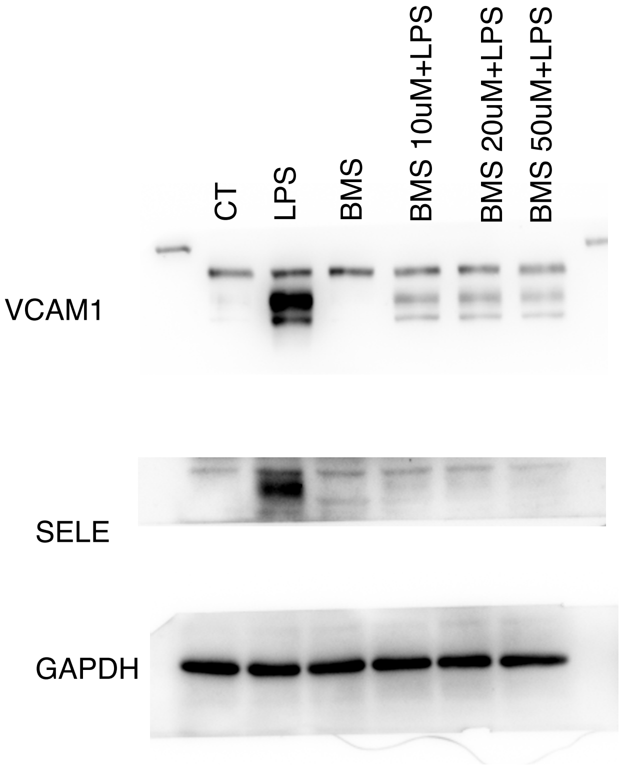
CT
LPS
BMS
BMS 10uM+LPS
BMS 20uM+LPS
BMS 50uM+LPS
VCAM1
SELE
GAPDH

Figure 3e.

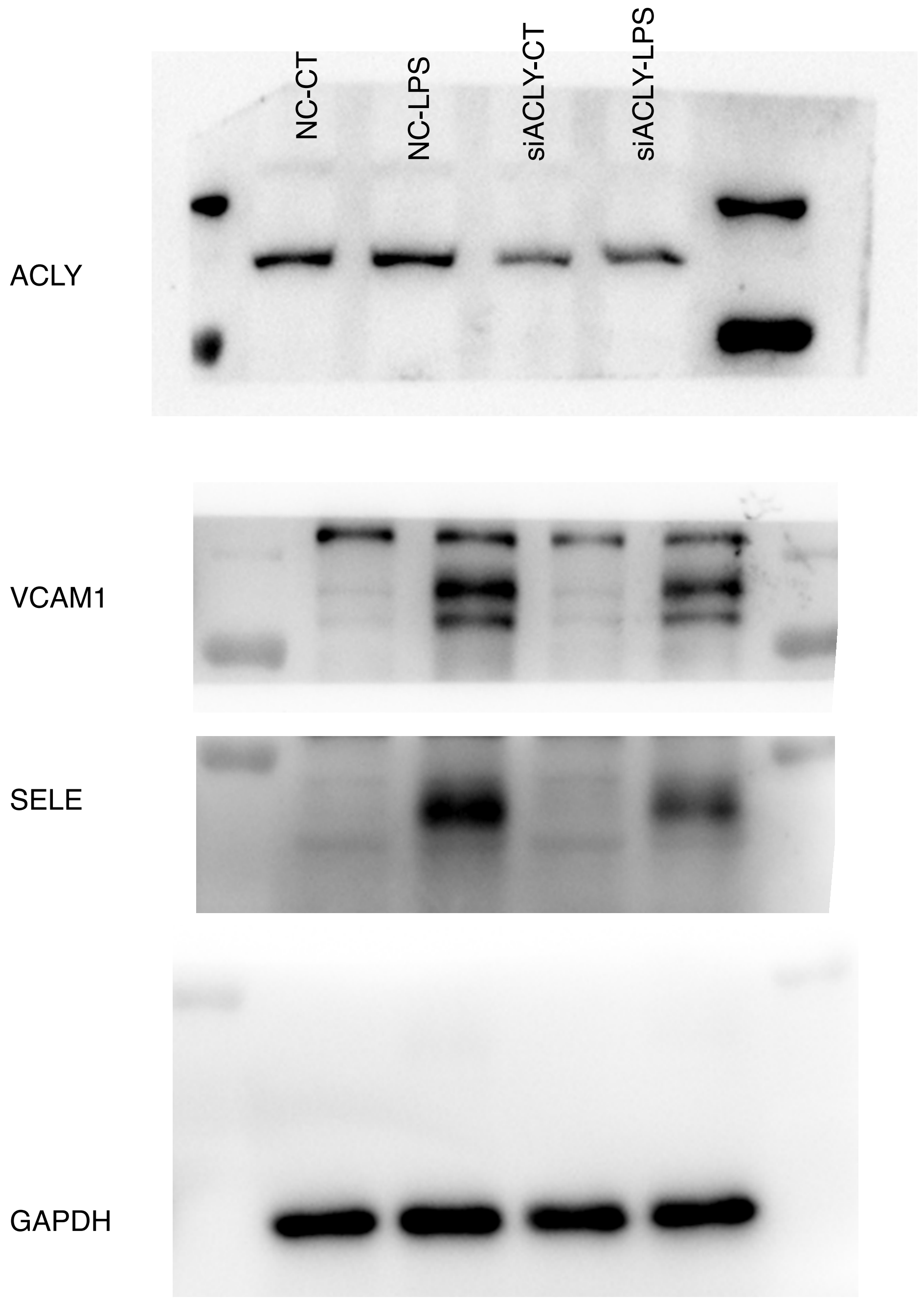

Figure 3K.

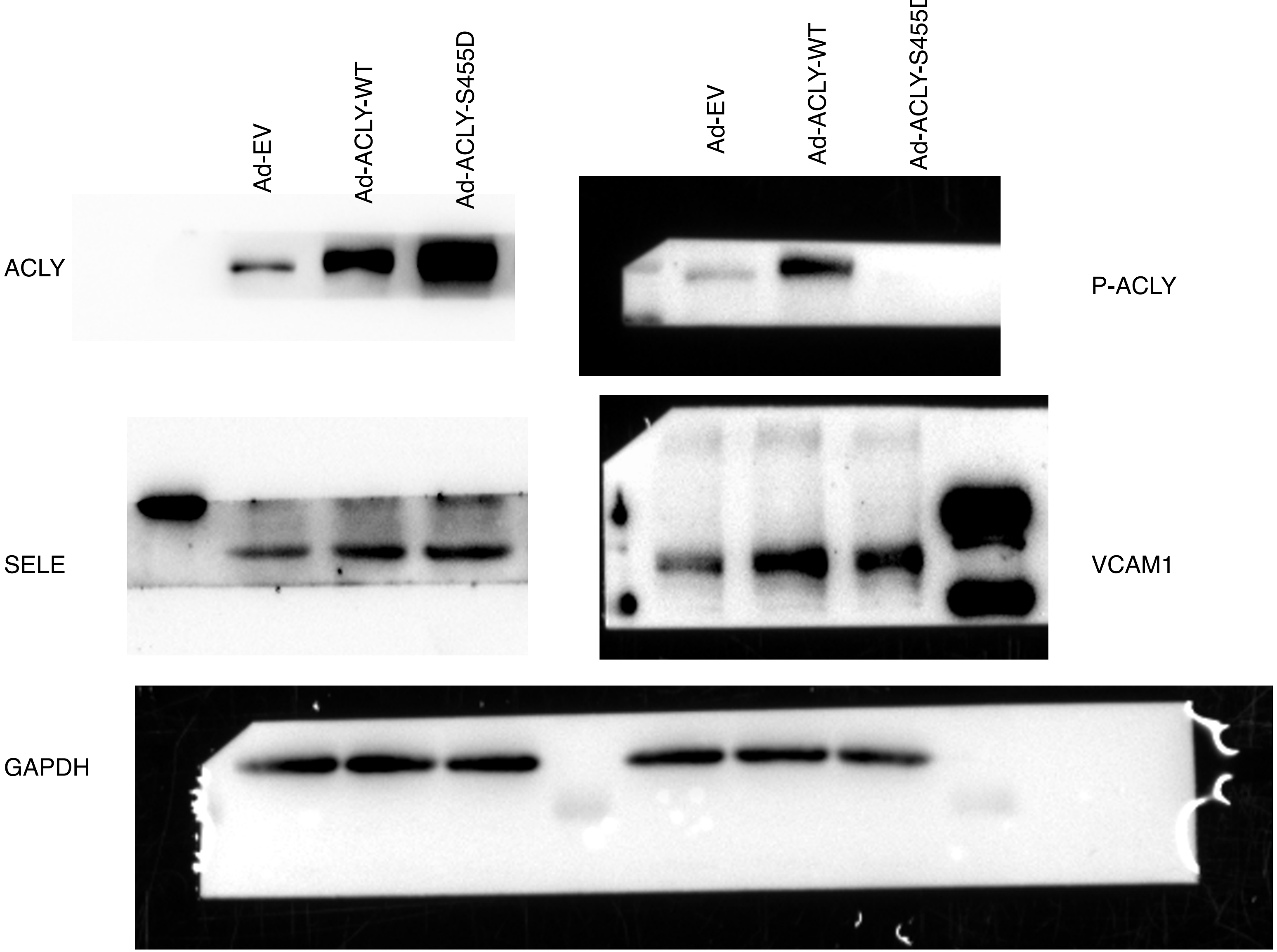
Ad-EV
Ad-ACLY-WT
Ad-ACLY-S455D
Ad-EV
Ad-ACLY-WT
Ad-ACLY-S455D
ACLY
P-ACLY
SELE
VCAM1
GAPDH

Figure 4C

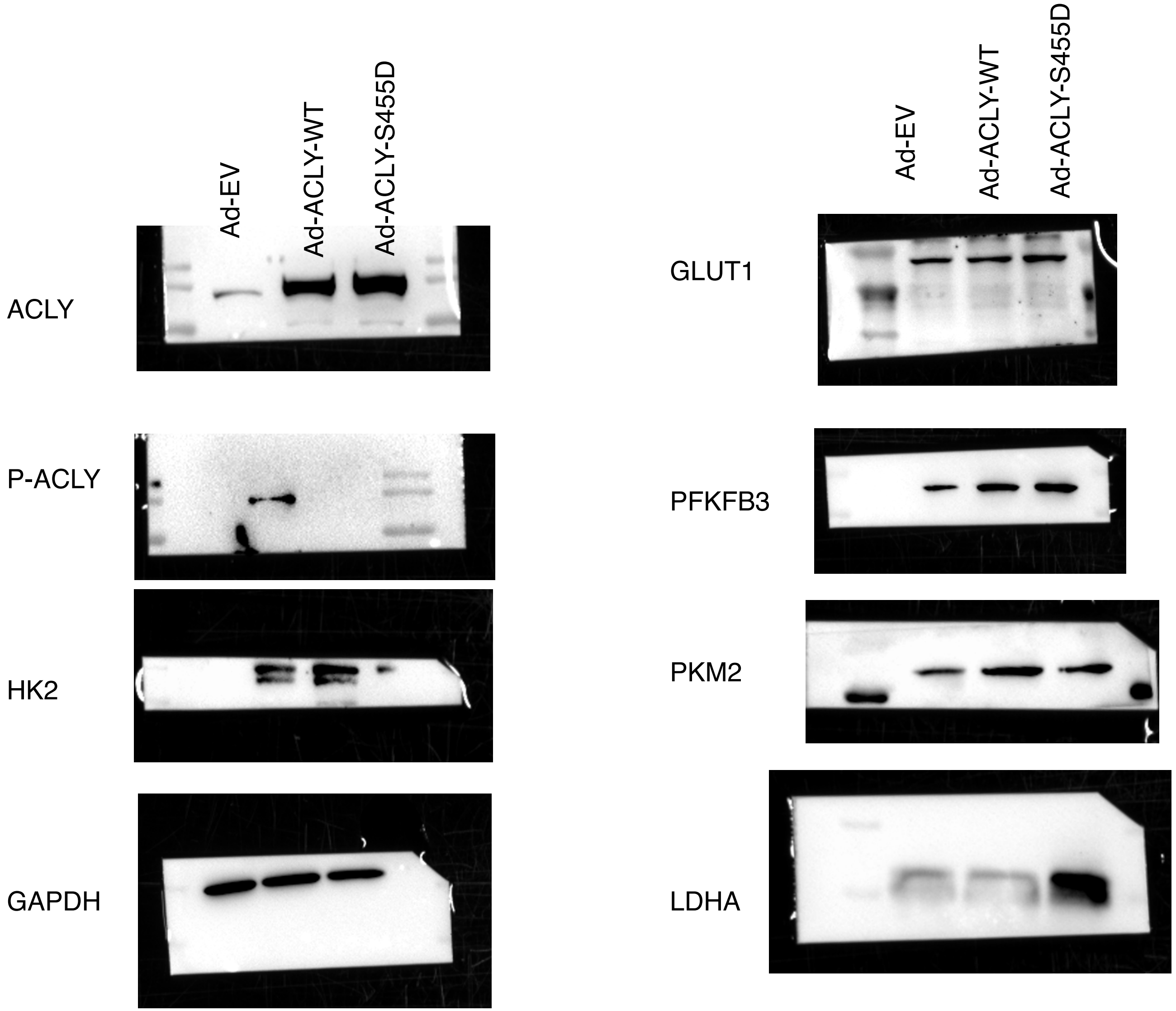
Ad-EV
Ad-ACLY-WT
Ad-ACLY-S455D
ACLY
P-ACLY
HK2
GAPDH
Ad-EV
Ad-ACLY-WT
Ad-ACLY-S455D
GLUT1
PFKFB3
PKM2
LDHA

Figure 4E

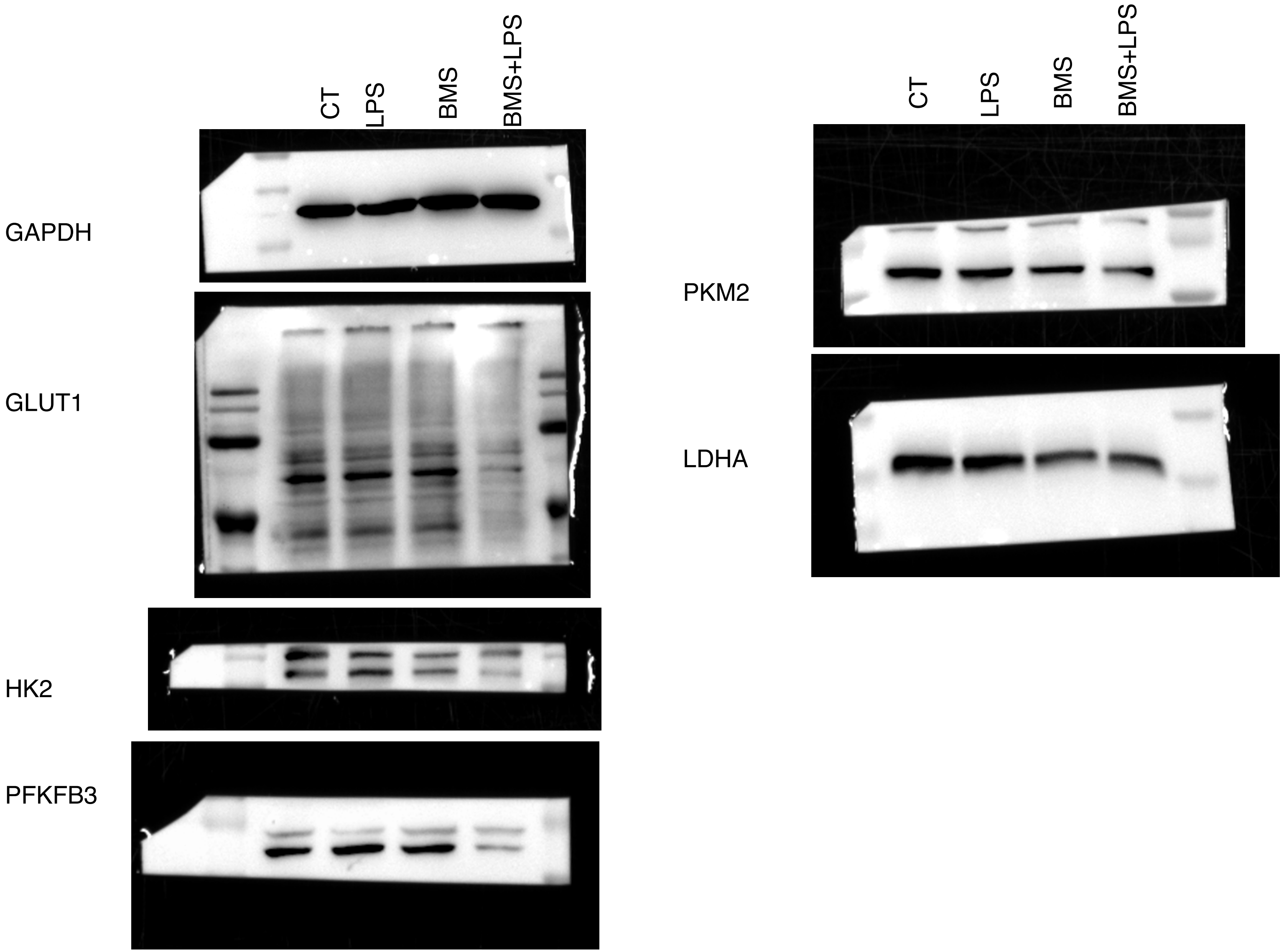
CT
LPS
BMS
BMS+LPS
GAPDH
GLUT1
HK2
PFKFB3
CT
LPS
BMS
BMS+LPS
PKM2
LDHA

Figure 4K

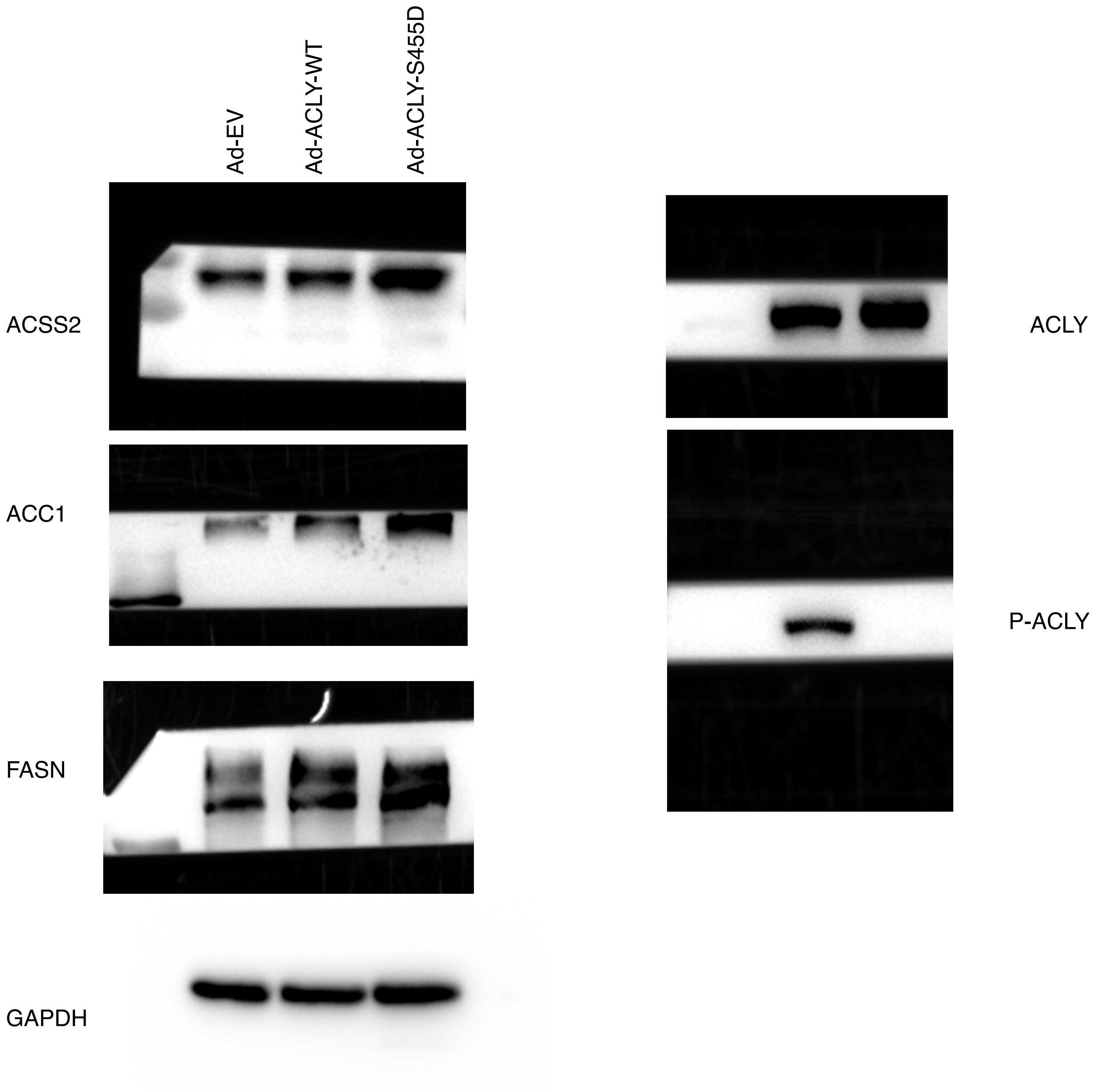
Ad-EV
Ad-ACLY-WT
Ad-ACLY-S455D
ACSS2
ACC1
FASN
GAPDH
ACLY
P-ACLY

Figure 4M

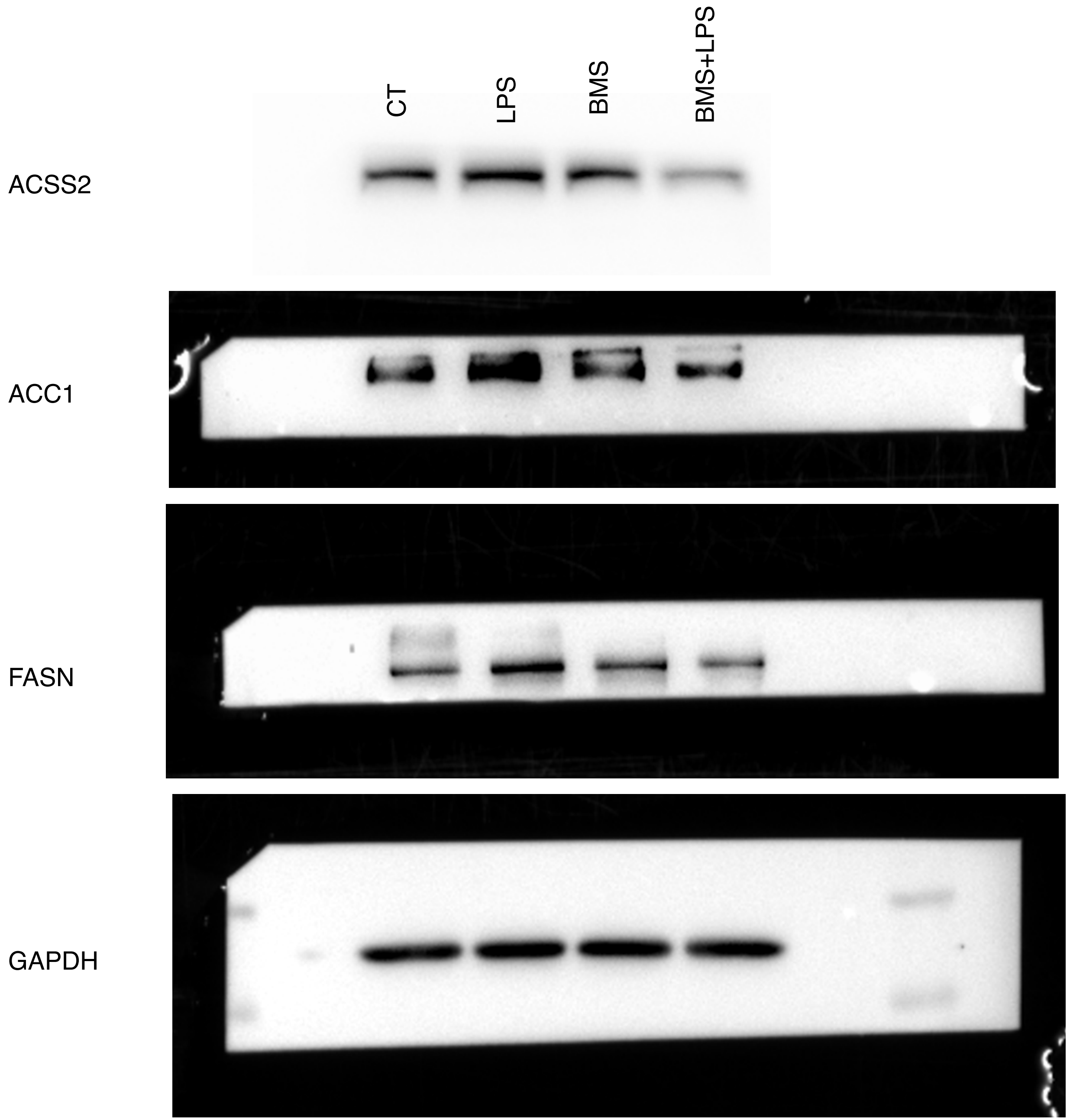

Figure 5D

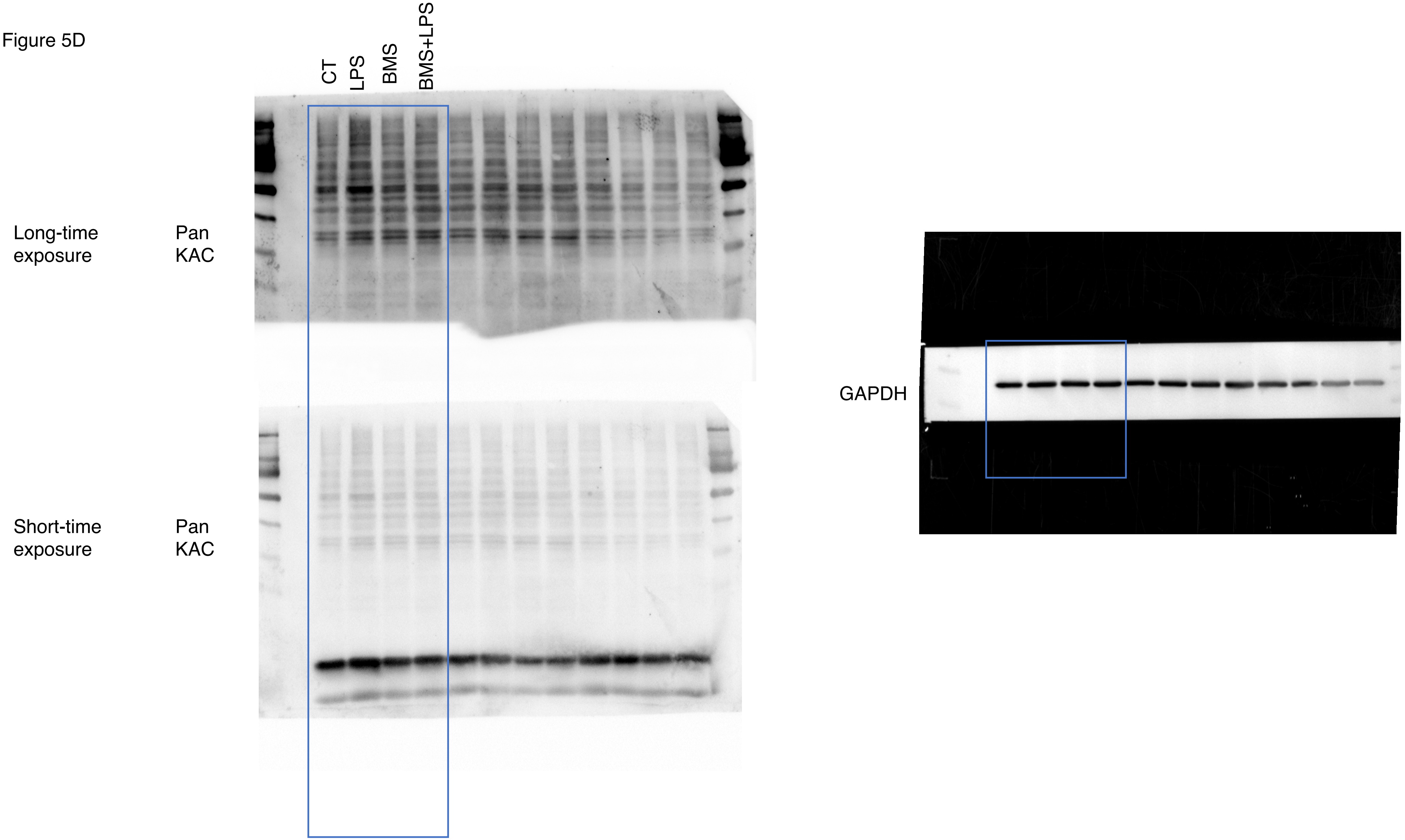
CT
LPS
BMS
BMS+LPS
Long-time exposure
Pan KAC
Short-time exposure
Pan KAC
GAPDH

Figure 5E

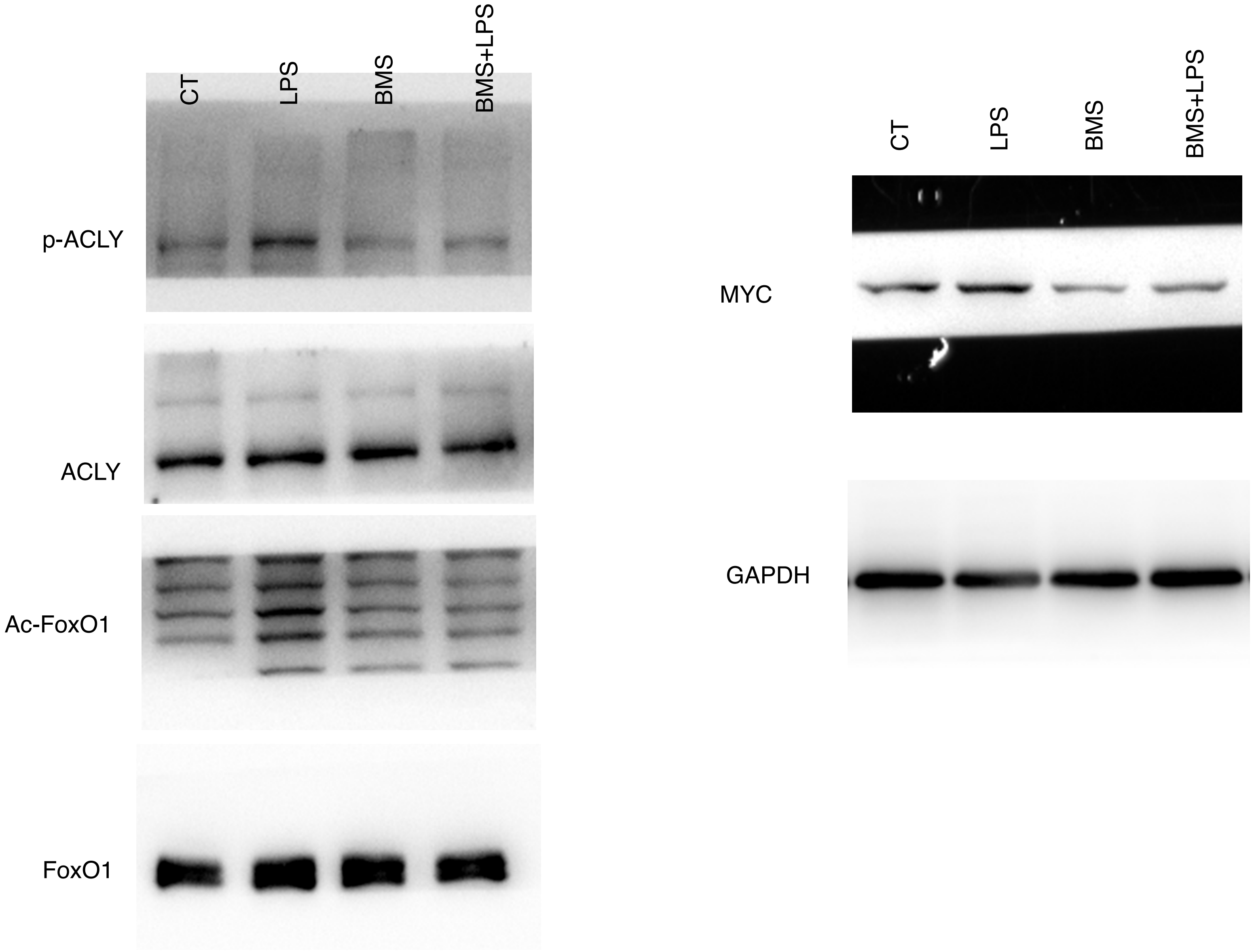
CT
LPS
BMS
BMS+LPS
p-ACLY
ACLY
Ac-FoxO1
FoxO1
CT
LPS
BMS
BMS+LPS
MYC
GAPDH

Figure 5G

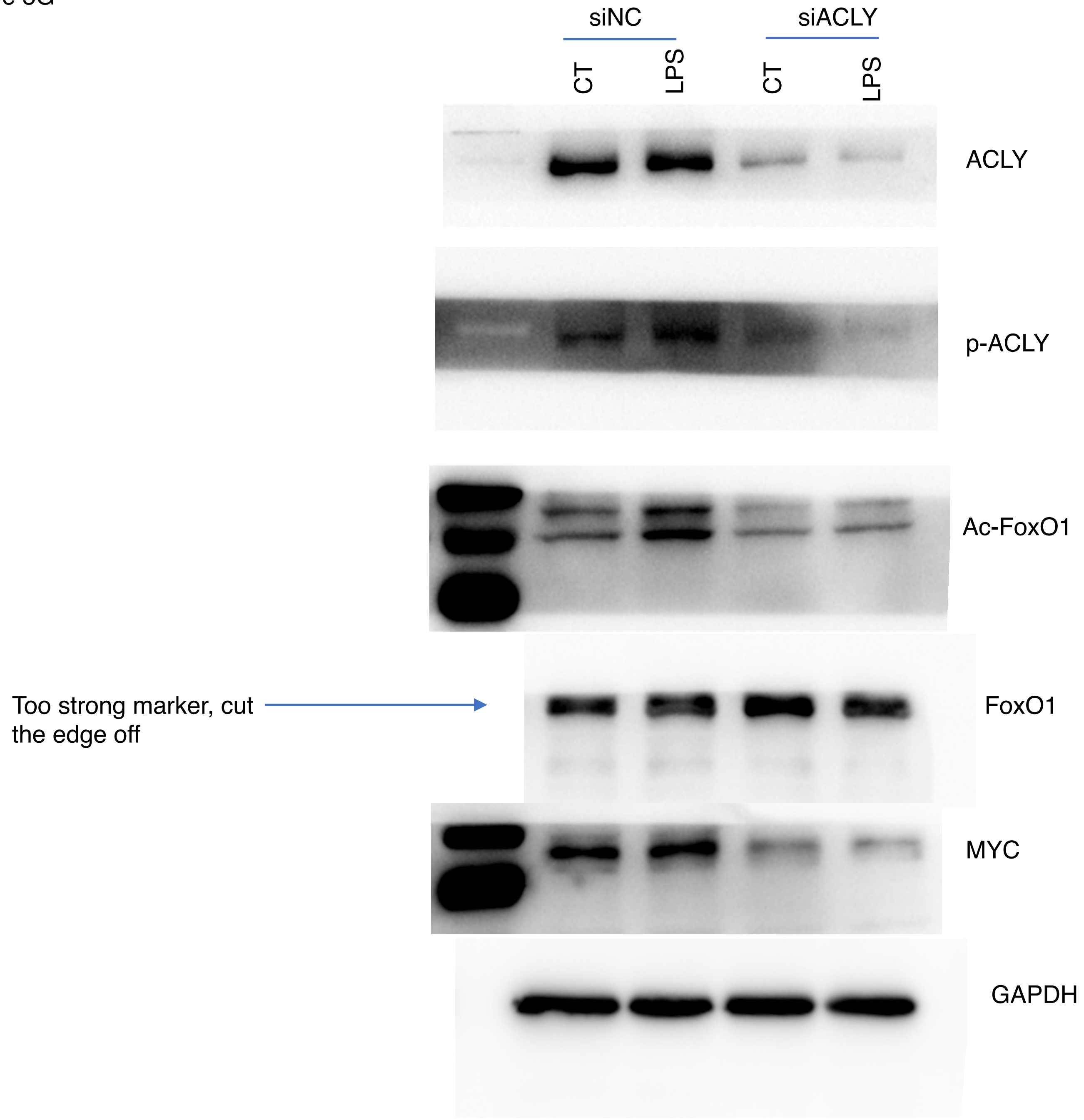
siNC
siACLY
CT
LPS
CT
LPS
ACLY
p-ACLY
Ac-FoxO1
FoxO1
MYC
GAPDH
Too strong marker, cut the edge off

Figure 5J

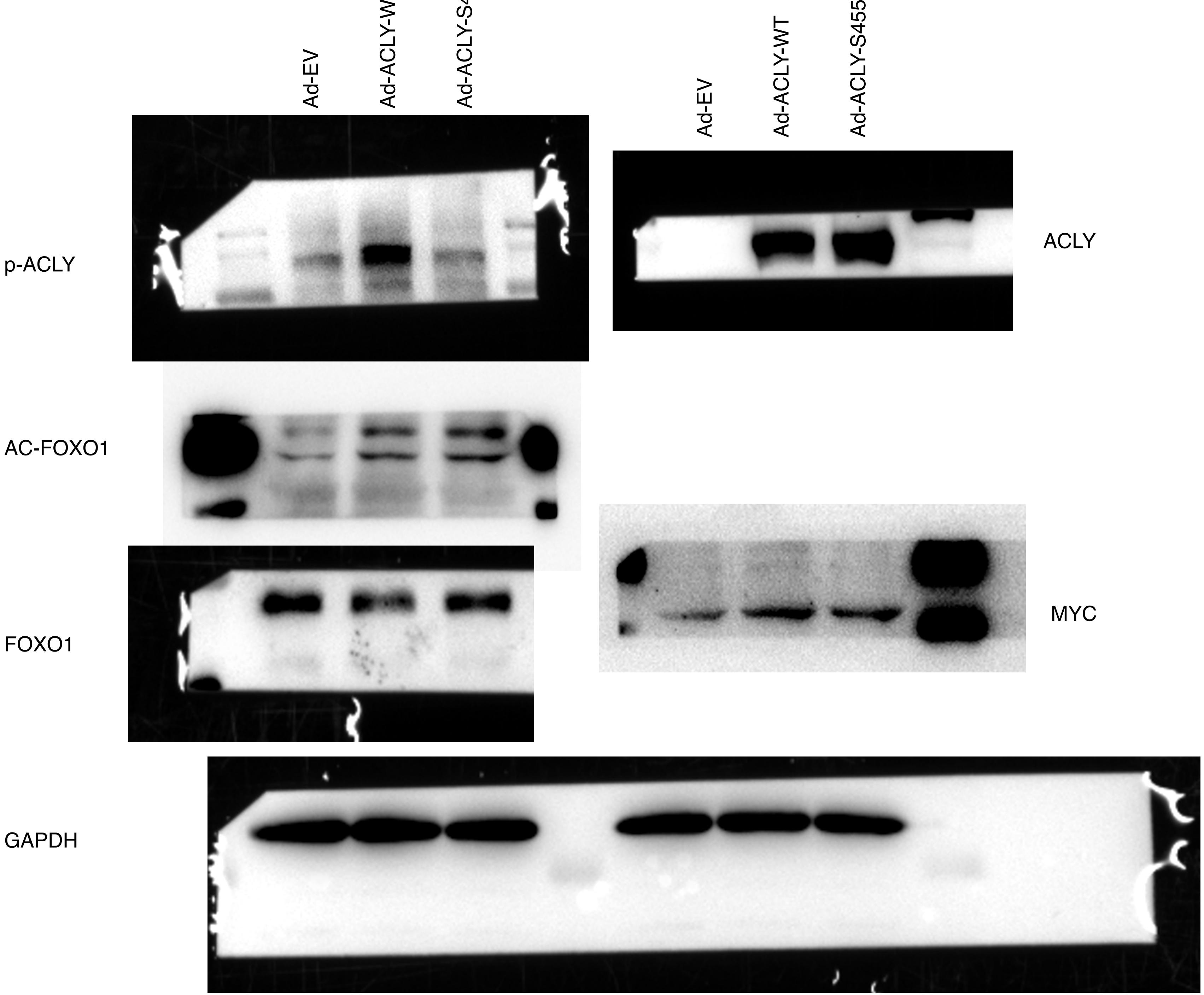
Ad-EV
Ad-ACLY-W
Ad-ACLY-S4
Ad-EV
Ad-ACLY-WT
Ad-ACLY-S455
p-ACLY
ACLY
AC-FOXO1
FOXO1
MYC
GAPDH

Figure 5M

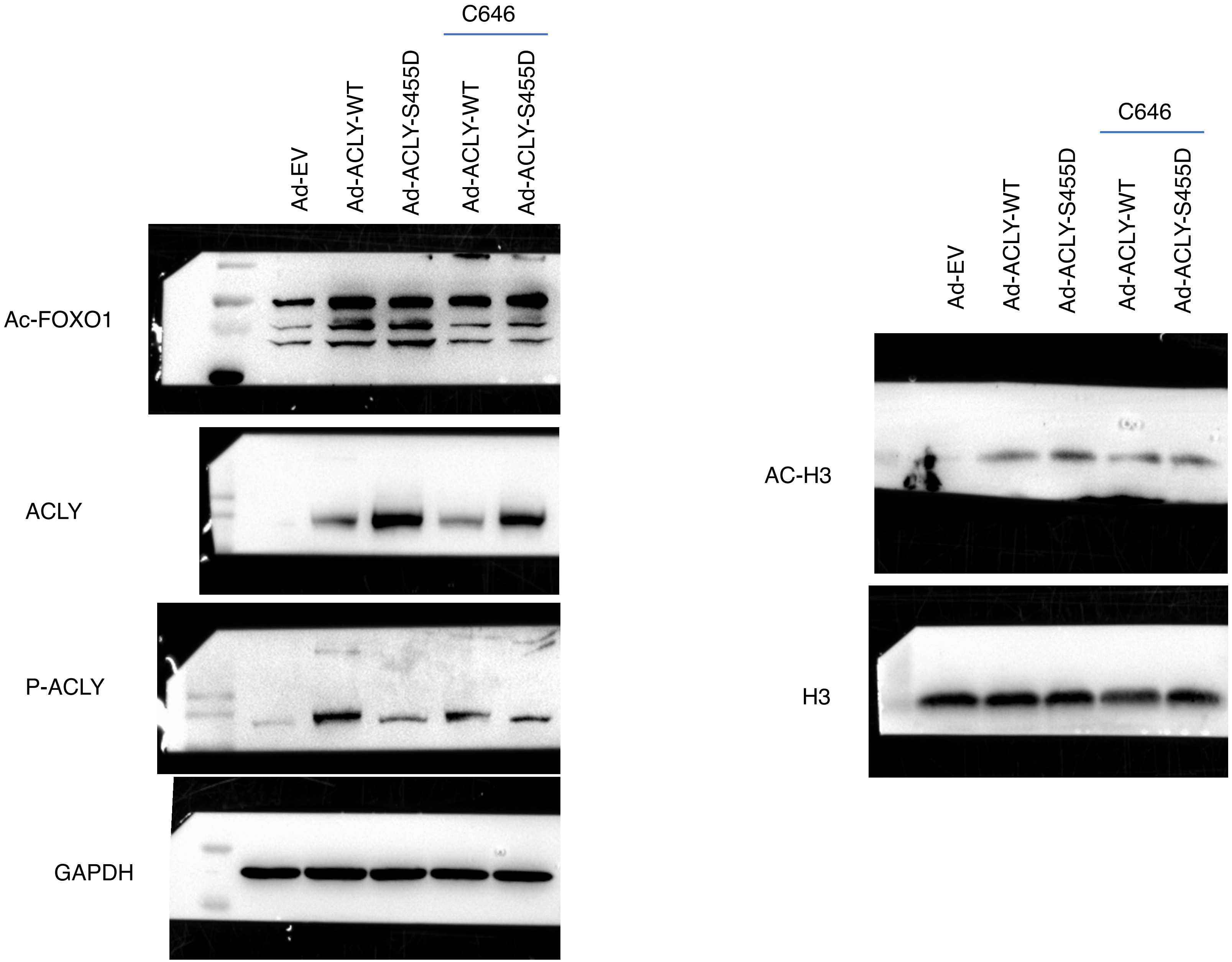

C646
Ad-EV
Ad-ACLY-WT
Ad-ACLY-S455D
Ad-ACLY-WT
Ad-ACLY-S455D
Ac-FOXO1
ACLY
P-ACLY
GAPDH
C646
Ad-EV
Ad-ACLY-WT
Ad-ACLY-S455D
Ad-ACLY-WT
Ad-ACLY-S455D
AC-H3
H3

Figure 6A

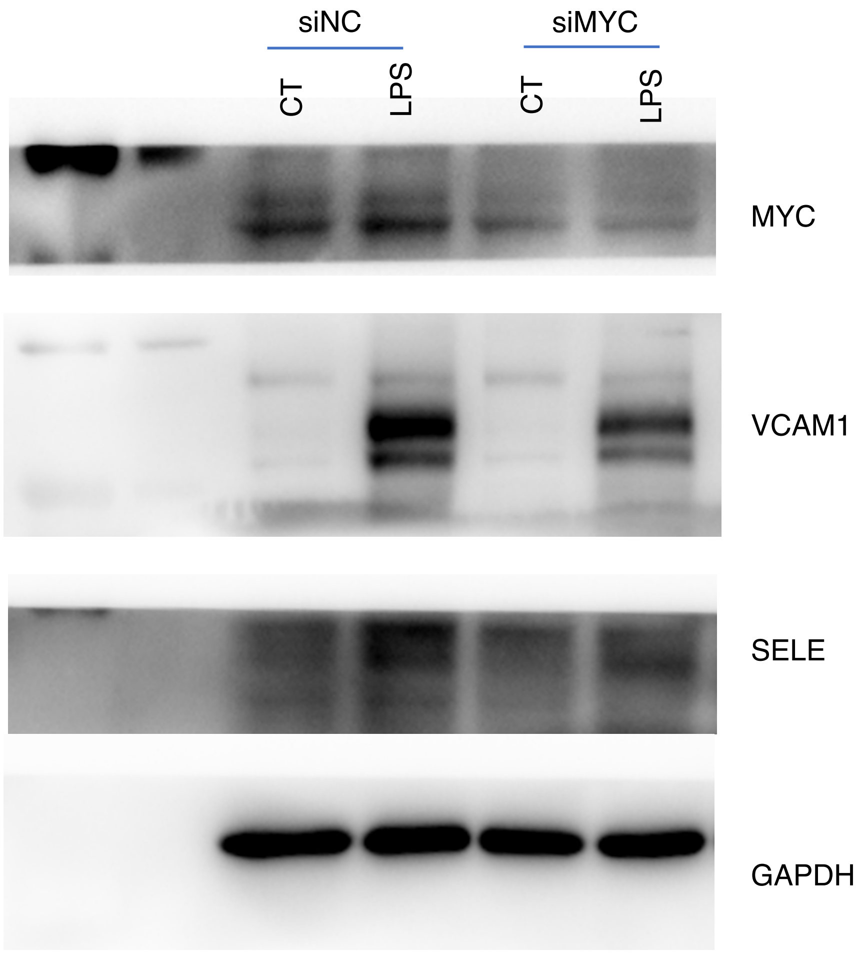

Figure 6C

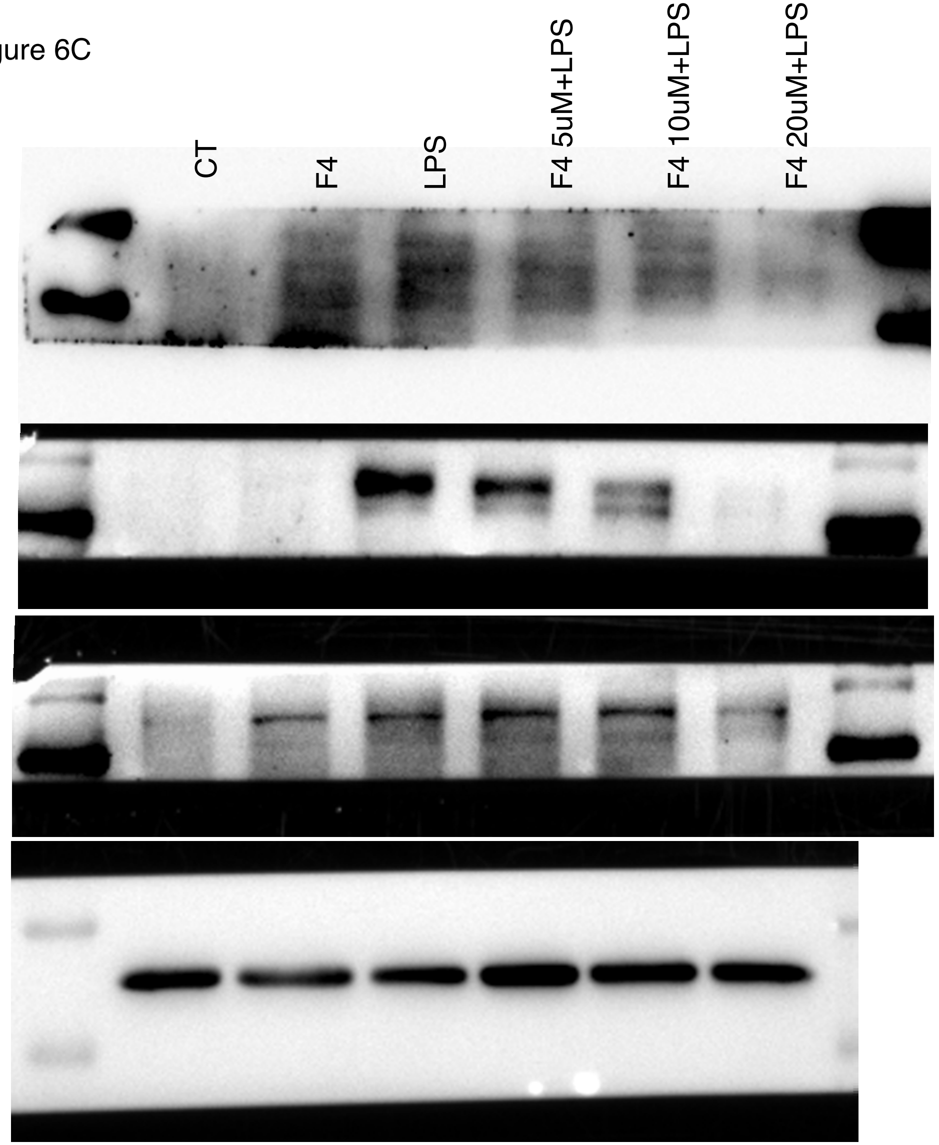

Figure 6F

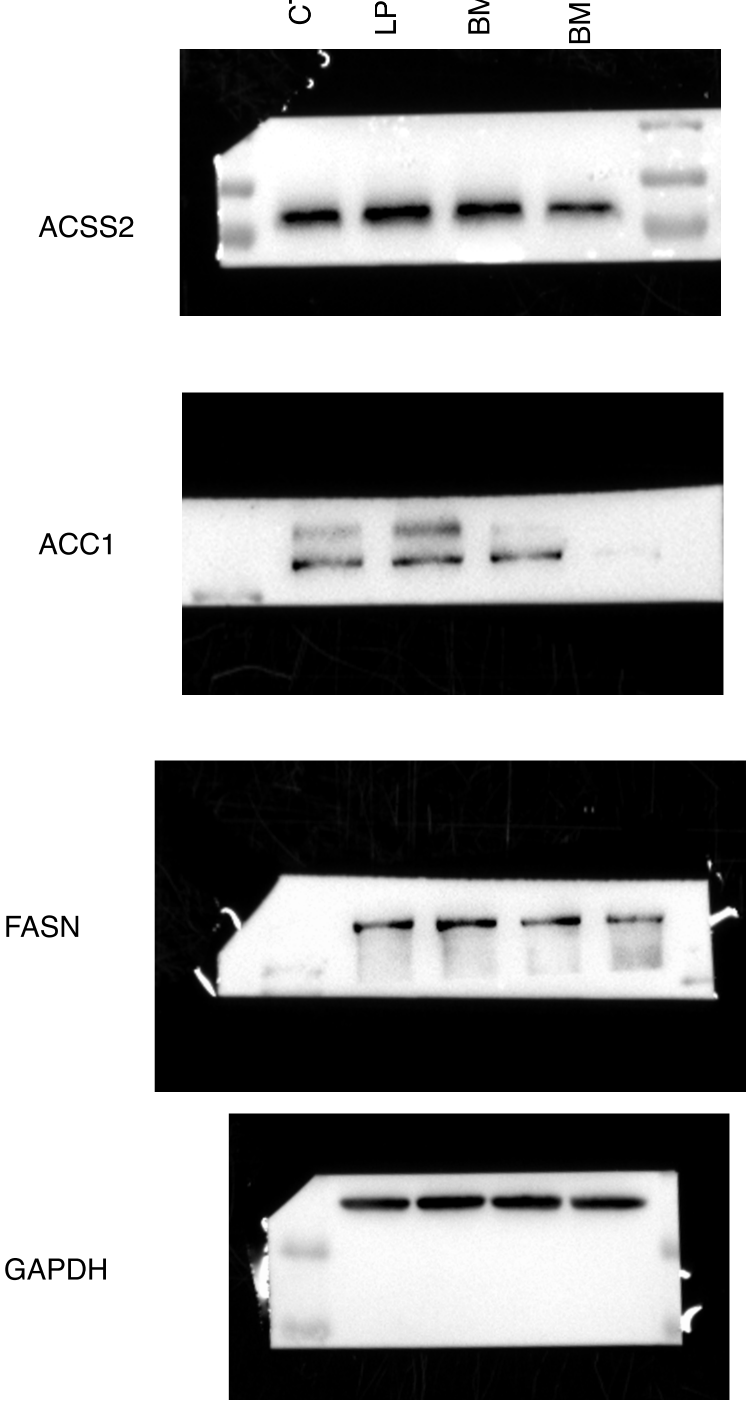

Figure 6H

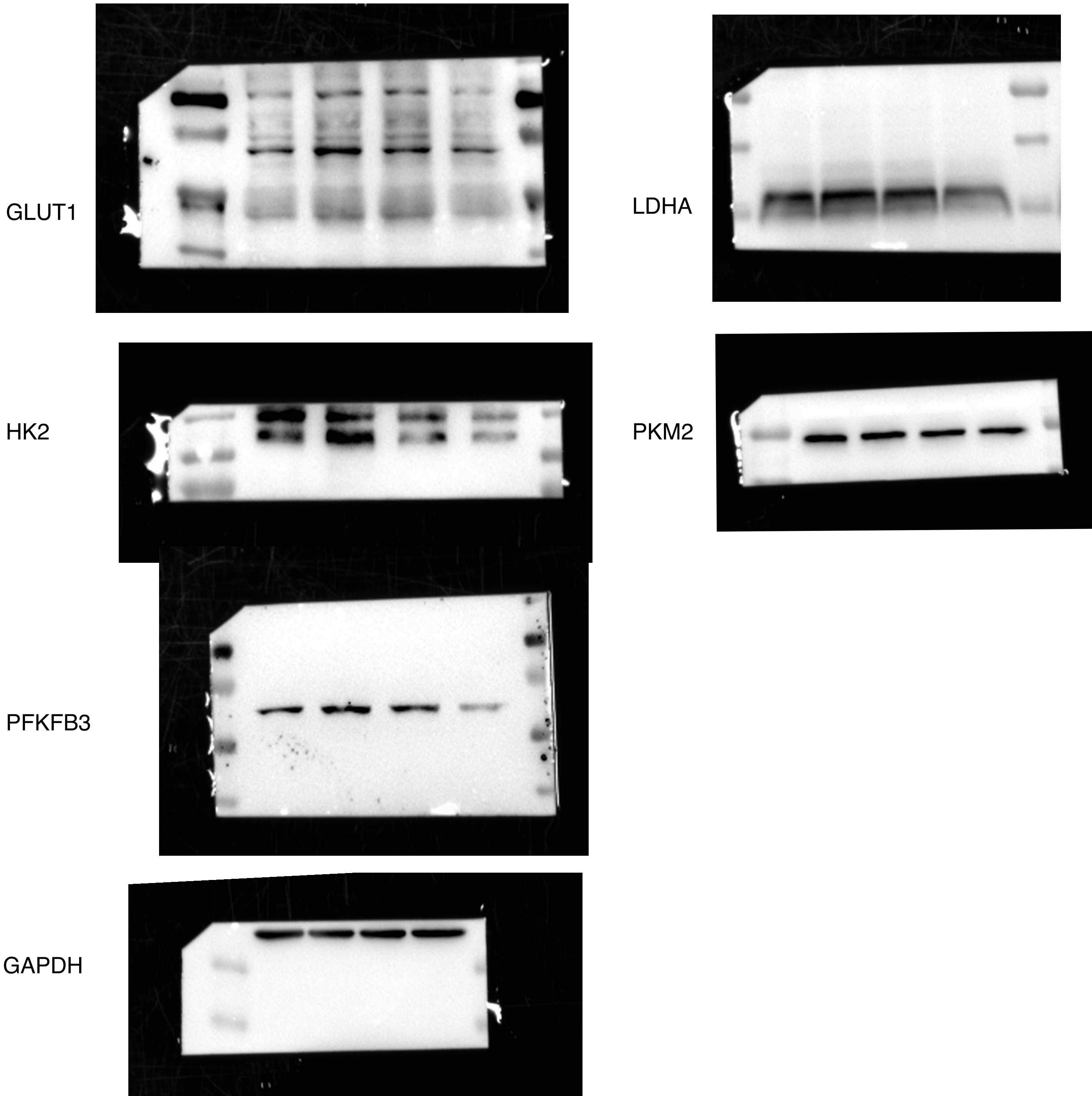

Figure 6M

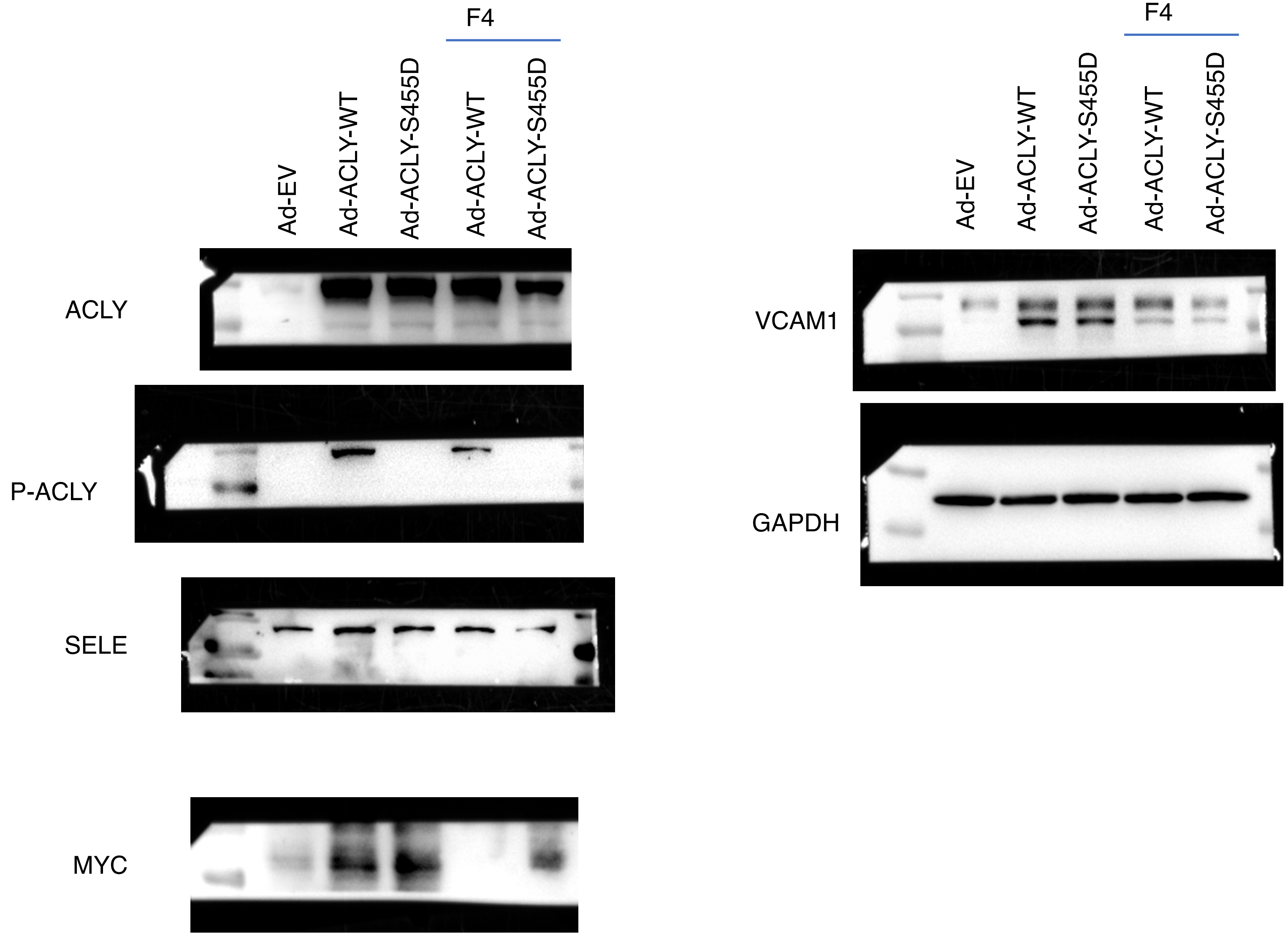
F4
Ad-EV
Ad-ACLY-WT
Ad-ACLY-S455D
Ad-ACLY-WT
Ad-ACLY-S455D
ACLY
P-ACLY
SELE
MYC
F4
Ad-EV
Ad-ACLY-WT
Ad-ACLY-S455D
Ad-ACLY-WT
Ad-ACLY-S455D
VCAM1
GAPDH

Figure 7A

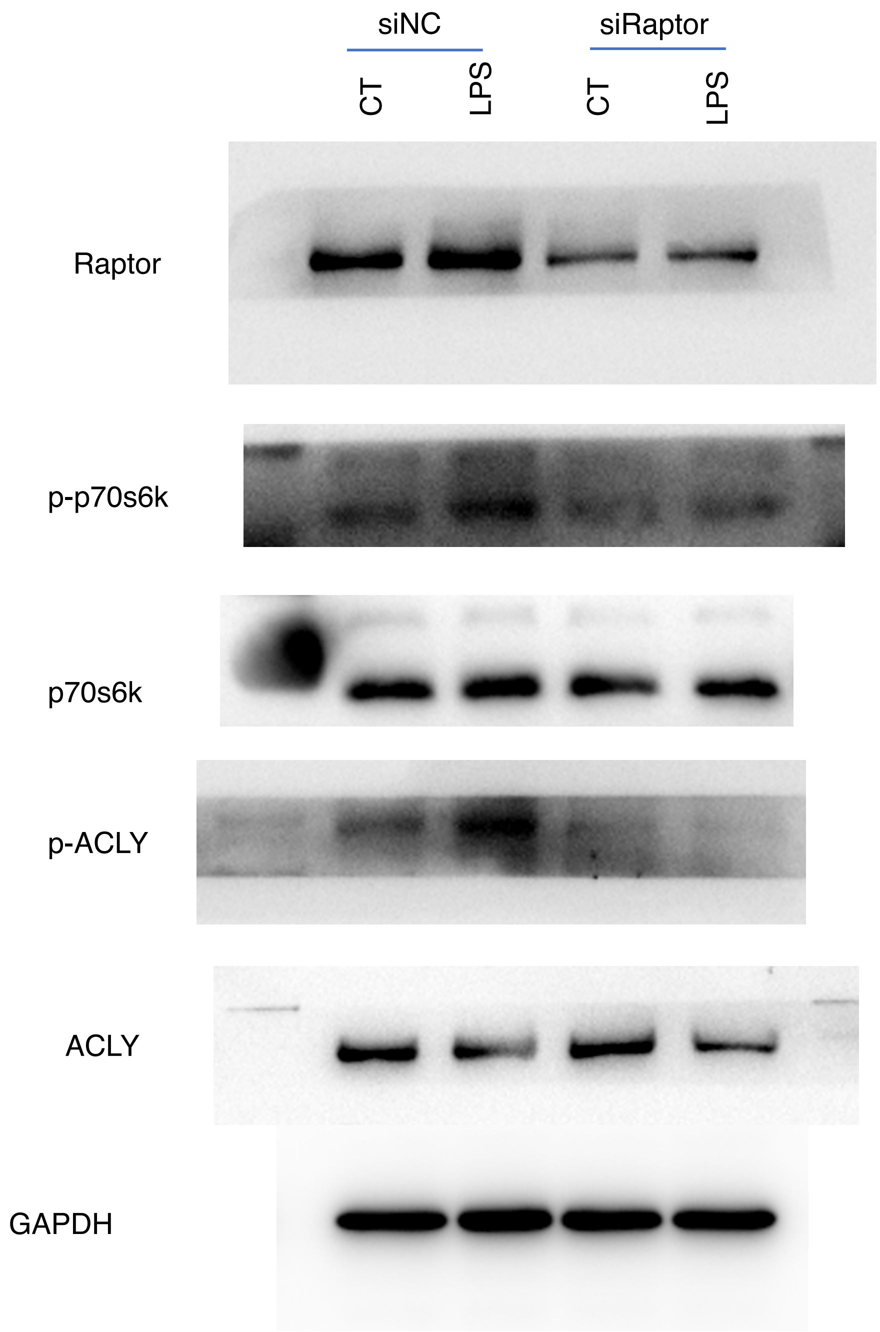
siNC
siRaptor
CT
LPS
CT
LPS
Raptor
p-p70s6k
p70s6k
p-ACLY
ACLY
GAPDH

Figure 7C

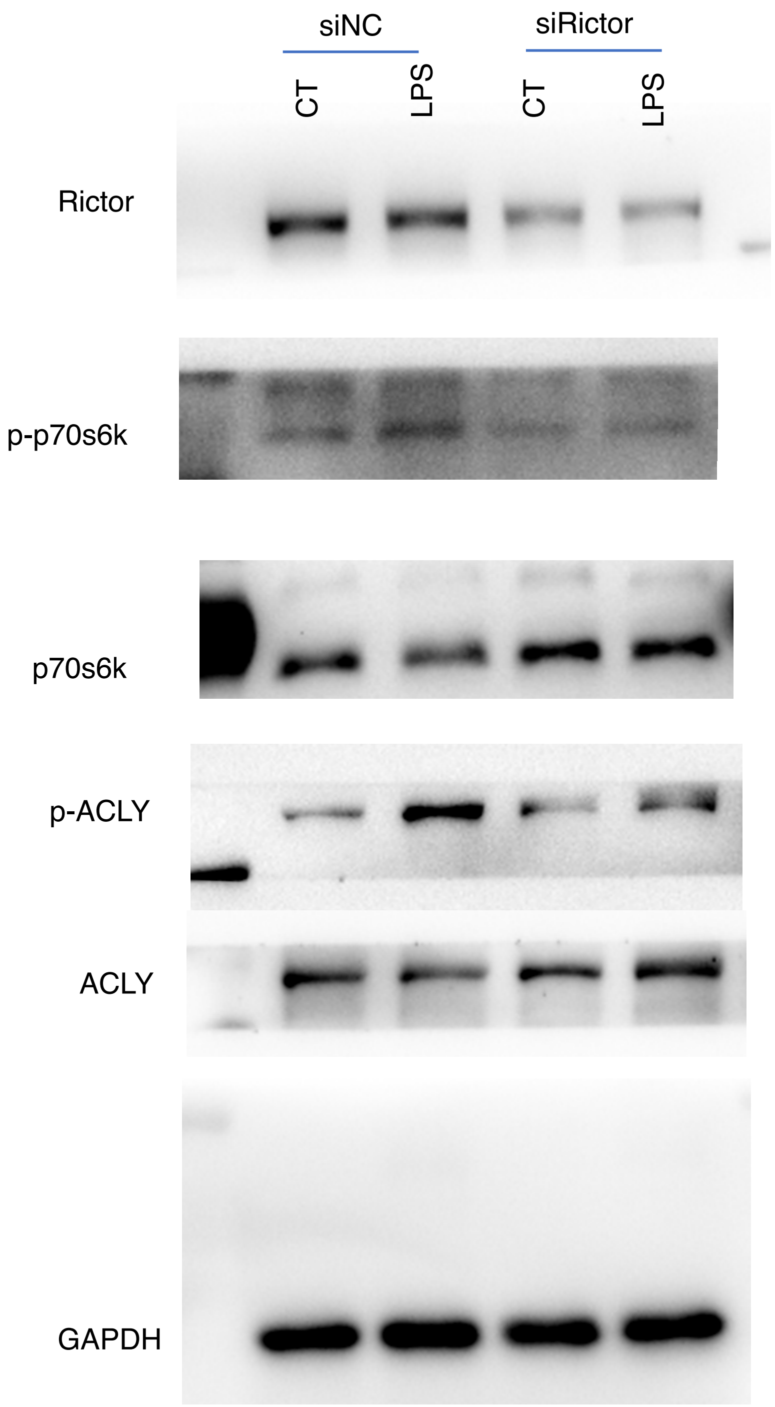
siNC
siRictor
CT
LPS
CT
LPS
Rictor
p-p70s6k
p70s6k
p-ACLY
ACLY
GAPDH

Figure 7F

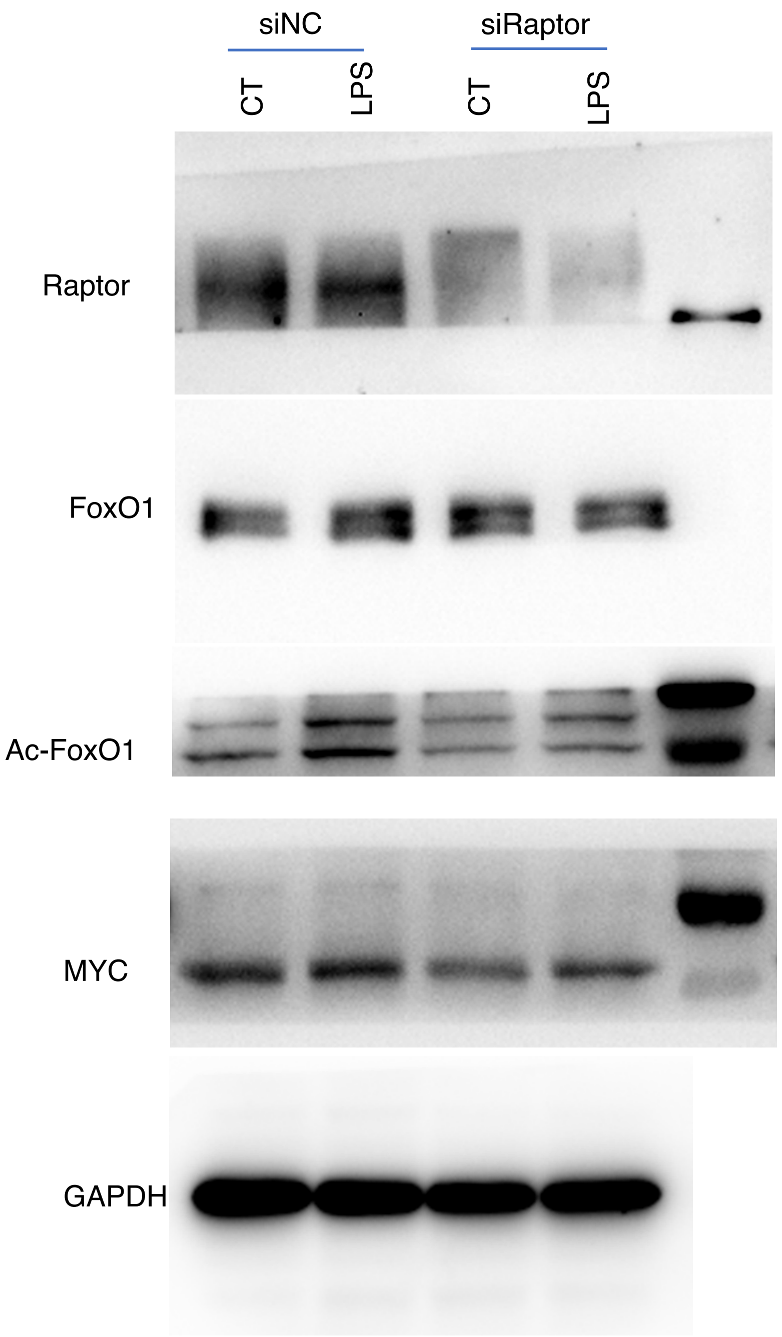

Figure 7H

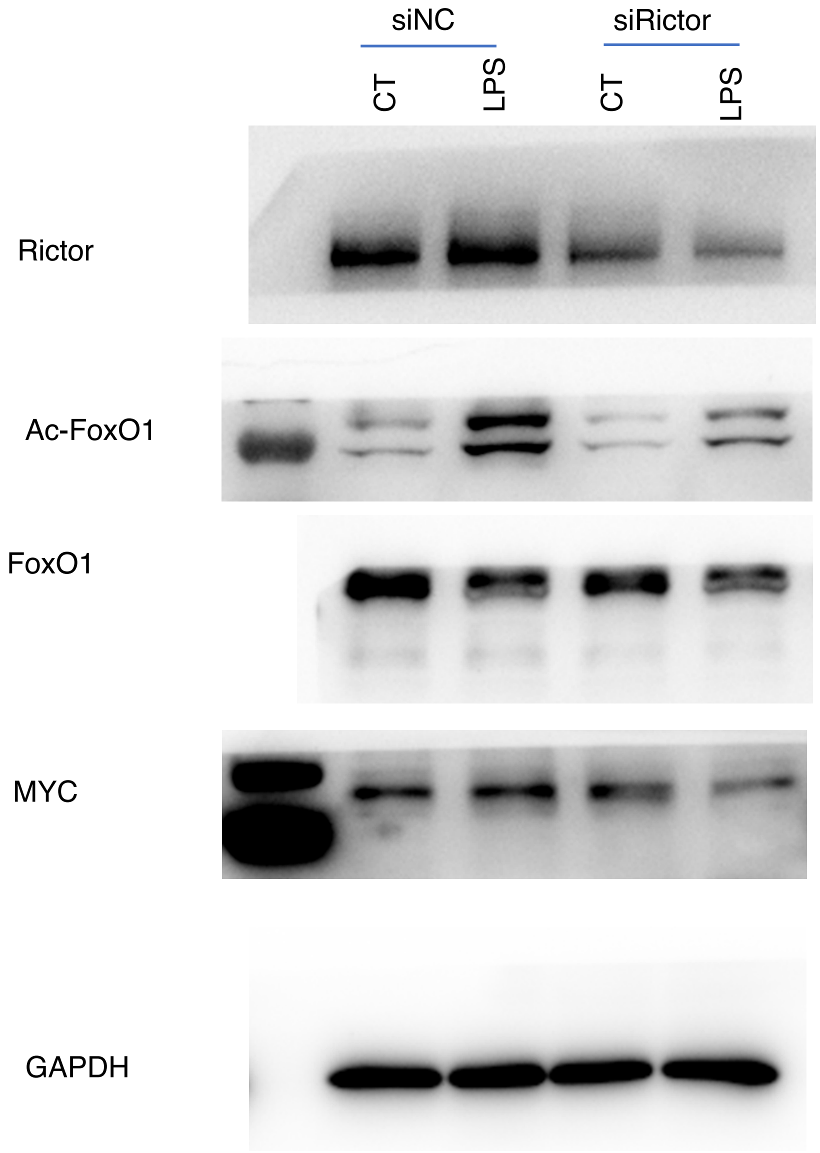

Figure 7J

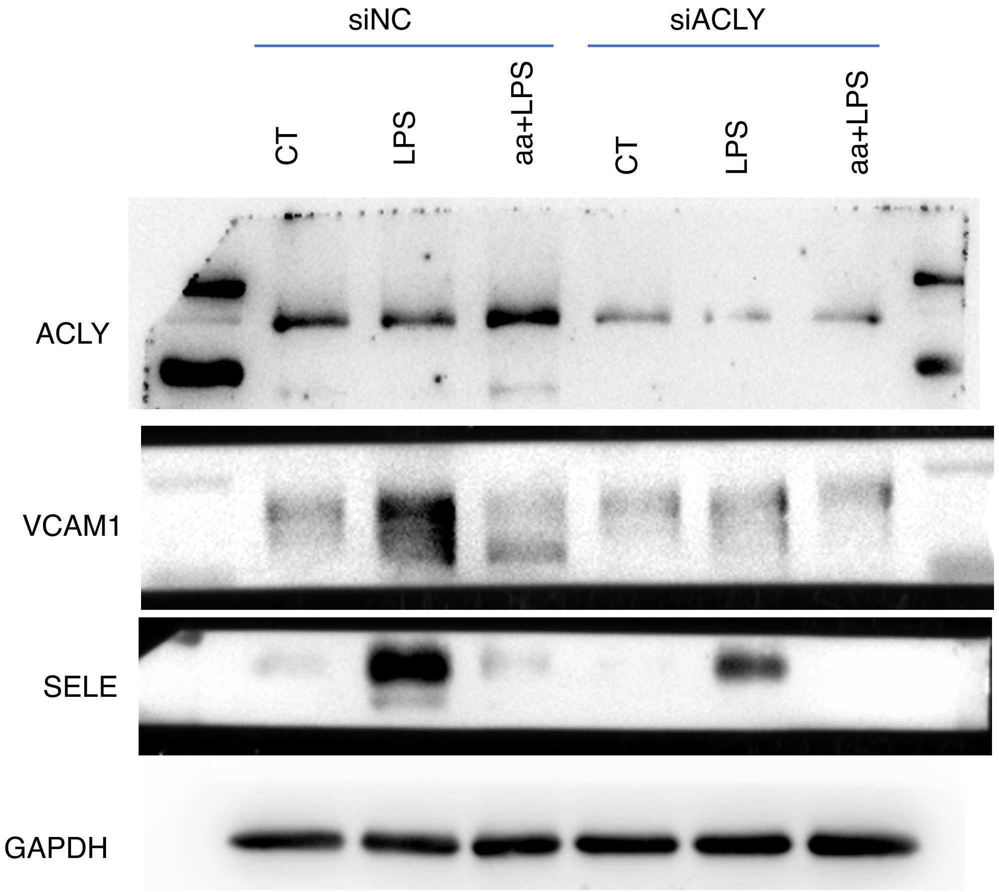

Figure 7M

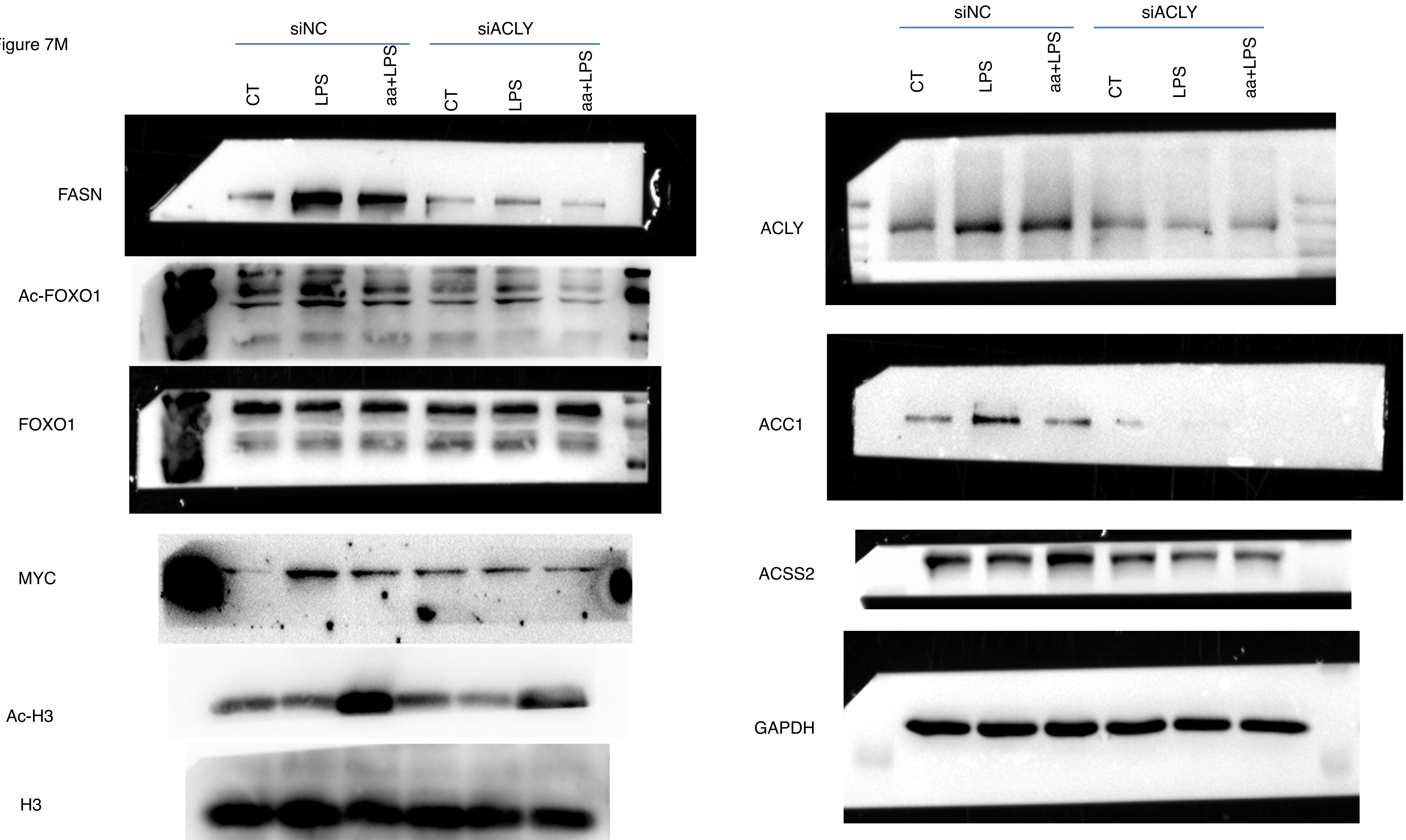
siNC
siACLY
CT
LPS
aa+LPS
CT
LPS
aa+LPS
FASN
Ac-FOXO1
FOXO1
MYC
Ac-H3
H3
siNC
siACLY
CT
LPS
aa+LPS
CT
LPS
aa+LPS
ACLY
ACC1
ACSS2
GAPDH

Figure S3A

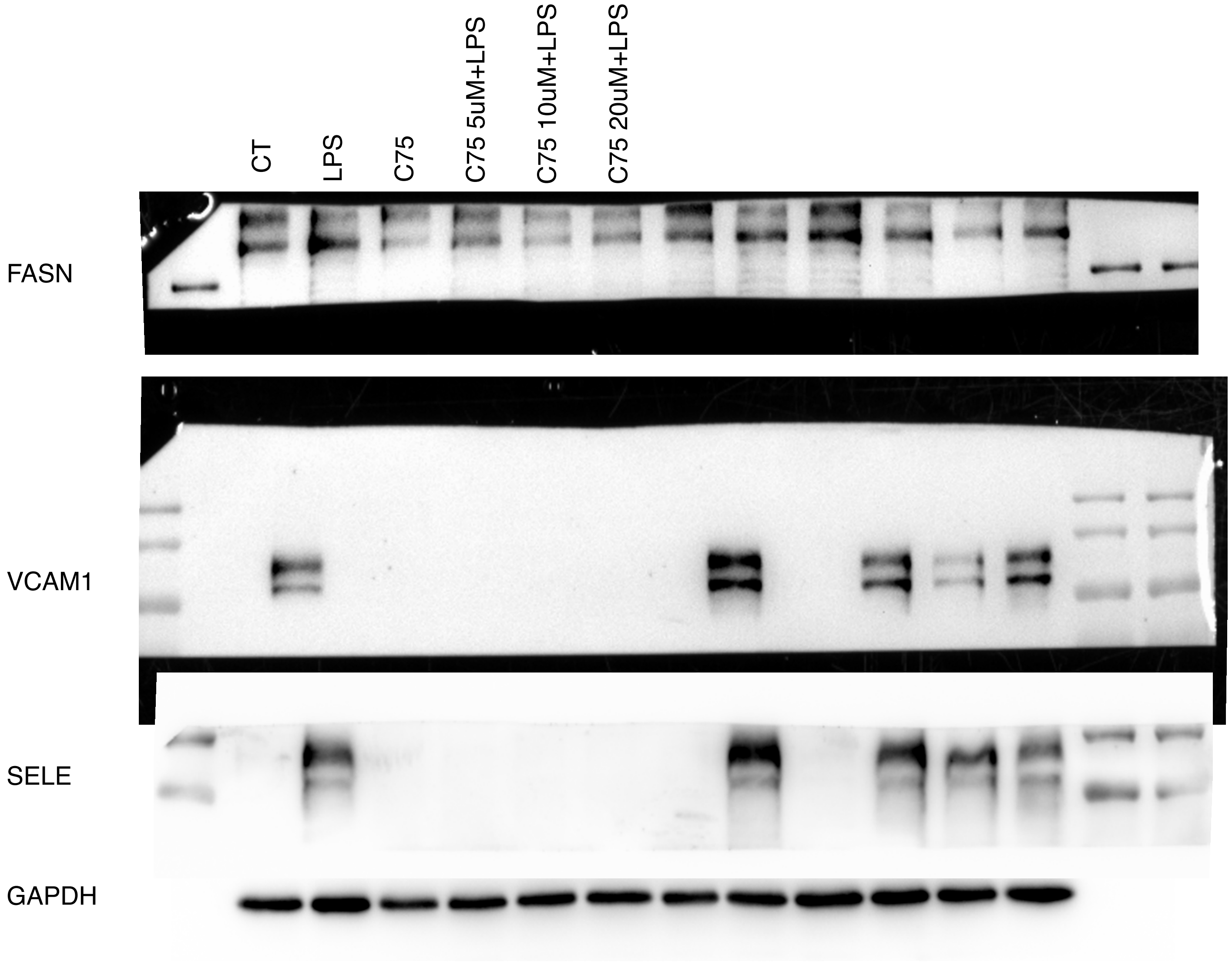
CT
LPS
C75
C75 5uM+LPS
C75 10uM+LPS
C75 20uM+LPS
FASN
VCAM1
SELE
GAPDH

Figure S4A

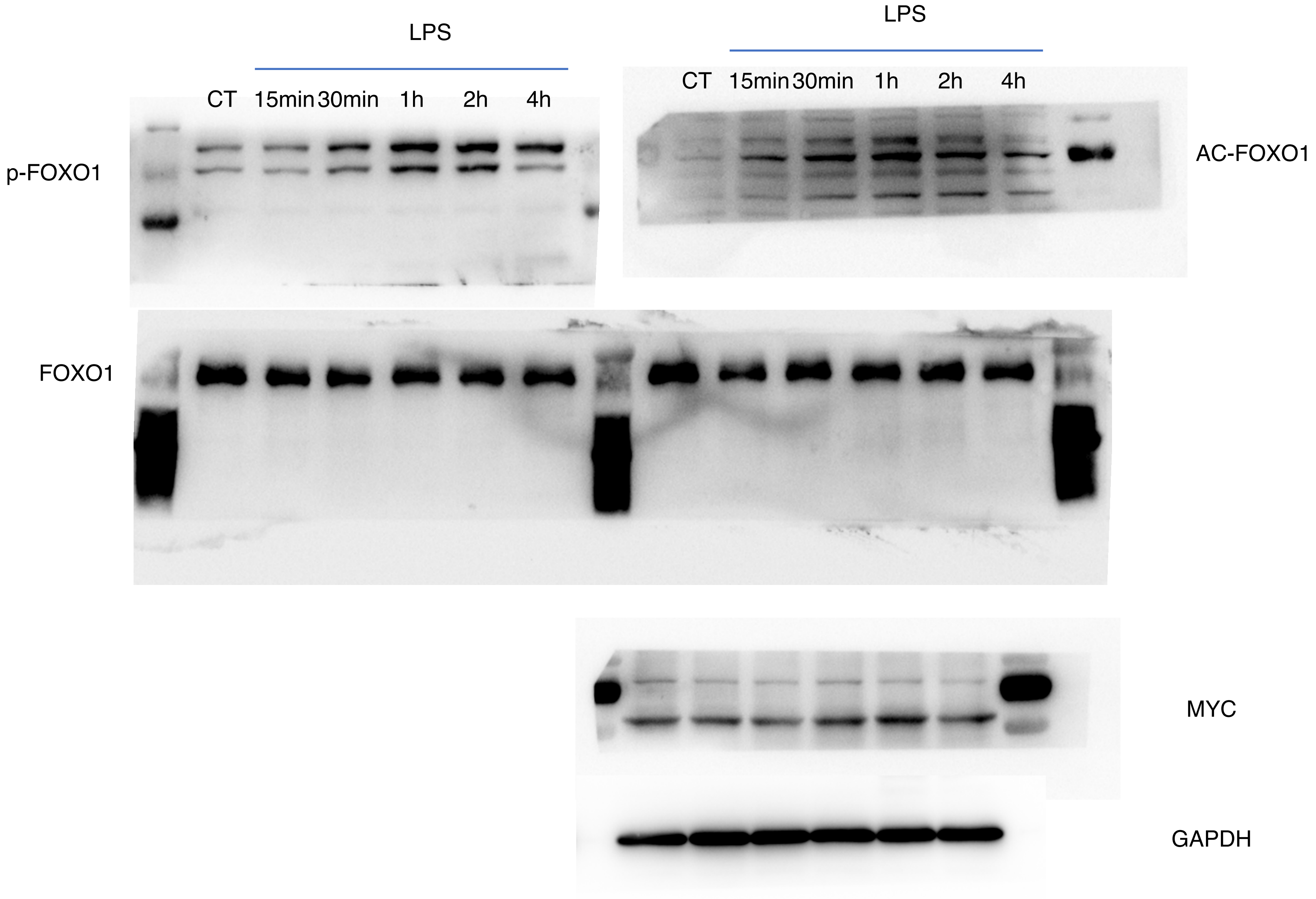
LPS
CT
15min
30min
1h
2h
4h
p-FOXO1
LPS
CT
15min
30min
1h
2h
4h
AC-FOXO1
FOXO1
MYC
GAPDH

Figure S4E

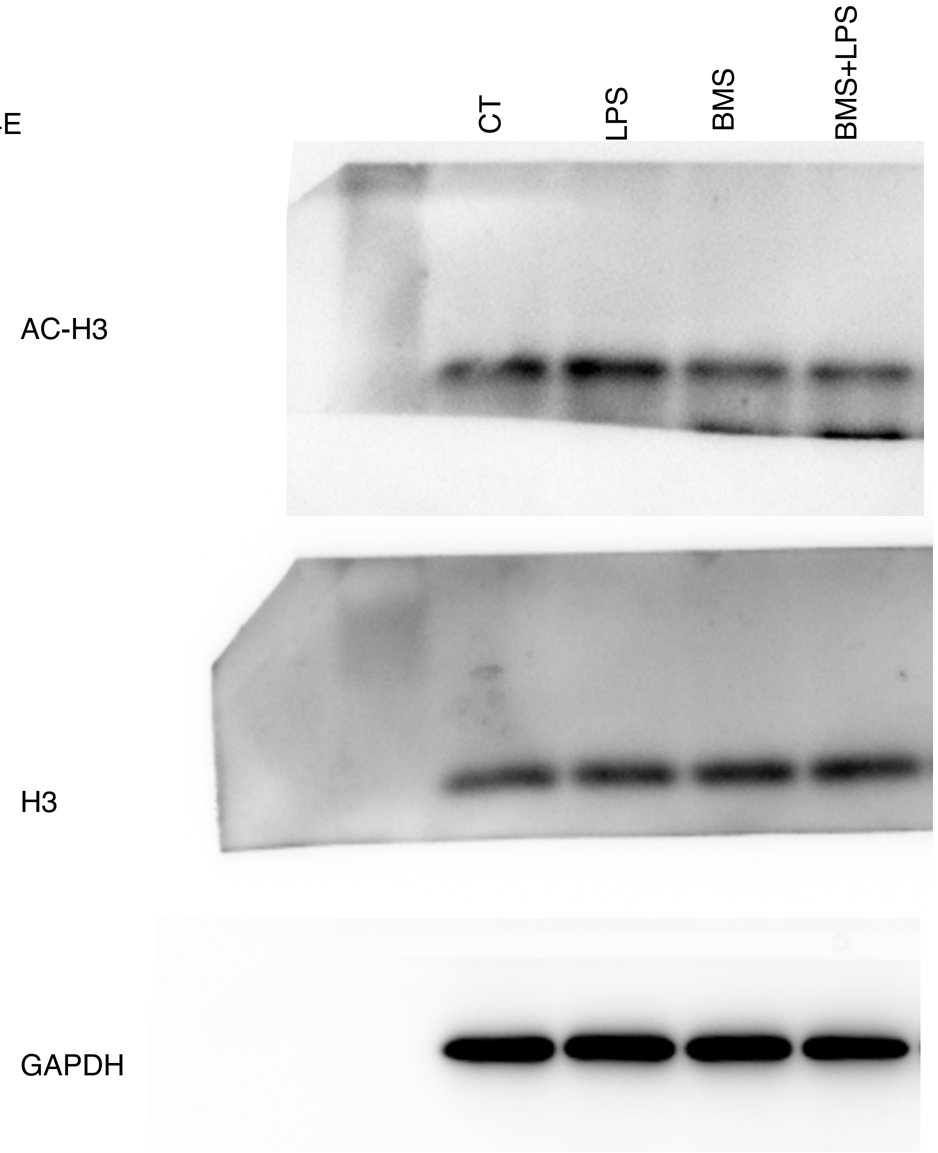

Figure4G

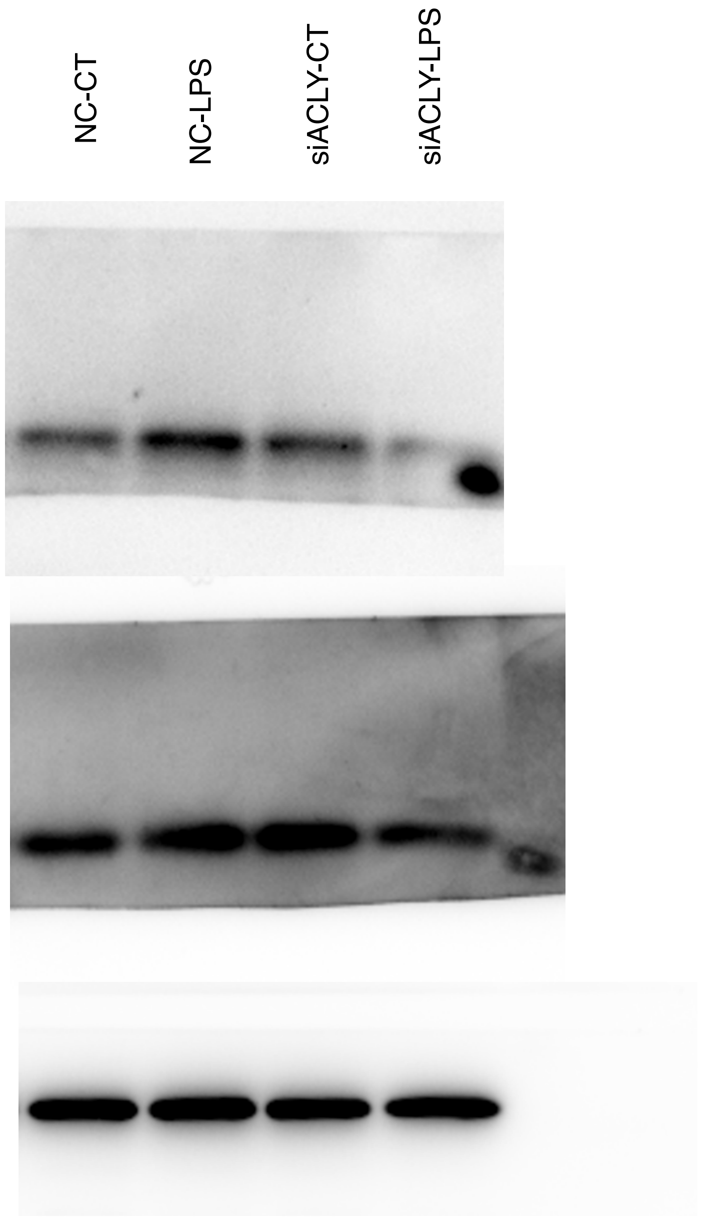

Figure S4I

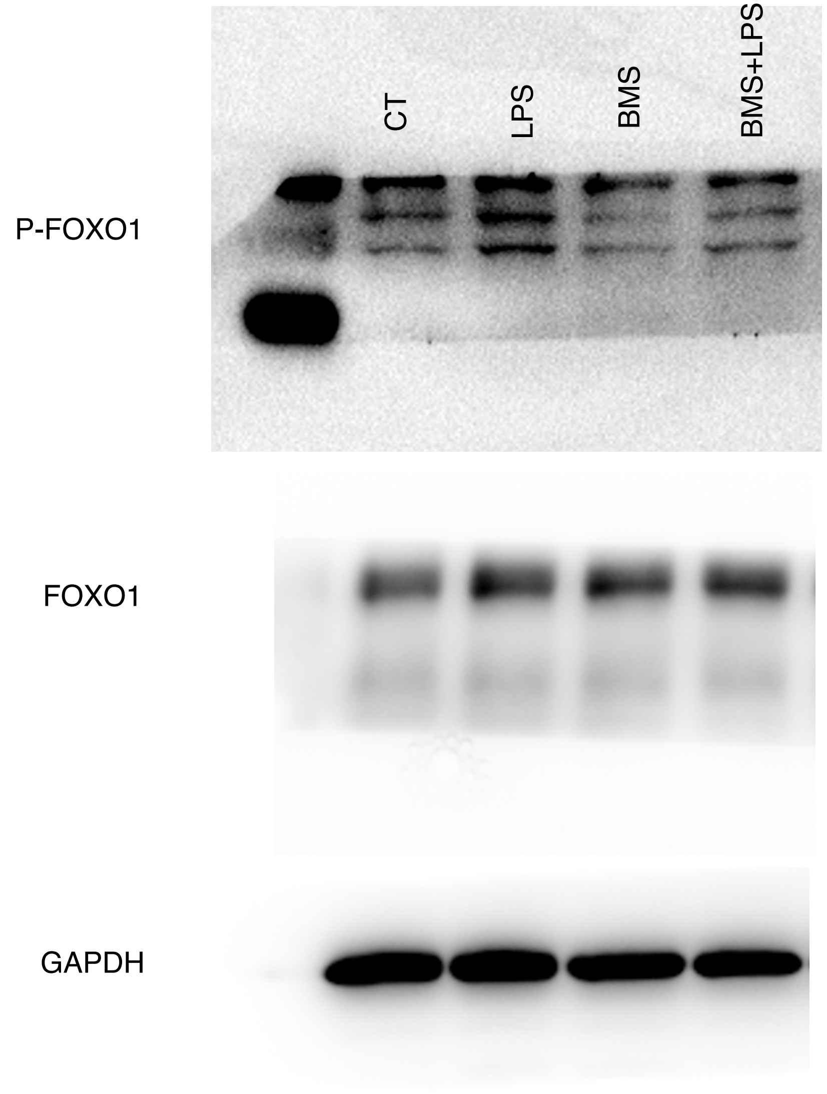

Figure S4K

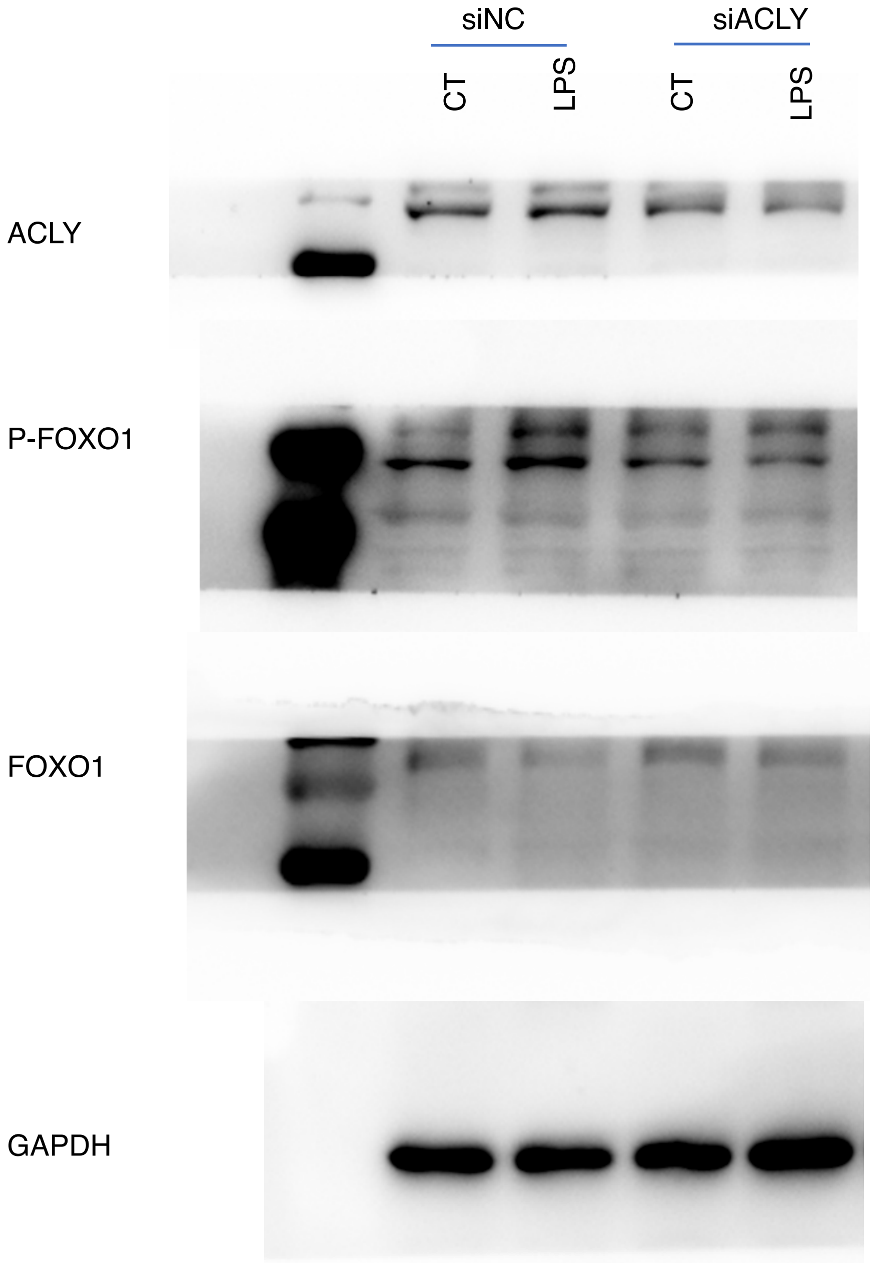

Figure S5A

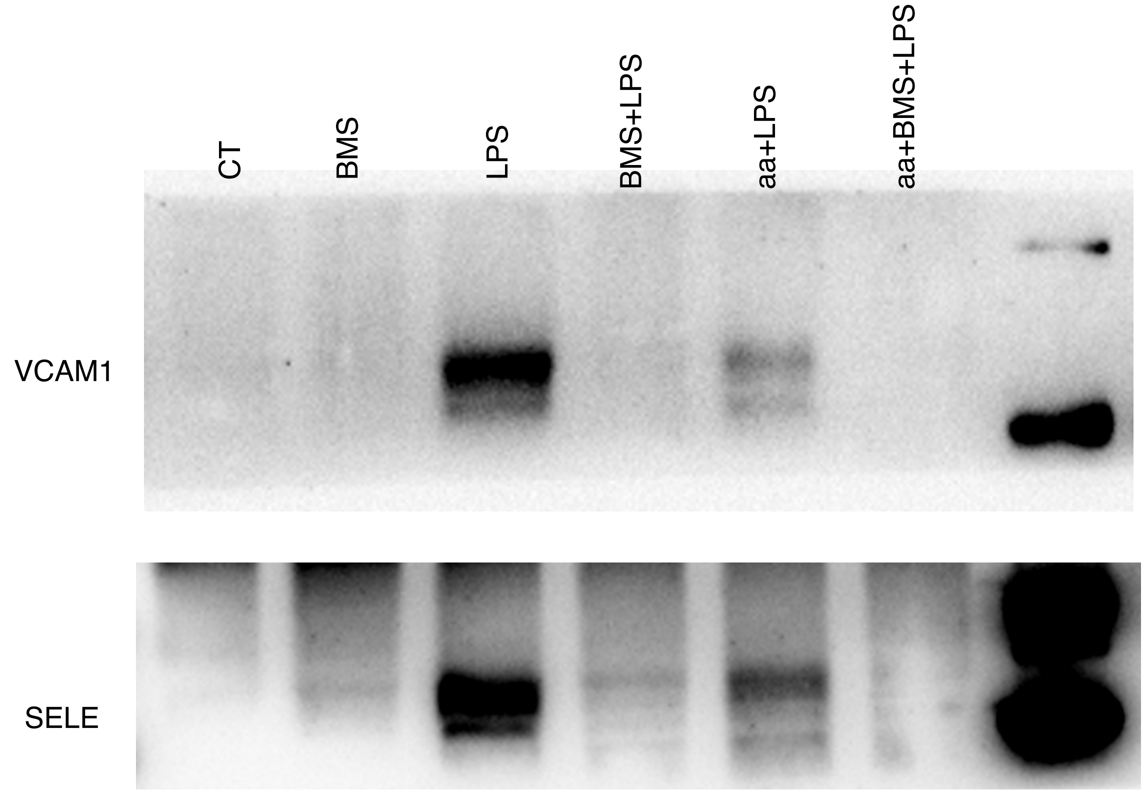
CT
BMS
LPS
BMS+LPS
aa+LPS
aa+BMS+LPS
VCAM1
SELE

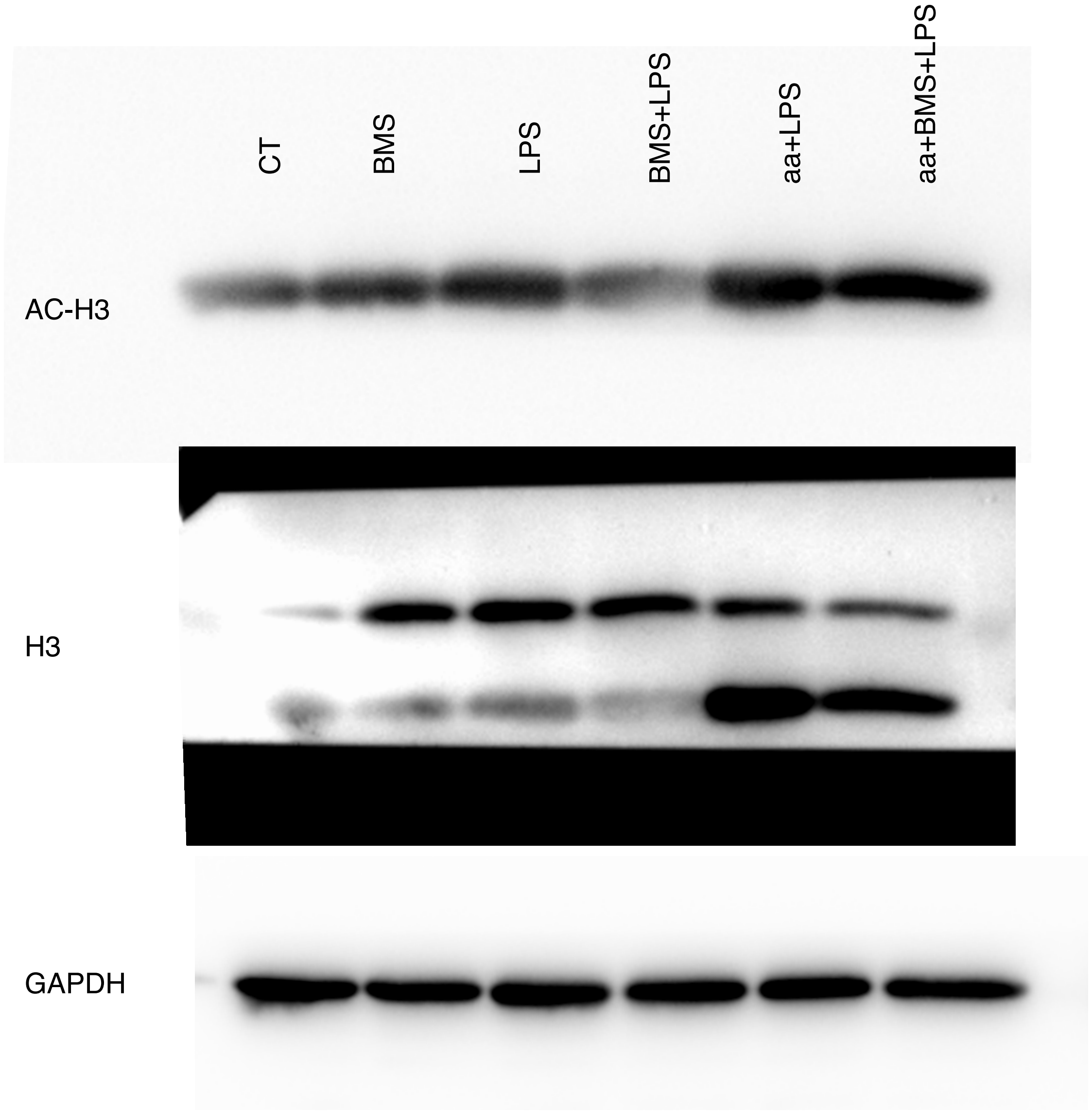
CT
BMS
LPS
BMS+LPS
aa+LPS
aa+BMS+LPS
AC-H3
H3
GAPDH

Figure S5D

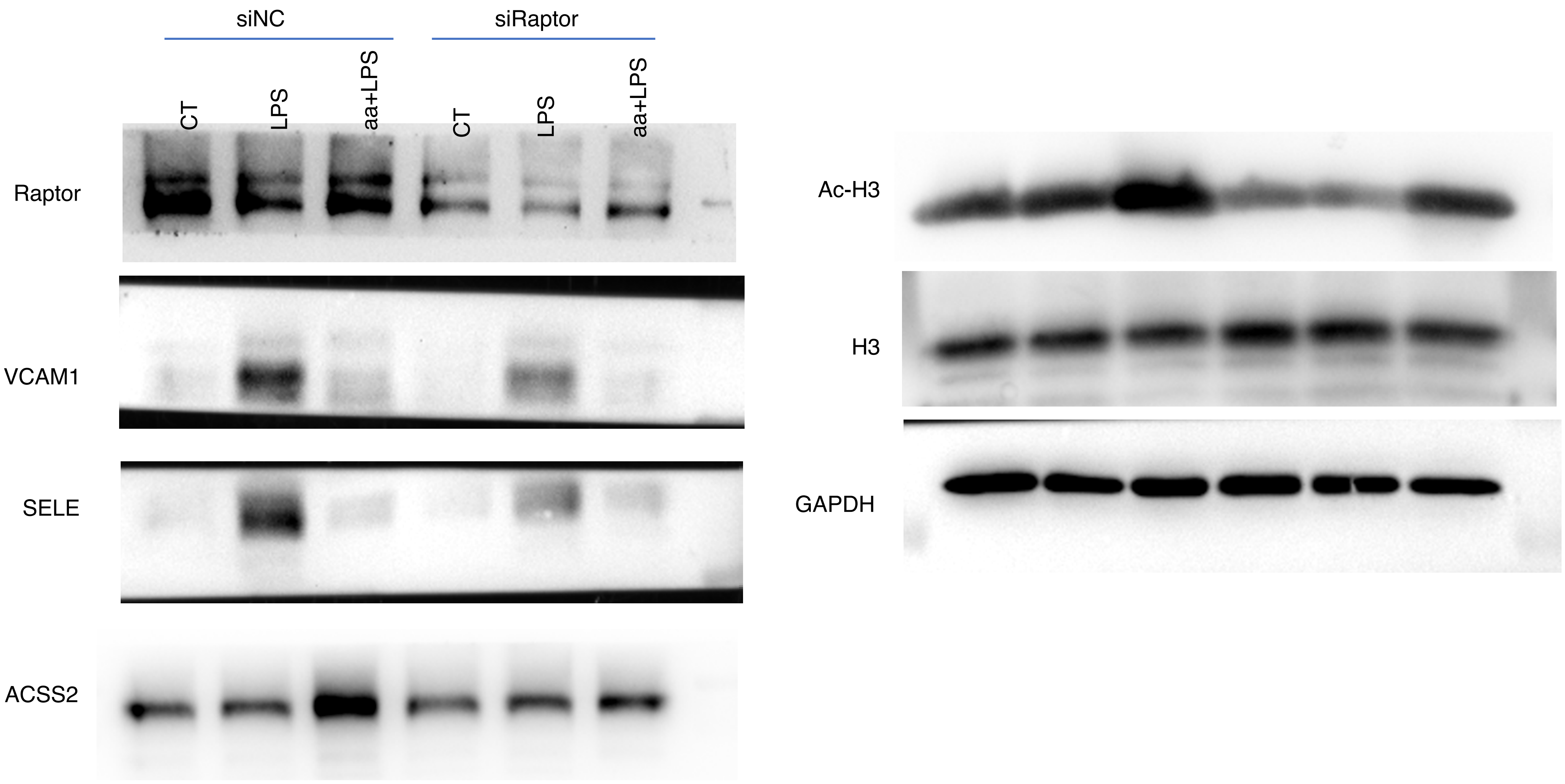
siNC
siRaptor
CT
LPS
aa+LPS
CT
LPS
aa+LPS
Raptor
VCAM1
SELE
ACSS2
Ac-H3
H3
GAPDH

Figure S5G

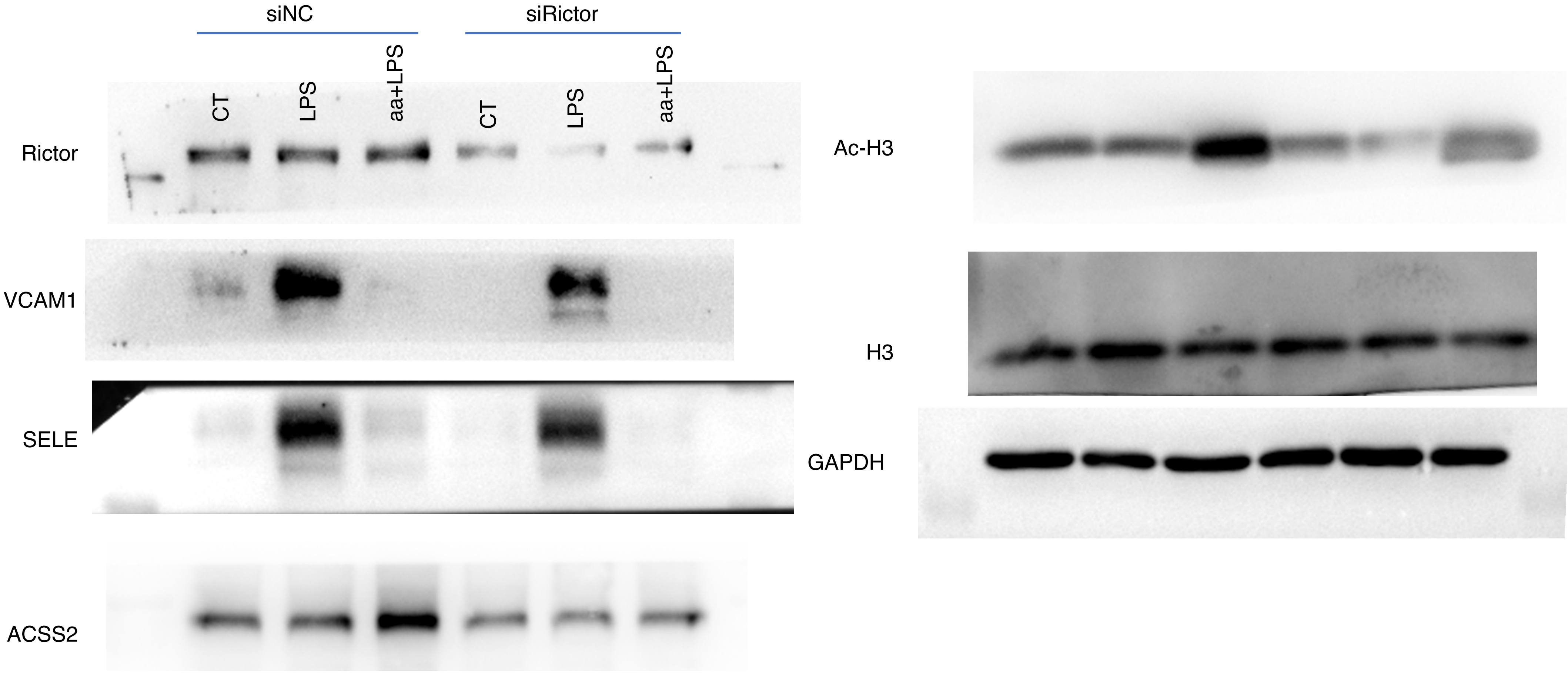
siNC
siRictor
CT
LPS
aa+LPS
CT
LPS
aa+LPS
Rictor
VCAM1
SELE
ACSS2
Ac-H3
H3
GAPDH
